# Developing a Highly Efficient and Magnetically Recoverable Nanocatalyst for Glycolytic Depolymerization of Various Polyesters

**DOI:** 10.1021/acssuschemeng.5c01220

**Published:** 2025-05-23

**Authors:** Carmen Martín, Maite Perfecto-Irigaray, Garikoitz Beobide, Elena Solana-Madruga, David Ávila-Brande, Marcos Laso-Quesada, Imanol de Pedro, Francisco A. Casado-Carmona, Rafael Lucena, Soledad Cardenas, Israel Cano

**Affiliations:** † Departamento de Química Inorgánica, Universidad Complutense de Madrid, Madrid 28040, Spain; ‡ Departamento de Química Orgánica e Inorgánica, Universidad del País Vasco, UPV/EHU, Apartado 644, E-48080 Bilbao, Spain; § ISIS Neutron and Muon Source, STFC Rutherford Appleton Laboratory, Didcot OX11 0QX, U.K.; ∥ BCMaterials, Basque Center for Materials, Applications and Nanostructures, UPV/EHU Science Park, E-48940 Leioa, Spain; ⊥ CITIMAC, Facultad de Ciencias, Universidad de Cantabria, 39005 Santander, Spain; # Affordable and Sustainable Sample Preparation (AS_2_P) Research Group, Departamento de Química Analítica, Instituto Químico para la Energía y el Medioambiente (IQUEMA), Universidad de Córdoba, Campus de Rabanales, Edificio Marie Curie, 14071 Córdoba, Spain; ∇ Department de Química, Facultat de Ciències, Universitat de les Illes Balears, Illes Balears, Carretera de Valldemossa Km 7.5, E-07122 Palma de Mallorca, Spain

**Keywords:** depolymerization, glycolysis, ionic liquid, magnetite, nanoparticles, polyesters, zinc

## Abstract

The synthesis of a new recyclable magnetic catalyst consisting of silica-coated magnetite nanoparticles (Fe_3_O_4_@SiO_2_) with a zinc-containing ionic liquid anchored to the surface is described. An in-depth characterization was performed using different techniques, which demonstrated that Fe_3_O_4_@SiO_2_@(mim)­[ZnCl­(OH)_2_] (mim: methylimidazolium) depicts the actual structure of the nanocatalyst. This system exhibits an outstanding performance as a magnetically recoverable catalyst for the glycolysis of different polyesters in ethylene glycol, such as polyethylene terephthalate (PET), poly­(1,4-butylene terephthalate) (PBT), and bisphenol A polycarbonate (BPA-PC). The depolymerization of PET and PBT into bis­(2-hydroxyethyl)­terephthalate (BHET) was carried out with nearly 100% selectivity and yield over 12 reaction cycles at 170 °C without tedious workup or purification processes. Similar behavior was observed in the glycolysis of BPA-PC into bisphenol A (BPA), which was obtained with more than 80% yield during 12 consecutive runs. Indeed, the nanocatalyst remained active with only a small loss of activity in the 20th cycle of recovery and reuse, demonstrating the high potential of this catalytic system for the chemical recycling of plastics. Besides, the unique catalytic and magnetic properties of this hybrid material have allowed us to develop gram-scale experiments. Finally, an in-depth characterization of the recovered catalyst showed that its overall structure was preserved after the glycolysis process. Only a loss of Cl^–^ ions of the Zn-based ionic liquid, caused by a ligand exchange process with ethylene glycol species and OH^–^ ions, was observed.

## Graphical abstract

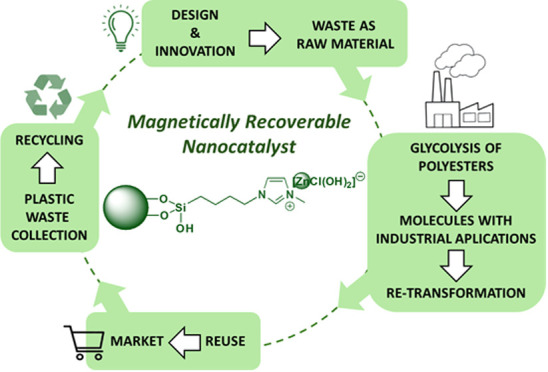

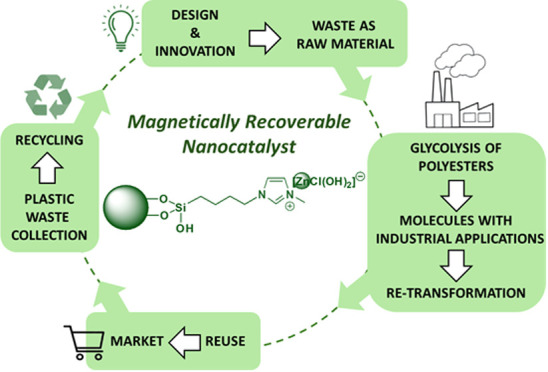

## Introduction

Plastics made from nonrenewable fossil fuels have become ubiquitous materials in our daily life, with a global production of *ca*. 359 million tons in 2018.^1–3^ These nondegradable polymers are an important environmental problem, with a prediction of accumulated plastic in nature up to 12,000 Mt by 2050.^4,5^ Consequently, advances in new biodegradable plastics and more efficient recycling approaches have become important research areas.^6^ In particular, polyesters are widely used, low-cost thermoplastics that represent more than 10% of the global plastic market. Some examples of polyesters are poly­(1,4-butylene terephthalate) (PBT), employed in the development of electronic instruments or automotive parts materials,^7,8^ polyethylene terephthalate (PET), used as packaging materials, adhesives and fibers,^9,10^ or bisphenol A polycarbonate (BPA-PC), with applications in optic (DVDs) and electronic devices (Figure 1).^11^

**Figure 1:**
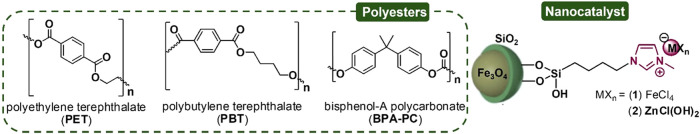
Repeating units of selected polyesters (left) and the scheme of nanocatalysts Fe_3_O_4_@SiO_2_@(mim)­[FeCl_4_] (**1**) and Fe_3_O_4_@SiO_2_@(mim)­[ZnCl­(OH)_2_] (**2**) (right).

Among the different strategies for the recycling of polyesters,^12–15^ catalytic glycolysis is considered as a low-cost and powerful chemical recycling method, which is even used in industry (*e*.*g*., IBM and Ioniqa).^16–19^ In the glycolysis process, a polyester is depolymerized to its monomer via transesterification reaction in the presence of glycol.^20^ Interestingly, the obtained monomer can be repolymerized or used as a chemical precursor in the synthesis of fine chemicals.^21,22^ Many types of catalysts have been employed to accelerate this transformation, decrease the reaction temperature, and make the process more selective toward the monomers. Commonly used catalysts^13,23^ are metal salts,^24,25^ metal oxides,^26^ metal–organic frameworks (MOFs),^27^ ionic liquids (ILs),^28^ deep eutectic solvents,^29,30^ metal nanoparticles,^31^ organocatalysts,^32,33^ and microbial agents.^34^ Among them, the most attractive catalysts are those that can be efficiently recovered to be reused, such as ultrasmall cobalt nanoparticles,^35^ Fe_3_O_4_-boosted multiwalled carbon nanotubes,^36^ iron catalyst immobilized on bentonite,^37^ or metal oxides supported on alumina (10%Ce/Al_2_O_3_).^38^ In the current realm, magnetically recoverable catalysts have become a smart alternative to tedious purification techniques (*e*.*g*., filtration and vacuum distillation). Indeed, the past decade has seen an increase in the development of this type of catalytic systems. To give recent examples, magnetic CoFe_2_O_4_,^39,40^ Mg–Al–O@Fe_3_O_4_,^41^ or Co–Al31@Fe_3_O_4_ ^42^ particles led to quantitative polymer degradation and monomer yields between 72 and 99%, also allowing the magnetic recycling of the catalyst for its subsequent reuse.

In this context, we have recently become interested in the development of new ILs-based catalytic systems for polyesters degradation.^43,44^ In particular, metal-containing ILs, having a Lewis acid and a nucleophile within the structure, are quite active for PET glycolysis.^45^ In a recent contribution, we described the synthesis of a multifunctional material based on silica-coated magnetite (Fe_3_O_4_@SiO_2_) nanoparticles (NPs) with an iron-containing ionic liquid catalyst (mim)­[FeCl_4_] anchored on the surface (Figure 1, NPs **1**, MX*_n_* = FeCl_4_).^46^ These functionalized NPs showed an outstanding performance as a magnetically recoverable catalyst for the glycolysis of PET into bis­(2-hydroxyethyl) terephthalate (BHET) in ethylene glycol (EG) due to the cooperative effect of the magnetic core and the IL-based catalytic coating. This catalytic system led to nearly 100% yield and selectivity over 12 consecutive reaction cycles at 180 °C. Inspired by these results, we set out to extend this approach to a new synthesized magnetically recoverable nanocatalyst with a zinc-containing IL anchored on the surface, Fe_3_O_4_@SiO_2_@(mim)­[ZnCl­(OH)_2_] NPs (Figure 1, NPs **2**). We have considered this nanomaterial as a promising candidate for catalytic applications in polyesters degradation, as zinc-based ILs exhibit among the highest catalytic activity for the glycolysis of PET (conversion = 99.6%, selectivity = 77.4%, *T* = 170 °C) compared to similar ILs containing other metals (*e*.*g*., Cr, Mn, Fe, Co, Ni).^47^

Important advances have recently been reported on magnetically recoverable catalysts based on a magnetite core^41,42^ and analogous catalysts coated by ILs.^17–19,48,49^ For example, Fe_3_O_4_@PMIM.SbBr_4_ nanoparticles (PMIM: propylmethylimidazolium) with antimony­(III) bromide on the surface (BHET yield = 96.4%, 200 °C, catalyst loading = 6.0 wt %, 5 cycles),^48^ or silica-coated magnetite NPs (Fe_3_O_4_@SiO_2_) functionalized with ILs and Fe^3+^ ions, and with Fe_2_O_3_ immobilized on the surface (BHET yield = 73.0%, 190 °C, catalyst loading = 10.0 wt %, 5 cycles).^49^ However, these catalytic systems are not able to compete with the high activity (BHET yield = 99%, 170 °C, catalyst loading = 6 wt %, Zn-containing IL loading = 0.39 mol %), versatility (degradation of various polyesters: PET, PBT, and BPA-PC), and recyclability (up to 20 cycles!) of the nanocatalyst described herein, Fe_3_O_4_@SiO_2_@(mim)­[ZnCl­(OH)_2_] (**2**). The synergistic effect of the magnetic core and the Zn-based catalytic coating, together with its high thermal stability, turns this nanocatalyst into one of the most active reported to date for polyesters depolymerization. Indeed, **2** shows comparable activity to those exhibited by similar IL catalysts based on Fe, Al, Ca, and Cu immobilized on magnetic supports developed by Ioniqa.^17–19^ It is also worth noting the exhaustive characterization of the fresh and recovered catalysts, which has allowed us to gain insight into the catalyst evolution during the depolymerization process.

First, the synthesis and characterization of the catalytic system (**2**) will be presented in detail. Then, special attention will be paid to the optimization of the glycolysis reaction conditions. Subsequently, we will show the catalytic behavior and magnetic recycling of these NPs for the degradation of several polyesters (PET, PBT, and BPA-PC). Finally, the facile magnetic recovery and successive reuse of the catalyst for up to 20 cycles in the glycolysis of BPA-PC will be presented.

## Materials and Methods

### General Procedures

All solvents were purchased from Sigma-Aldrich. PBT (average Mv ≈ 38,000) and BPA-PC pellets were also supplied by Sigma-Aldrich (ref 190945 and ref 435139, respectively). Commercial PET sourced from water bottles obtained from a local market and cut to an approximate size of *ca*. 5 mm was employed in the catalytic degradation.

### Instrumentation

#### Thermogravimetric Analysis (TGA)

Thermogravimetric measurements were carried out on a TA Instruments, SDT Q600 model, at a heating rate of 10 °C/min in a temperature range from 25 to 800 °C under synthetic air in a platinum crucible. Decomposition temperatures (*T*_d_) at 10% weight loss of the analyte were determined by TGA for BHET and BPA.

#### Differential Scanning Calorimetry (DSC)

DSC measurements were carried out on a DSC Q20 TA Instruments, from 0 to 150 or 175 °C at a rate of 10 °C/min under nitrogen atmosphere. Glass transition temperatures (*T*_g_) are determined by DSC.

#### X-ray Powder Diffraction (XRPD)

Laboratory XRPD studies were performed in an air atmosphere on a PANalytical X′Pert Powder diffractometer, using Cu Kα radiation and an X’celerator CCD detector. Diffraction patterns were collected in the 5–70° 2θ angular range with a 0.04° step size and Bragg–Brentano geometry. High-resolution patterns for refinement were collected in a 0.3 mm borosilicate capillary in transmission mode using a PANalytical X′Pert Pro α 1 diffractometer with monochromatic Cu Kα_1_ radiation. An elliptical mirror was used in the detector to optimize the signal, collected in the 5° < 2θ < 120° angular range with a 0.1° step size.

#### X-ray Energy-Dispersive Spectroscopy (XEDS) Mapping

The XEDS maps of the samples were carried out using scanning transmission electron microscopy (STEM) combined with XEDS mapping. The measurements were performed on a JEOL 3000 F microscope equipped with a high-angle annular dark-field (HAADF) detector for Z-contrast imaging and an Oxford Instruments XEDS detector (OXFORD INCA) for elemental analysis. The XEDS spectra were collected using an acquisition time of approximately 5 s per pixel to ensure adequate signal-to-noise ratios.

#### High-Resolution Transmission Electron Microscopy (HR-TEM)

Low-magnification and high-resolution transmission electron microscopy (HR-TEM) studies were carried out with a JEM 3000F microscope operating at 300 kV (double tilt (±20°), point resolution 1.7 Å) fitted with an XEDS microanalysis system (OXFORD INCA). The samples were ground in *n*-butyl alcohol and ultrasonically dispersed. A few drops of the resulting suspension were deposited on a carbon-coated grid.

#### X-ray Photoelectron Spectroscopy (XPS)

XPS analyses of fresh and recovered catalyst samples were performed using a Phoibos 150 1D-DLD (SPECS) energy analyzer equipped with a Focus 500 monochromatic radiation source, an Al/Ag dual anode, and an SED-200 secondary electron detection system. Prior to measurements, the catalyst sample recovered from the polyester glycolysis process was washed with water to remove remnant EG and reagents from the surface and dried in an oven at 100 °C for 5 h. Alternatively, the catalyst sample recovered after heating in the presence of EG and in the absence of polymer was washed with dichloromethane and dried in an oven at 170 °C for 24 h under a vacuum. The data treatment was performed using CasaXPS software (version 2.3.26).^50^

#### Magnetic Susceptibility Measurements

Variable-temperature magnetic susceptibility measurements were performed using an MSPS-XL SQUID magnetometer from 2 to 300 K at magnetic fields of 0.1 and 0.5 kOe both in FC and ZFC modes. A field-dependent magnetization loop at 2 K was measured between −5 and 5 T.

#### Sorption Measurements

Nitrogen physisorption data were collected at 77 K using a Quantachrome Autosorb-iQ MP analyzer. Prior to the measurements, the samples were outgassed under vacuum at 140 °C for 6 h. The surface area values were calculated by fitting the nitrogen adsorption data to the Brunauer–Emmett–Teller (BET) equation.^51^ The mean particle size was approached according to the expression *S* = 6/*d*_p_·ρ, which relates the specific surface area (*S*) with the diameter of spherical particles (*d*_p_) and their density (ρ).

#### Fourier Transform Infrared Spectroscopy (FT-IR)

FT-IR measurements of products were performed on a PerkinElmer Spectrum 100 FT-IR spectrometer equipped with an attenuated total reflectance (ATR) module. The ATR FT-IR spectra were recorded by collecting 24 scans in the ATR module.

#### Nuclear Magnetic Resonance (NMR)

^1^H and ^13^C NMR spectra of the products were recorded on a Bruker Avance 300 MHz nuclear magnetic resonance spectrometer. Chemical shifts of ^1^H and ^13^C NMR are reported in ppm. Signals are quoted as s (singlet), t (triplet), or m (multiplet).

### Synthesis of the Magnetically Recoverable Nanocatalyst (**2**)

Magnetite (Fe_3_O_4_) NPs were prepared in four stages according to the literature procedure.^52^ Afterward, the synthesized Fe_3_O_4_ NPs were coated with silica (SiO_2_) by reaction with tetraethylorthosilicate (TEOS) to afford silica-coated magnetite NPs (Fe_3_O_4_@SiO_2_).^53^ Then, Fe_3_O_4_@SiO_2_ NPs were functionalized with methylimidazolium-chloride ((mim)­Cl).^54^ Finally, the obtained Fe_3_O_4_@ SiO_2_@(mim)Cl NPs (2 g) were dispersed in an aqueous ZnCl_2_ solution (100 mL, 7% (w/v)) and stirred for 48 h to generate the zincate ionic ensemble ((mim)­[ZnCl­(OH)_2_]) on the surface of the nanoparticles via the substitution of Cl^–^ by the zincate anion. The synthesized NPs (**2**) were sequentially washed with Milli-Q water (50 mL) and methanol (50 mL), and dried at 80 °C for 8 h. Elemental analysis (%): C, 2.66; H, 0.91; N, 0.79. ICP (%): Fe, 54.0; Zn, 2.2. TGA: 7.09% mass loss at 700 °C.

### Catalytic Experiments

#### General Procedure for Catalytic Degradation of Polyesters

6 mg of catalyst, 100 mg of polyester, and 1 mL of EG were placed in a 10 mL round-bottom flask equipped with a reflux condenser. The mixture was heated under reflux at 180 °C (170 °C inside of the reaction flask). After 24 h, the flask was cooled down to room temperature (r.t.), and 10 mL of distilled H_2_O (for PET and PBT) or a mixture of H_2_O:EtOH 1:1 (for BPA-PC) was added to the reaction crude. The catalyst was separated from the liquid phase with an external magnet and washed with distilled water (for PET and PBT) or a mixture of H_2_O:EtOH 1:1 (for BPA-PC) two times. The portions were combined, and the undepolymerized polyester was separated from the resulting mixture by filtration and dried at 80 °C. The liquid phase was evaporated using a rotary evaporator at 40 °C to remove H_2_O. An aliquot of the residue (100 mg) was dissolved in DMSO-*d*_6_ and analyzed by ^1^H NMR to determine the amount of product using 1-phenyl-1,2-ethanediol (0.1 mmol) as an internal standard. BHET was purified by recrystallization in water. BPA was purified by precipitation with water and subsequent filtration through silica gel using a mixture of hexane:dichloromethane (1:1) as eluent. The purity of the product was confirmed using a variety of techniques, such as ^1^H and ^13^C NMR spectroscopy, ATR FT-IR, elemental analysis (EA), DSC, and TGA (Figures S19–S33, Supporting Information).

#### BHET

^1^H NMR (300 MHz, 298 K, DMSO-*d*_6_): δ 8.12 (s, 4H, C*H*_Ar_), 4.96 (t, *J* = 5.7 Hz, 2H, – OCH_2_CH_2_O*H*), 4.32 (t, *J* = 4.8 Hz, 4H, – OC*H*_2_CH_2_OH), 3.72 (m, 4H, – OCH_2_C*H*_2_OH). ^13^C NMR (75 MHz, DMSO-*d*_6_): δ 165.2 (s, C_q_, *C*=O), 133.8 (s, C_q_, *C*_Ar_), 129.5 (s, CH, *C*H_Ar_), 67.0 (s, – O*C*H_2_CH_2_OH), 59.0 (s, – OCH_2_ *C*H_2_OH). Characteristic IR bands (cm^–1^): 3444 (O–H), 2962 (C–H), 2945 (C–H), 2930 (C–H), 2879 (C–H), 1713 (C=O), 1503–1409 (C_Ar_ = C_Ar_), 1275 (C–O), 1250 (O–H), 1070 (C–O), 909–860 (C_Ar_ = C_Ar_). *T*_d_ = 226.7 °C. *T*_g_ = 113.8 °C. Anal. for C_12_H_14_O_6_ (from PET): C 56.69, H 5.55; found: C 56.65, H 5.47. Anal. Calcd for C_12_H_14_O_6_ (from PBT): C 56.69, H 5.55; found: C 56.69, H 5.45.

#### BPA

^1^H NMR (300 MHz, 298 K, DMSO-*d*_6_): δ 9.12 (s br, 2H, O*H*), 6.97 (d, *J* = 8.7 Hz, 4H, C*H*_Ar_), 6.63 (d, *J* = 8.7 Hz, 4H, C*H*_Ar_), 1.52 (s, 6H, C*H*_3_). ^13^C NMR (75 MHz, DMSO-*d*_6_): δ 154.8 (s, C_q_, *C*_Ar_), 141.1 (s, C_q_, *C*_Ar_), 127.2 (s, CH, *C*H_Ar_), 114.5 (s, CH, *C*H_Ar_), 40.9 (s, C_q_, *C*(CH_3_)_2_(C_Ar_)_2_), 30.8 (s, CH_3_, – C­(*C*H_3_)_2_). Characteristic IR bands (cm^–1^): 3326 (O–H), 3067 (C–H), 3031 (C–H), 2964 (C–H), 2929 (C–H), 2872 (C–H), 1713 1615–1600 (C_Ar_ = C_Ar_), 1218 (C–O). *T*_d_ = 214.8 °C. *T*_g_ = 155.6 °C. Anal. for C_15_H_16_O_2_·1/4H_2_O: C 77.39, H 7.14; found: C 77.33, H 7.39.

The conversion of polymer is calculated using the following equation

1 $$\mathrm{conversion}=\frac{W_{0}-W_{1}}{W_{0}}\times 100$$

where *W*_0_ is the initial polymer weight and *W*_1_ is the weight of undepolymerized polyester.

In addition, the yield and selectivity of the monomer are defined by eqs 2 and 3

2 $$y\mathrm{ield}=\frac{\mathrm{moles of monomer}}{\mathrm{initial moles of polymer units}}\times 100$$

3 $$s\mathrm{electivity}=\frac{\mathrm{moles of monomer}}{\mathrm{moles of depolymerized polymer units}}\times 100$$

### General Procedure for Recycling Experiments

6 mg of catalyst, 100 mg of polyester, and 1 mL of EG were placed in a 10 mL round-bottom flask equipped with a reflux condenser. The mixture was heated under reflux at 180 °C (170 °C inside the reaction flask). After 24 h, the flask was cooled down to r.t., and 10 mL of distilled H_2_O (for PET and PBT) or a mixture of H_2_O:EtOH 1:1 (for BPA-PC) was added to the reaction crude. The catalyst was separated from the liquid phase with an external magnet and washed with distilled water (for PET and PBT) or a 1:1 H_2_O:EtOH mixture (for BPA-PC) two times. The portions were combined, and the conversion of the polymer and the yield and selectivity of the monomer were studied as described above. The reaction flask containing the catalyst was dried using a vacuum drying oven Lbx 52 L at 60 °C for 6 h to remove water. The catalyst was then used for the next cycle.

### General Procedure for the Scale-Up Experiment

120 mg of catalyst, 2 g of polyester, and 20 mL of EG were placed in a 50 mL round-bottom flask equipped with a reflux condenser. The mixture was heated under reflux at 180 °C (170 °C inside the reaction flask). After 24 h, the flask was cooled down to R.T. and 50 mL of distilled H_2_O (for PET and PBT) or a mixture of H_2_O:EtOH 1:1 (for BPA-PC) was added to the reaction crude. The catalyst was separated from the liquid phase with an external magnet and washed with distilled H_2_O (for PET and PBT) or a 1:1 H_2_O:EtOH mixture (for BPA-PC) two times. The portions were combined, and the conversion was investigated as previously described. The BHET monomer was isolated by crystallization from the combined aqueous solution, and BPA was purified by precipitation with water and subsequent filtration through silica gel using a mixture of hexane:dichloromethane (1:1) as eluent. These purification processes allowed us to determine the selectivity and isolated yield of BHET and BPA, respectively.

## Results and Discussion

### Synthesis and Characterization of the Magnetic Catalytic System 2

The nanocatalyst **2** was prepared following the previously described synthetic procedure (for details, see the Materials and Methods section). Subsequently, it was fully characterized using a wide range of techniques, including N_2_ adsorption–desorption measurements, HR-TEM, XEDS, XRPD, XPS, magnetic measurements, elemental analysis (EA), FT-IR, and TGA. HR-TEM analysis of **2** showed monodisperse and approximately spherical nanoparticles with an average diameter of 10 ± 2 nm (Figure 2a and Figures S1–S11, Supporting Information), and revealed a crystalline core characteristic of Fe_3_O_4_ crystals as demonstrated in Figure 2b, where the nanoparticle outlined by the dotted line shows an interplanar distance of 0.253 nm, corresponding to the <113> crystallographic plane of the spinel structure. HR-TEM images also allowed us to observe the amorphous layer of SiO_2_ on the surface of magnetite NPs, with a thickness ranging from 0.7 to 2.1 nm (Figure 2b and Figures S7–S11, Supporting Information).^46^ XRPD studies confirmed the spinel structure of the magnetite core (refined against high-resolution data in Figure S12, Supporting Information) and the presence of the amorphous SiO_2_ shell (see the bump in the background of laboratory XRPD data in Figure S12 between 15 and 25°).^55^ Additionally, the use of the Scherrer equation yielded an estimated mean NP diameter of 8.96 ± 0.13 nm for **2**.

**Figure 2:**
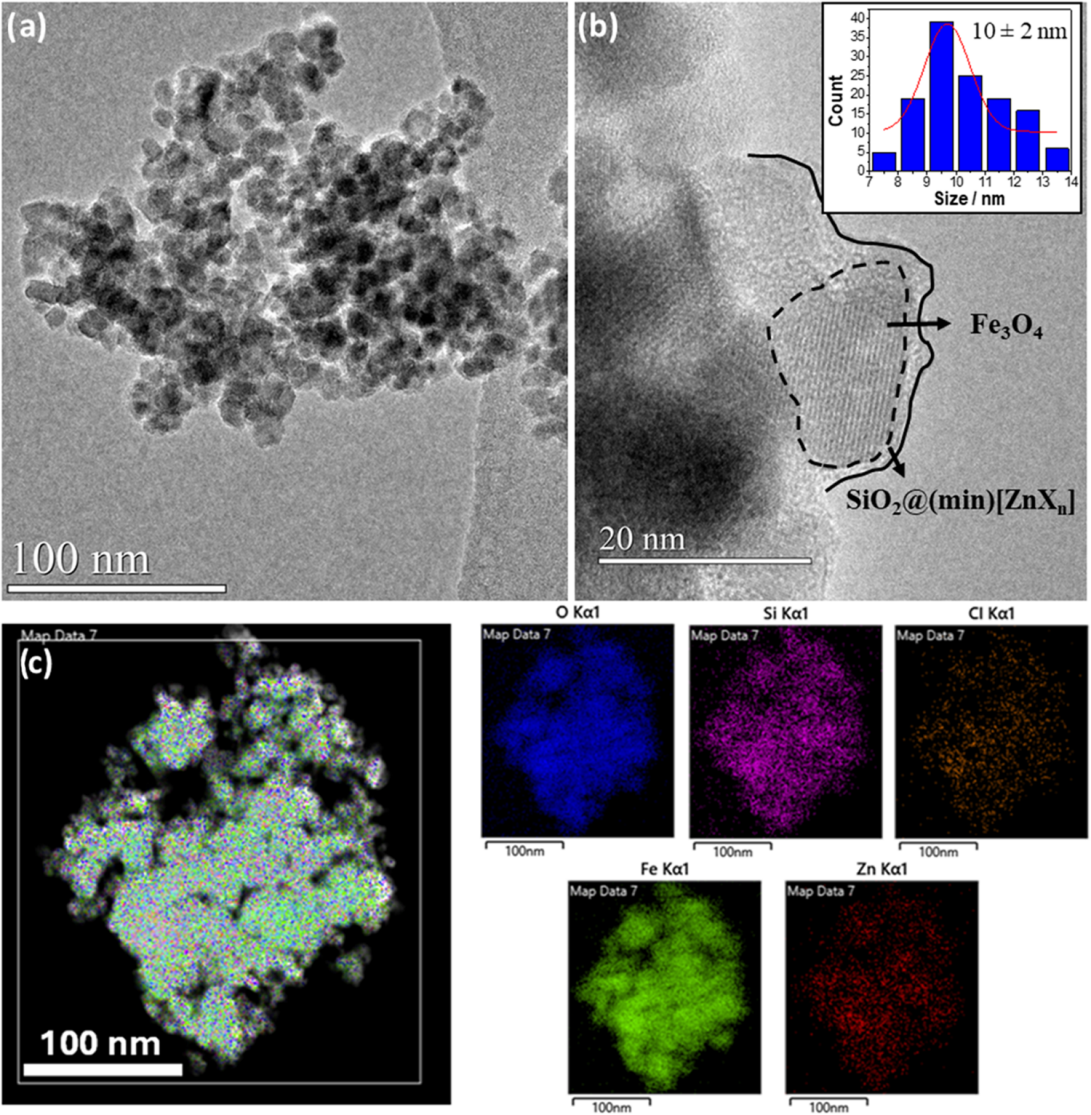
HR-TEM micrographs for **2** showing (a) a bunch of nanoparticles and (b) high magnification of the amorphous layer of SiO_2_ and the Fe_3_O_4_ core. (c) STEM-XEDS elemental map over a group of nanoparticles.

The expected ferrimagnetic behavior of magnetite was observed from susceptibility measurements (Figure S13a, Supporting Information). The characteristic irreversibility (*T*_irr_) and blocking (*T*_B_) temperatures are strongly dependent on the applied magnetic field, as in the related Fe_3_O_4_@SiO_2_@(mim)­[FeCl_4_] NPs.^46^ FC data increased upon cooling the sample below *T*_B_, which points to weak dipolar core–core interactions due to the small particle size,^56^ as confirmed by structural characterization techniques. This justifies the wasp-waisted-like feature in the low magnetic field region for the M vs H loop at 2 K, which reveals the presence of ferromagnetic domains in an antiferromagnetic matrix (Figure S13b, Supporting Information). A metamagnetic transition was noted at *H*_c_ ∼ 3*T*, above which the domains get reoriented, showing an irreversible hysteretic feature in this high magnetic field range.

Figure S14 displays the N_2_ adsorption isotherm collected at 77 K for **2**. At low relative pressures, a short adsorption step takes place until *p*/*p*° is *ca*. 0.05 (point B), after which the adsorption follows a monotonic increase related to the multilayer adsorption onto the nanoparticles of the catalyst. Thereafter, the adsorption follows a steep slope resembling a Type II curve, while the desorption branch forms an elongated hysteresis loop, resulting from capillary condensation within the interparticle macropores and mesopores. Fitting the adsorption data to BET equation (in the range *p*/*p*° = 0.05–0.30) led to a specific surface area (*S*_BET_) value of 73.7 m^2^·g^–1^ (Figure S15, Supporting Information). Since the surface area of the catalyst arises from the external area of the nanoparticles, the estimated mean particle size, using a spherical approach, was *ca*. 11 nm, in agreement with XRPD data and TEM images, which showed a size of *ca*. 9 and 10 nm for the core, respectively, and *ca*. 0.7–2.1 nm for the shell.

**Figure 3:**
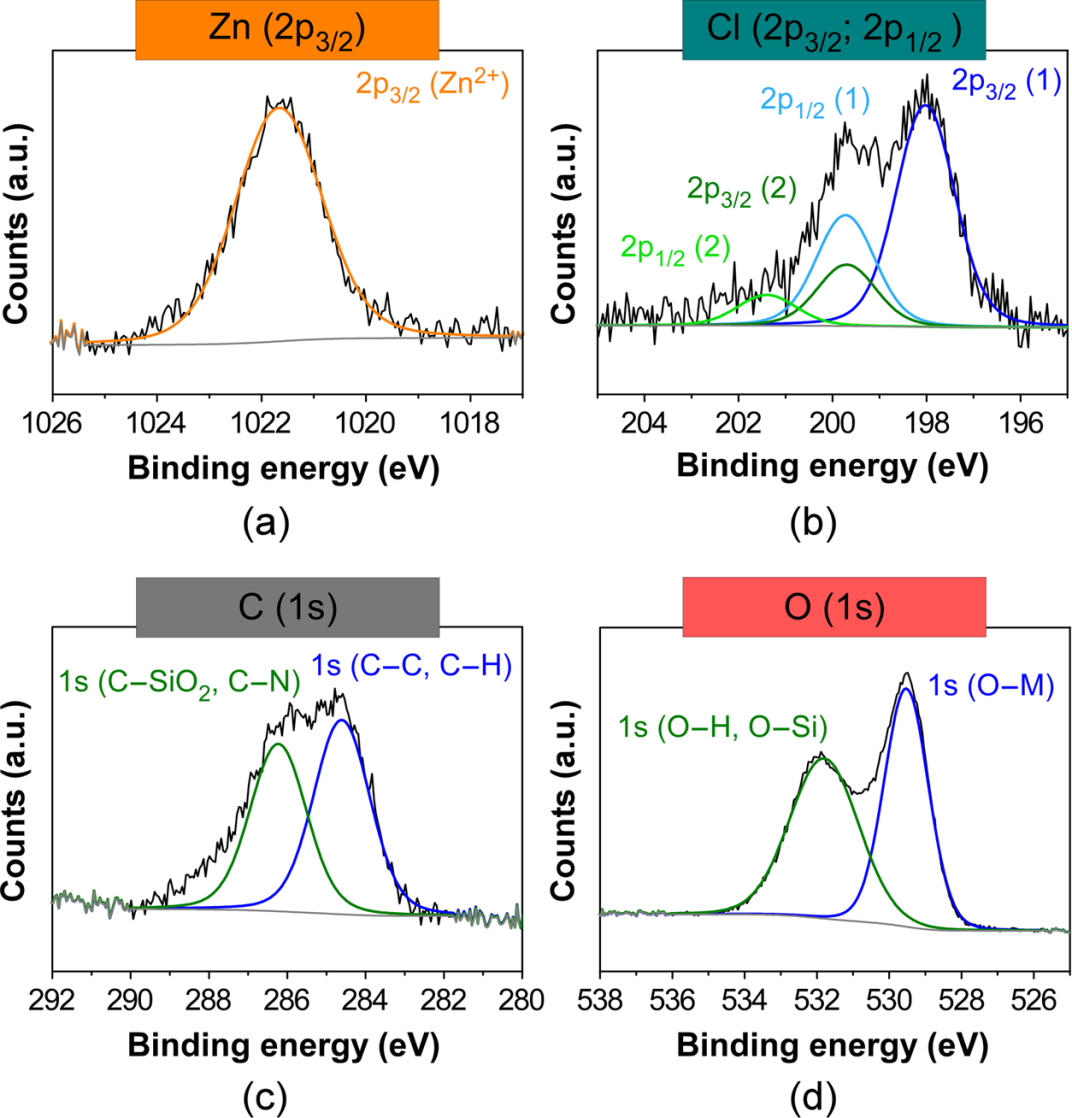
XPS data fitting for (a) Zn (2p_2/3_), (b) Cl (2p), (c) C (1s), and (d) O (1s) lines in **2**. Gray line for background end envelope omitted for clarity in all spectra.

The FT-IR spectrum of **2** (Figure S16, Supporting Information) exhibits absorption bands at 552–580 cm^–1^ related to the magnetite core (Fe–O stretching vibration), peaks between 1224 and 890 cm^–1^ associated with the SiO_2_ shell (Si–O and Si–O–Si), and absorption bands corresponding to the organic moiety of the IL at 1627 and 1566 cm^–1^ (C=N/C=C and C–N/C–C).^57,58^ Interestingly, the STEM-XEDS elemental map over a group of nanoparticles showed the presence of Fe and Zn, Cl, Si, and O in the shell, which provides evidence of the silica coating and the Zn-containing IL on the Fe_3_O_4_ NP surface (Figure 2c). XEDS spectroscopy also confirmed the presence of Fe, Zn, Cl, and Si in the sample (Figure S17).

In addition, the chemical features of the Zn-containing ionic liquid anchored on the NP surface were assessed by XPS. The fitting of XPS data of fresh NPs shows a Zn/Cl ratio close to 1:1 (Table 1 and Figure 3a,b),^59,60^ which suggests that, during catalyst preparation, part of chloride (Cl^–^) ions has been exchanged by hydroxide (OH^–^) ligands to afford anionic [ZnCl­(OH)_2_(H_2_O)_n_]^−^ type complexes that assembly to the silica shell balancing the cationic charge of the imidazolium groups (+1). Note that, in the preparation of **2**, water was employed as a solvent, which has been described to lead to a partial displacement of Cl^–^ ligands by OH^–^ in the coordination sphere of Zn­(II).^61–63^ Consider also that the formation of zinc hydroxide (β_1_: 3.16 × 10^7^; β_2_: 2.51 × 10^16^; β_3_: 1.58 × 10^28^ for [Zn­(OH)*_n_*]^2–*n*^; β = hydrochemical equilibrium constant) and zinc hydroxichloride (β: 3.02 × 10^7^ for [ZnCl­(OH)]) complexes is favored by their relatively high stability constants with respect to simple zinc chloride complexes (β_1_: 2.57; β_2_: 2.82; β_3_: 3.16 for [ZnCl*_n_*]^2–*n*^).^64^ On the basis of this thorough characterization study, Fe_3_O_4_@SiO_2_@(mim)­[ZnCl­(OH)_2_] was proposed as the actual structure of the nanocatalyst **2**.

**Table 1:** Fitted XPS Lines and Elemental Quantitative Analysis Normalized for Zn and Cl for **2** ^a^

element	line	*E*_b_ (eV)	at. (%)	at. (rel.)
Zn	Zn (2p_3/2_)	1021.7	53.4	1.00
Cl	Cl (2p_3/2_) Cl (2p_1/2_)	198.0/199.7 199.7/201.4	46.6	0.87

a XPS accuracy for elemental analysis is usually 5–15%.^65^

Finally, according to EA and ICP, the IL content on the NP surface is *ca*. 6.12 and 7.30 wt %, respectively. These results match with TGA data (*ca*. 7.09 wt %, see Figure S18, Supporting Information). The thermal stability of **2** and its durability at catalytic temperatures were also studied by TGA, revealing a decomposition temperature around 230 °C (Figure S18, Supporting Information)­.

**Figure 4:**
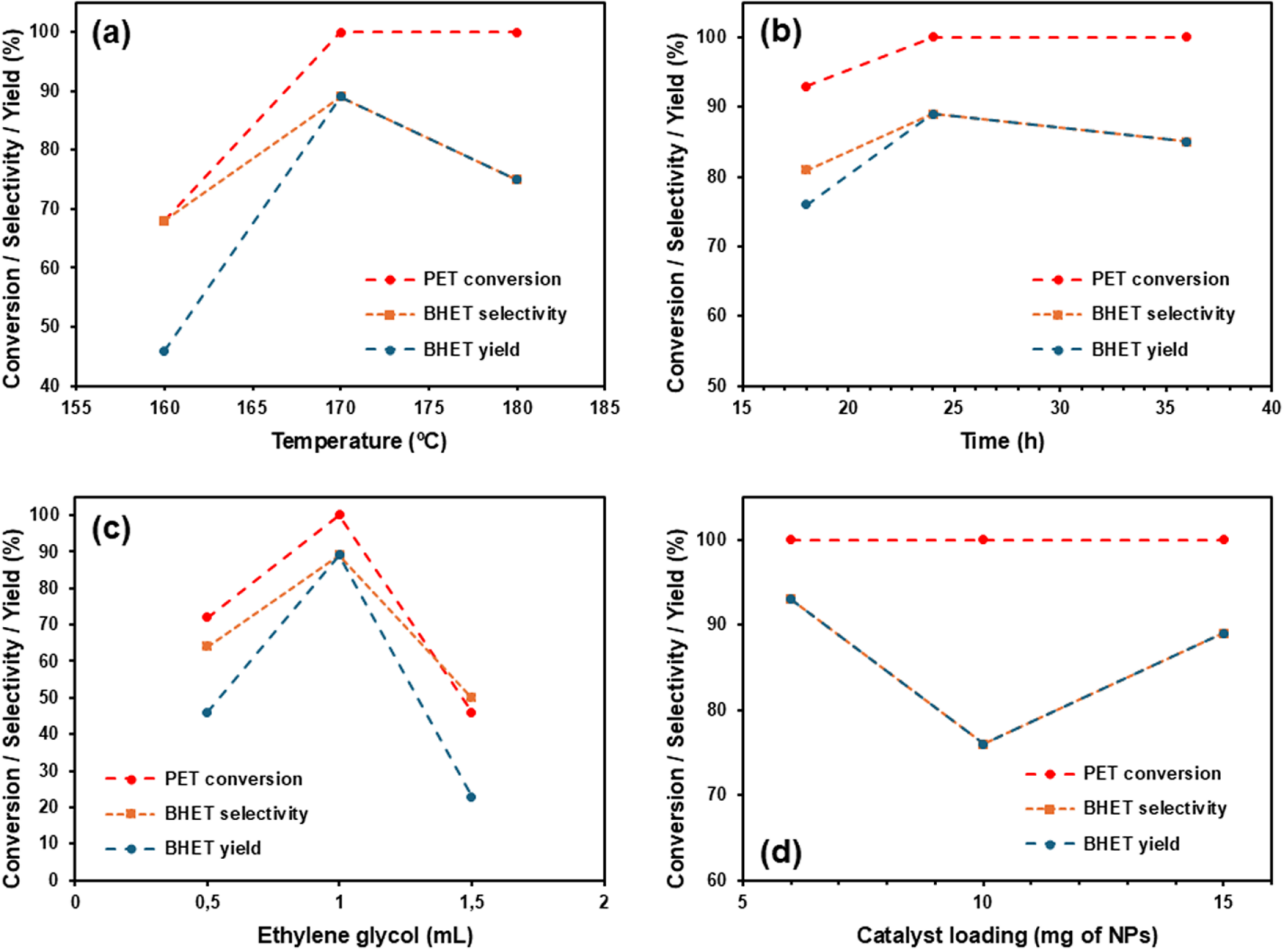
(a) Influence of temperature on the glycolysis of PET catalyzed by **2**. Reagents and conditions: PET (100 mg), **2** (15 mg), EG (1 mL), 24 h. (b) Influence of time on the glycolysis of PET catalyzed by **2**. Reagents and conditions: PET (100 mg), **2** (15 mg), EG (1 mL), 180 °C (170 °C inside the reaction flask). (c) Influence of EG amount in the glycolysis of PET catalyzed by **2**. Reagents and conditions: PET (100 mg), **2** (15 mg), 180 °C (170 °C inside the reaction flask), 24 h. (d) Influence of catalyst loading in the glycolysis of PET catalyzed by **2**. Reagents and conditions: PET (100 mg), EG (1 mL), 180 °C (170 °C inside the reaction flask), 24 h.

### Glycolysis of PET, PBT, and BPA-PC Catalyzed by 2

In order to investigate the catalytic performance of **2** for the glycolysis of polyesters in EG, the nanocatalyst was first evaluated in the depolymerization of postconsumer PET (Scheme 1a) under various reaction conditions (Table 2)­. Our previous study using Fe_3_O_4_@SiO_2_@(mim)­[FeCl_4_] (**1**) as catalytic system gave rise to a yield and selectivity >99% for the glycolysis of PET into BHET in EG after 24 h at 180 °C and with a 15 wt % catalyst loading. Thus, we initially employed similar conditions for **2** (Zn-containing IL loading = 0.97 mol %) to those used with **1** (Table 2, entry 1; conversion >99%, selectivity = 75%, yield = 75%). Importantly, a decrease in the reaction temperature from 180 to 170 °C provided better catalytic results in terms of both yield (89%) and selectivity of BHET (89%) (Table 2, entry 2). The lower BHET selectivity and thus the decrease in BHET yield as the reaction temperature increases can be related to the equilibrium established between monomers and oligomers.^44,47,66^ Therefore, there may be a repolymerization of BHET into oligomers at higher temperatures. On the other hand, a further reduction of 10 °C in the temperature (from 170 to 160 °C) led to much lower PET conversion (68%) and BHET yield (46%) (Table 2, entry 3). Therefore, 170 °C was selected as the optimal reaction temperature (Figure 4a). We then varied the reaction time (Figure 4b), which revealed that 24 h seems to be the optimal choice (Table 2, entry 2), since shorter reaction times gave lower conversion and yield (Table 2, entry 4; conversion = 93%, yield = 76%), and longer times did not improve the BHET selectivity and yield (Table 2, entry 5; selectivity = 85%, yield = 85%). In addition, the amount of EG in the reaction media had a significant effect on both conversion and selectivity (see Figure 4c and Table 2, entry 2 vs 6 and 7), 1 mL being the optimal EG volume for this process. This amount corresponds to an EG:PET ratio of 9:1 (w/w), which can be considered somewhat high.^28,31,37,39,41,44,47–49,66^ Finally, the catalyst loading was investigated (Figure 4d), which could be reduced to 6 wt % (Zn-containing IL loading = 0.39 mol %) keeping the quantitative conversion and the high selectivity and yield (Table 2, entry 9; selectivity = 93%, yield = 93%). In fact, it can be observed a slight decrease in yield and selectivity at higher catalyst loadings. In this sense, Zhang et al. suggested that an increase of the catalyst loading in PET degradation by metal-containing ILs may lead to a rapid initial production and subsequent accumulation of BHET, which can then repolymerize as the reaction evolves.^47^

**Scheme 1:**
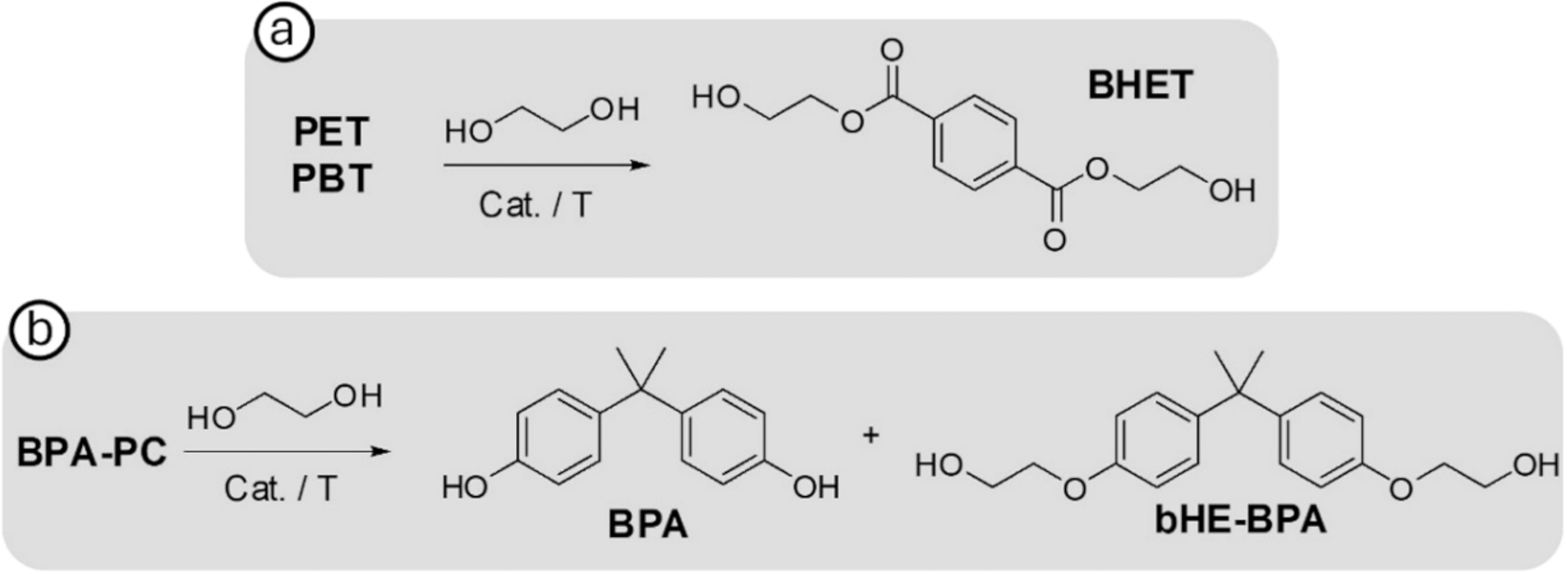
(a) Glycolysis of PET and PBT. (b) Glycolysis of BPA-PC

**Table 2:** Optimization Parameters for the Glycolysis of PET Catalyzed by **2** ^a^

entry	[cat] (mg)	[IL]^b^ (mol %)	EG (mL)	time (h)	*T* (°C)	conversion (%)^c^	selectivity (%)^d^	yield (%)^e^
1	15	0.97	1	24	180	>99	75	75
2	15	0.97	1	24	170	>99	89	89
3	15	0.97	1	24	160	68	68	46
4	15	0.97	1	18	170	93	81	76
5	15	0.97	1	36	170	>99	85	85
6	15	0.97	0.5	24	170	72	64	46
7	15	0.97	1.5	24	170	46	50	23
8	10	0.65	1	24	170	>99	87	87
**9**	**6**	**0**.**39**	**1**	**24**	**170**	>**99**	**93**	**93**
10^d^	6	0.39	1	24	170	>99	>99	>99
11^e^	6	0.39	1	24	170	>99	80 (9)	80 (9)

a Reagents: PET (100 mg). Product: BHET.

b Zn-containing IL loading.

c Conversion = (*W*_0_ – *W*_1_)/*W*_0_, where *W*_0_ is the initial weight of polyester and *W*_1_ is the weight of undepolymerized polyester.

d Product determined through ^1^H NMR spectroscopy by the use of 1-phenyl-1,2-ethanediol as internal standard (average of two runs).

e Reagents: PBT (100 mg). Product: BHET. ^*e*^ Reagents: BPA-PC (100 mg). Product: BPA (pHE-BPA).

With the optimal reaction conditions in hand, we explored both the versatility of this catalytic system for the depolymerization of other polyesters (PBT and BPA-PC) and its potential as a magnetically recoverable catalyst. As previously mentioned, the glycolysis of PET under the optimized conditions gave quantitative conversion, and 93% selectivity and yield toward BHET (Table 2, entry 9). Pleasantly, when PBT was depolymerized, BHET yield >99% was obtained (Table 2, entry 10 and Scheme 1a). We also investigated the glycolysis of BPA-PC pellets (Table 2, entry 11). Quantitative conversion, high selectivity toward bisphenol A (BPA) monomer (80%), and a small quantity (9%) of bisphenol A bis­(2-hydroxyethyl)­ether (bHE-BPA) was observed (Scheme 1b). Note that all products, BHET and BPA, were isolated with high purity (for further details see Materials and Methods section), which was confirmed using a variety of techniques, such as ^1^H and ^13^C NMR, EA, FT-IR, TGA and DSC (see the Materials and Methods section and Figures S19–S33, Supporting Information).

The selectivity and yield observed in the polyester depolymerization process catalyzed by **2** follow the decreasing trend PBT *>* PET *>* BPA-PC. This tendency agrees with the mechanism proposed for the glycolysis of polyesters mediated by metal-containing ILs like the zinc-based catalyst anchored on the surface of **2**. To carry out the polymer degradation, the metal ion in the IL interacts with EG, and the imidazolium cation interacts with the ester groups of polyesters through hydrogen-bonding (for more details about the mechanism, see ref 43). Therefore, it may be expected that the highest yield is observed in the glycolysis of the least sterically hindered polymer around the ester group where substrate activation occurs, that is, PBT. On the other hand, the least selective process is observed in the depolymerization of BPA-PC, the most sterically hindered polyester in which two aromatic rings are in close proximity to the ester group.

The nanocatalyst **2** was easily recovered through magnetic separation with an external Nd_2_Fe_14_B permanent magnet in only 30 s (Figure 5 and Video S1). This simple recycling method allowed us to reuse **2** for 12 cycles without significant decrease in the catalytic activity. Figure 6 shows the excellent recycling performance of **2** for the depolymerization of PET, PBT, and BPA-PC. It is worth noting that **2** was efficiently reused for up to 20 consecutive cycles in the glycolysis of BPA-PC, which demonstrates the exceptional recycling behavior of the nanocatalyst described herein! (Figure 7).

**Figure 5:**
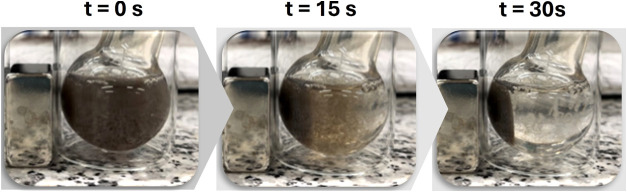
Magnetic separation of **2** using an external magnet.

**Figure 6:**
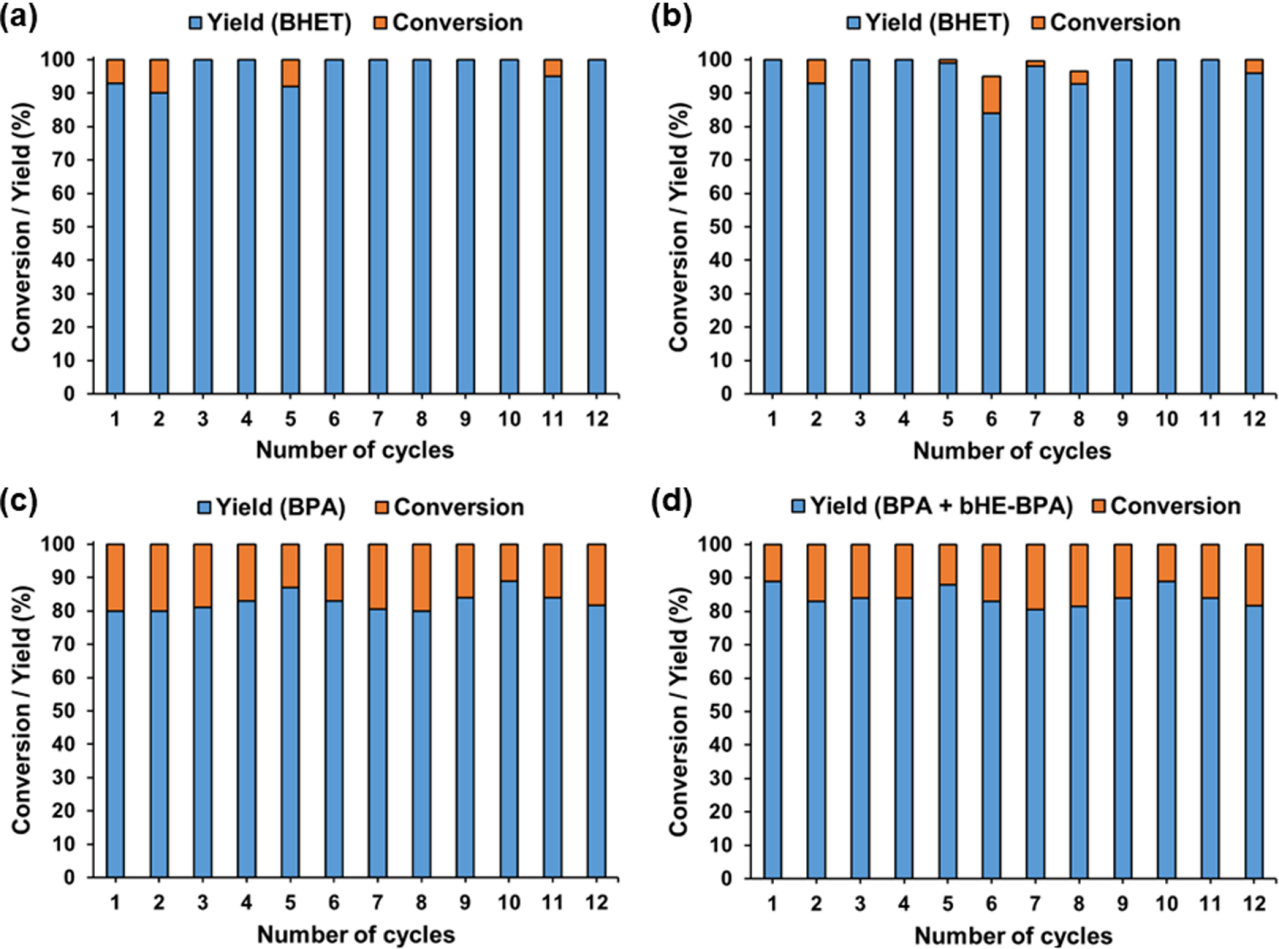
Reuse of **2** in the glycolysis of (a) PET, (b) PBT, (c, d) BPA-PC. Reagents and conditions: **2** (6 mg), polyester (100 mg), EG (1 mL), 180 °C (170 °C inside the reaction flask), 24 h.

**Figure 7:**
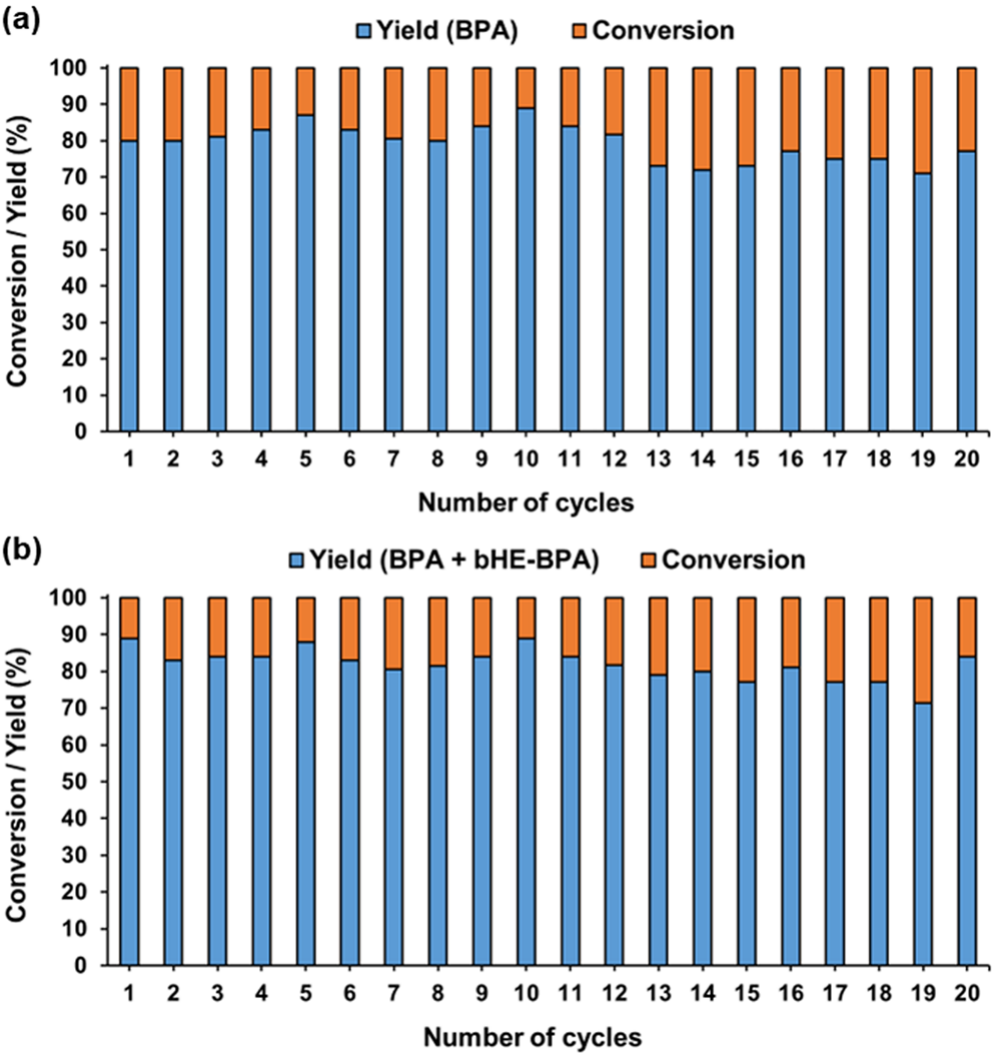
Recyclability of **2** in the glycolysis of BPA-PC. a) Yield of BPA. b) Yield of BPA + bHE-BPA. Reagents and conditions: **2** (6 mg), BPA-PC (100 mg), EG (1 mL), 180 °C (170 °C inside the reaction flask), 24 h.

In addition, gram-scale reactions were performed with 2 g of commercial PET sourced from water bottles obtained from a local market, and PBT and BPA-PC pellets (Table 3 and Section 9, Supporting Information). Remarkably, quantitative consumption of PET and *ca*. 80% isolated yield toward BHET were observed in the first and second catalytic cycles. Similarly, nearly complete conversion of PBT and more than 80% isolated yield toward BHET were obtained in the first and second runs. In addition, quantitative conversion of BPA-PC was noted, while the isolated yields toward BPA were 59.8% and 61.8% in the first and second catalytic cycles, respectively. These experiments suggest that **2** could be applied at a larger scale in the chemical recycling of polyester plastics.

**Table 3:** Gram-Scale Glycolysis of PET, PBT, and BPA-PC Catalyzed by **2**.^a^

catalytic cycle	conversion (%)^b^	isolated yield (%)
	*PET*	
1	>99	79.4
2	>99	79.9
	*PBT*	
1	98.1	84.8
2	>99	83.0
	*BPA-PC*	
1	>99	59.8
2	>99	61.8

a Reagents: **2** (120 mg), polyester (2 g), EG (20 mL), 24 h, 180 °C (170 °C inside the reaction flask).

b Conversion = (*W*_0_ – *W*_1_)/*W*_0_, where *W*_0_ is the initial weight of polyester and *W*_1_ is the weight of undepolymerized polyester.

A series of control experiments were also carried out to confirm the importance of the metal-containing IL anchored on the NP surface for the polyester degradation process. In this way, no reaction was observed in the absence of the catalyst. Furthermore, Fe_3_O_4_ ^52^ (Figures S41–S42, Supporting Information) and Fe_3_O_4_@SiO_2_ ^53^ (Figures S43–S44, Supporting Information) NPs lead to lower yields (71 and 78% BHET, respectively) than Fe_3_O_4_@SiO_2_@(mim)­[ZnCl­(OH)_2_]. Fe_3_O_4_@SiO_2_@(mim)­Cl^54^ (Figures S45–S46, Supporting Information) and Fe_3_O_4_@SiO_2_@(mim)­[PF_6_][Bibr ref53] ((mim)­[PF_6_]: methylimidazolium-hexafluorophosphate; Figures S47–S48, Supporting Information) NPs were also tested as catalysts, showing less activity (*ca*. 85% BHET yield) than the hybrid material described herein (>99% BHET yield in 8 out of 12 reaction cycles).

Finally, the catalyst described in this work was compared with similar catalytic systems reported in the literature (Table S1, Supporting Information). This catalyst required a higher reaction time (24 h) to perform the depolymerization of PET than those needed on average with purely organic and metal-containing ionic liquids, magnetic NPs, and ionic liquids immobilized on magnetic supports (1–4 h). However, we must take into account that the BHET yield achieved is very high (>99%) and the temperature (170 °C) is lower than those used with analogous catalysts consisting of ionic liquids immobilized on magnetic supports, which are in the 190–200 °C range. Indeed, the reaction temperature is within the range typically applied with ionic liquids as catalyst and much lower than those described for magnetic nanoparticles (180–300 °C). In addition, assuming that the catalytically active species is the Zn-containing ionic liquid supported on the NP surface, its loading is as low as 0.39 mol % (1:227 w/w) relative to the moles of PET units when 6 wt % of nanoparticles are employed. Finally, the catalytic systems shown in Table S1 exhibit variable recyclability, from 1 to 10 runs, while the one developed herein was recovered and reused for at least 12 consecutive reaction cycles without appreciable loss of activity. In fact, **2** was successfully recycled over 20 runs, which highlights the extraordinary robustness and recyclability of this catalyst.

### Characterization of the Recovered Catalyst

After the polyester glycolysis process, the nanocatalyst **2** was retrieved from the reaction phase and studied through different characterization techniques (XRPD, HR-TEM, STEM, XEDS, IR, TGA, XPS) in order to verify the preservation of the structure of the catalytically active species. No significant differences were detected when comparing the XRPD diffractograms of the nanocatalyst before and after the recycling experiments, in which the most notable modification is an increased background at low angles due to the amorphous contribution of the polymer (Figure S49, Supporting Information). Schrerrer analysis yields a particle size of 9.1 ± 0.2 Å for the recovered catalyst, which is very similar within the error to that observed for fresh nanoparticles (9.0 ± 0.1 Å) from XRPD data collected with similar statistics. The recovered catalyst was also analyzed by HR-TEM, which showed the same size (10 ± 4 nm) and morphology as the nonused NPs while keeping the amorphous layer of SiO_2_ (Figures S50–S52, Supporting Information). Nevertheless, Cl was not observed in the STEM-XEDS elemental map (Figures S53–S54, Supporting Information), evidencing a clear loss of Cl^–^ ions of the zincate-based IL anchored on the NP surface, likely caused by a ligand exchange process in the coordination sphere of Zn between Cl^–^ and OH^–^ ions or even due to the coordination of glycolate species (deprotonated EG) to the metal center.

To gain more insight into this phenomenon and given that the NP surface adsorbs organic material (monomers and oligomers) from the degradation process that may cause interferences (see Figures S57 and S59 in Supporting Information corresponding to TGA and IR analyses, respectively), the fresh catalyst was exposed to the reaction conditions in the absence of polyester and washed with dichloromethane instead of water to avoid an exchange of potential Zn-coordinated glycolate species by OH^–^ ions. The recovered catalyst was then investigated through TGA and XPS. Along this line, despite at the initial stage the NP surface is comprised by zinc­(II) hydroxichloride complexes, the XPS analysis of the recovered catalyst does not display any trace of chloride accompanying the Zn­(II) atoms (Table 4, Figure 8a,b, and Figures S55a and S55b, Supporting Information). This suggests that during the glycolysis process, the high reaction temperatures can promote further ligand exchange, leading to the formation of zinc-glycolate type complexes and the release of H_2_O and volatile HCl (Figure 9). In fact, the formation of Zn­(II) glycolate complexes has been previously reported under similar reaction conditions (glycol solvent at 150–198 °C).^67^ Therein the reaction of zinc­(II) formate dihydrate with EG forms zinc­(II) glycolates ([Zn_2_(HCOO)_2_(C_2_H_4_O_2_)] and [Zn­(C_2_H_4_O_2_)]) and releases formic acid and H_2_O. As shown by the X-ray structure of the latter compound, the glycolate adopts a κ:*O*,*O′* coordination mode to set a stable five-membered chelate ring with a Zn­(II) atom. Despite this change in the coordination sphere generating an almost negligible shift of the Zn 2p_3/2_ line (ΔE_B_ = +0.02 eV), a new peak is observed in the energy range of the C 1s line in the spectra of the recovered catalyst (Figure 8c and Figure S55c, Supporting Information). Such a line is related to glycolate C atoms^68^ and sited at higher binding energies due to the deshielding produced by electronegative O atoms (C–O bond). The XPS data fitting reveals a stoichiometric Zn:C_(1s(C–O))_ ratio close to 1:2 (see Table 4), which is consistent with one glycolate species per zinc unit. The O 1s line attributable to the glycolate moiety appears overlaid in a bunch together with those from both OH^–^ ligands and oxide anions of SiO_2_, but in the quantitative analysis of the high-resolution spectra, a new O 1s component emerges in the deconvolution at 532.2 eV. This quantification also shows a stoichiometric ratio Zn:O_(1s(O–C))_ of 1:2 (Table 4, Figure 8d, and Figure S55d, Supporting Information), and is again consistent with a glycolate per zinc unit. When the catalyst recovered after the polyester glycolysis process was washed with H_2_O in the workup, the Zn 2p_2/3_ signal was kept, but the C 1s peak associated with the glycolate was meaningfully reduced (Figure S56, Supporting Information), probably due to hydrolysis of the Zn–glycolate complex. In any case, re-exposure to the catalytic reaction conditions would allow the regeneration of the Zn–glycolate adduct. All in all, the formation of such zinc­(II) glycolate complexes can play a key role in the depolymerization process by enabling the formation and subsequent transfer of glycolate species to afford BHET.

**Figure 8:**
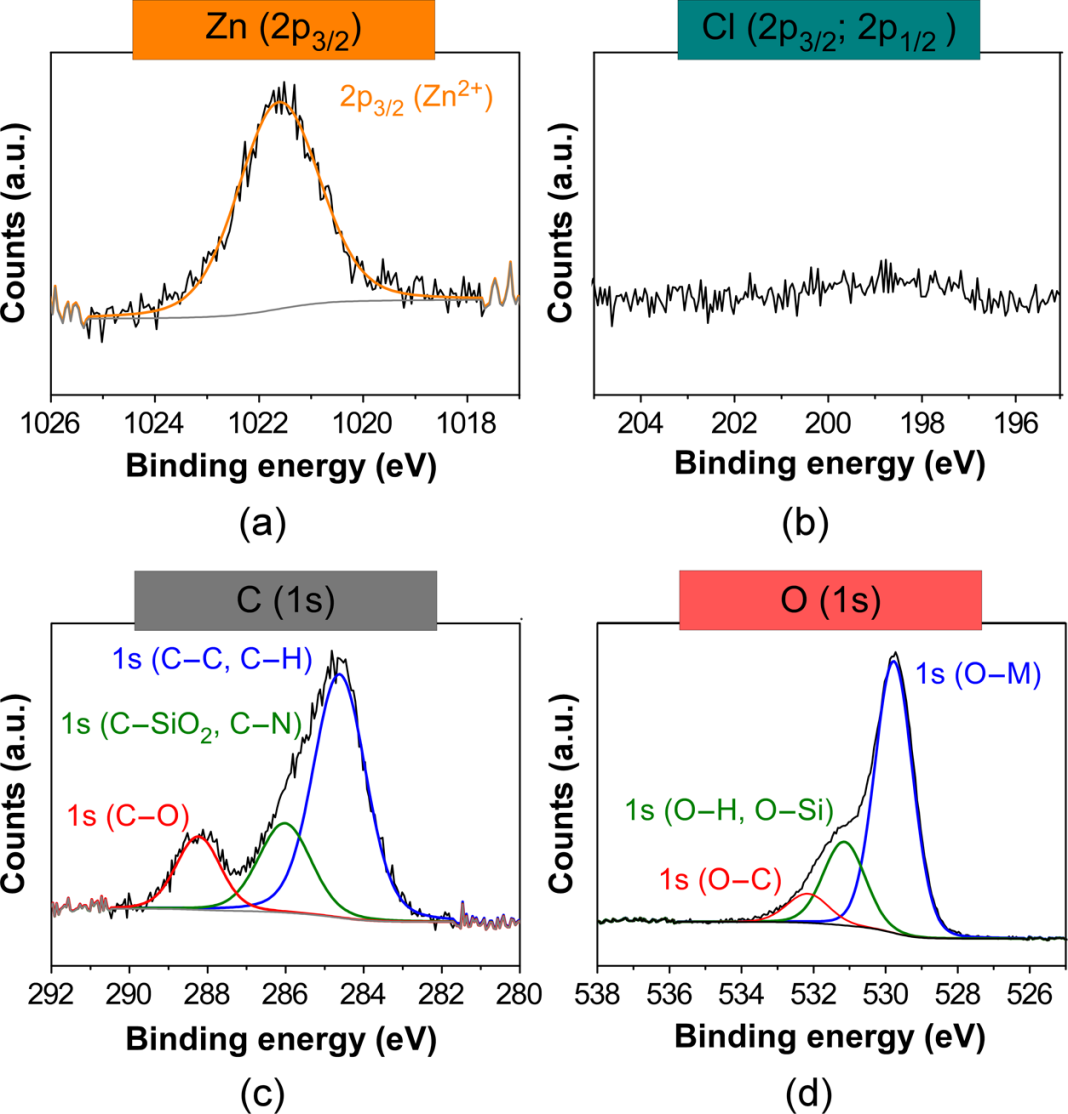
XPS data fitting for (a) Zn (2p_2/3_), (b) Cl (2p), (c) C (1s), and (d) O (1s) lines in the recovered catalyst after heating at 180 °C in the presence of EG and washing with CH_2_Cl_2_. Gray line for background end envelope omitted for clarity in all spectra.

**Figure 9:**
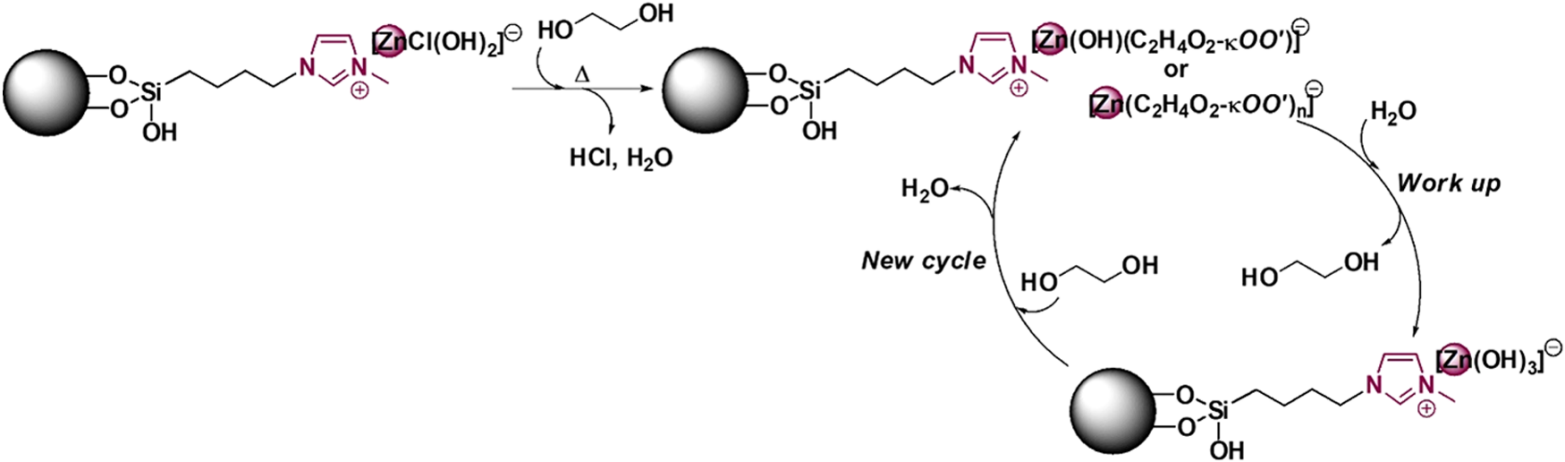
Proposed scheme of the ligand exchange in the Zn-based catalyst during the glycolysis process at a high temperature.

**Table 4:** Fitted XPS Lines and Elemental Quantitative Analysis Normalized for Zn and Cl for the Recovered Catalyst after Heating at 180 °C in the Presence of EG and Washing with CH_2_Cl_2_ ^a^

element	line	*E*_b_ (eV)	at. (%)	at. (rel.)
Zn	Zn (2p_3/2_)	1021.6	19.9	1.00
Cl	Cl (2p_3/2_)		0.0	0.00
	Cl (2p_1/2_)			
C	1s (C–O)	288.2	37.8	1.90
O	1s (O–C)	532.2	42.3	2.12

a XPS accuracy for elemental analysis is usually 5–15%.^65^

Accordingly, TGA analysis of the catalyst exposed to the reaction conditions in the absence of polyester displays a 10.62% mass loss, larger than that shown by the fresh catalyst (7.09%), which may be attributed to the higher weight of the glycolate ligand against Cl^–^ and OH^–^ ligands (Figure S58, Supporting Information). To strengthen the evidence of this Cl^–^ and OH^–^ exchange by glycolate, the Zn-containing ionic liquid (bmim)­[ZnCl­(OH)_2_] (bmim = 1-butyl-3-methylimidazolium) was prepared and exposed to the reaction conditions in the absence of polymer (for further details see Section 13 and Figures S60–S66, Supporting Information), *i*.*e*., this newly synthesized Zn-based IL was mixed with excess EG and heated at 170 °C. The exchange process was monitored by ^1^H NMR, which revealed that after 24 h at 170 °C, the substitution of Cl^–^ and OH^–^ ions by glycolate ligands occurs (Figures S61 and S65–S66, Supporting Information). Indeed, the ^1^H NMR spectra show a chemical shift for the −CH_2_– glycol peak to higher field, from 3.71 to 1.64 ppm, and the disappearance of the H signal corresponding to the –OH units. Such an analysis proves the formation of glycolate species and its coordination to the zinc­(II) center. Therefore, we can conclude that the formation of a Zn–ethylene glycolate complex takes place under the catalytic reaction conditions.

## Conclusions

In conclusion, a zinc-based ionic liquid has been immobilized on the SiO_2_ layer of silica-coated magnetite nanoparticles. The resulting Fe_3_O_4_@SiO_2_@(mim)­[ZnCl­(OH)_2_] nanoparticles have been extensively characterized and successfully used as a catalyst for the glycolysis of several polyesters. Such a nanocatalyst exhibits very high activity at 170 °C and, importantly, an excellent recycling behavior. Indeed, the catalyst was easily recovered with an external magnetic field, providing nearly 100% yield and selectivity in the depolymerization of PET and PBT into BHET for 12 consecutive reaction cycles. The robustness of the catalyst was demonstrated in the glycolysis of BPA-PC into BPA, since only a slight loss of activity was observed after 20 cycles of recovery and reuse, affording more than 80% BPA yield in most cases. What is more, depolymerization experiments with 2 g of PBT, BPA, or discarded commercial PET were successfully developed with quantitative consumption of polyester and high monomer yields during two catalytic cycles. Additionally, the structure of the catalyst remained practically intact after the catalytic experiments, since a profound characterization of the recovered catalytic system displayed only a loss of Cl^–^ ions from the Zn-containing IL as a result of a ligand exchange process with glycolate and OH^–^ species, which does not seem to affect the catalytic process and the reusability of the catalyst.

In summary, the nanocatalyst reported herein exhibits very high activity, robustness, and recyclability in the glycolysis of polyester plastics to obtain high-added-value products. Furthermore, the gram-scale experiments could open the door to broader applications of such a catalyst. Therefore, this type of catalytic system presents a huge potential for many new applications in sustainable chemistry, such as CO_2_ fixation into carbonates,^69,70^ biomass valorization,^71^ biodiesel production,^72^ and chemical recycling of other plastics (polyurethane, nylon, poly­(lactic acid), etc.) through different transformations (glycolysis, aminolysis, alcoholysis, etc.).^13,73^

## Supplementary Material





## Notes

Relevant data for the characterization of **2** (TEM, XRPD, FT-IR, XEDS, TGA, magnetic measurement, and N_2_ adsorption–desorption isotherm); spectra (NMR and IR) and thermal analyses (DSC and TGA) of isolated monomer samples, and synthesis and characterization of nonsupported ILs (NMR and IR) (PDF)
Recovery of nanocatalyst **2** through magnetic separation (AVI)
