# Supplementary material for: Developing a Highly Efficient and Magnetically Recoverable Nanocatalyst for Glycolytic Depolymerization of Various Polyesters
Source: ACS Sustain Chem Eng. 2025 May 23;13(21):7890–903. doi: 10.1021/acssuschemeng.5c01220 (PMC12139571; doi:10.1021/acssuschemeng.5c01220)
Supplement: Supplementary file 1 [file sc5c01220_si_001.pdf]

SUPPORTING INFORMATION

Developing a highly efficient and magnetically recoverable nanocatalyst for glycolytic depolymerization of various polyesters

Carmen Martín,*[,†] Maite Perfecto-Irigaray,[ψ,ǂ] Garikoitz Beobide,[ψ,¥] Elena Solana-Madruga,[†] David Ávila-Brande,[†] Marcos Laso-Quesada,[†] Imanol de Pedro,[§] Francisco A. Casado-Carmona,[‡,$] Rafael Lucena,[‡] Soledad Cardenas[‡] and Israel Cano*[,†]

[†] Departamento de Química Inorgánica, Universidad Complutense de Madrid, Madrid, 28040, Spain

[ψ] Departamento de Química Orgánica e Inorgánica, Universidad del País Vasco, UPV/EHU, Apartado 644, E-48080, Bilbao, Spain

[ǂ] ISIS Neutron and Muon Source, STFC Rutherford Appleton Laboratory, Didcot OX11 0QX, UK

[¥] BCMaterials, Basque Center for Materials, Applications and Nanostructures, UPV/EHU Science Park, E-48940 Leioa, Spain.

[§] CITIMAC, Facultad de Ciencias, Universidad de Cantabria, 39005 Santander, Spain

[‡] Affordable and Sustainable Sample Preparation (AS$_2$P) Research Group, Departamento de Química Analítica, Instituto Químico para la Energía y el Medioambiente (IQUEMA), Universidad de Córdoba, Campus de Rabanales, Edificio Marie Curie, 14071, Córdoba, Spain

[$] Department de Química, Facultat de Ciències, Universitat de les Illes Balears, Illes Balears, Carretera de Valldemossa Km 7.5, E-07122 Palma de Mallorca, Spain

Number of pages: 54

Number of Figures: 66

Number of Tables: 1

Contents

1. **HR-TEM images…..…………………………………………………………….……………..….. S3**
2. **XRPD analysis of Fe_3O_4@SiO_2@(mim)[ZnCl(OH)$_2$].. S9**
3. **Magnetic measurements of Fe_3O_4@SiO_2@(mim)[ZnCl(OH)$_2$].......………….................. S10**
4. **N_2 adsorption-desorption isotherm studies…………………………………………….. S11**
5. **FT-IR spectra of Fe_3O_4@SiO_2@(mim)[ZnCl(OH)$_2$]......………..……..…................... S13**
6. **XEDS spectrum of Fe_3O_4@SiO_2@(mim)[ZnCl(OH)$_2$]......…..............………………....... S14**
7. **TGA analysis of Fe_3O_4@SiO_2@(mim)[ZnCl(OH)$_2$]...............…..................................... S15**
8. **Characterization of BHET and bHE-BPA monomers…………………………………. S16**
9. **Gram-scale experiments………………………………………………………......... S27**
10. **TEM images of nanoparticles employed in the control experiments…………………. S33**
11. **Comparative analysis with similar catalytic systems reported in the literature…….... S37**
12. **Characterization of 2 after catalysis…………….. S38**
13. **Synthesis and characterization of (bmim)[ZnCl(OH)$_2$] and (bmim)[Zn((O)C_2H_4OH)$_3$]……….…………………………………………………….... S45**
14. **References………………….…………………………………………………....... S52**

1. TEM images

Figure S1. Low-magnification TEM image of Fe_3O_4@SiO_2@(mim)[ZnCl(OH)$_2$].

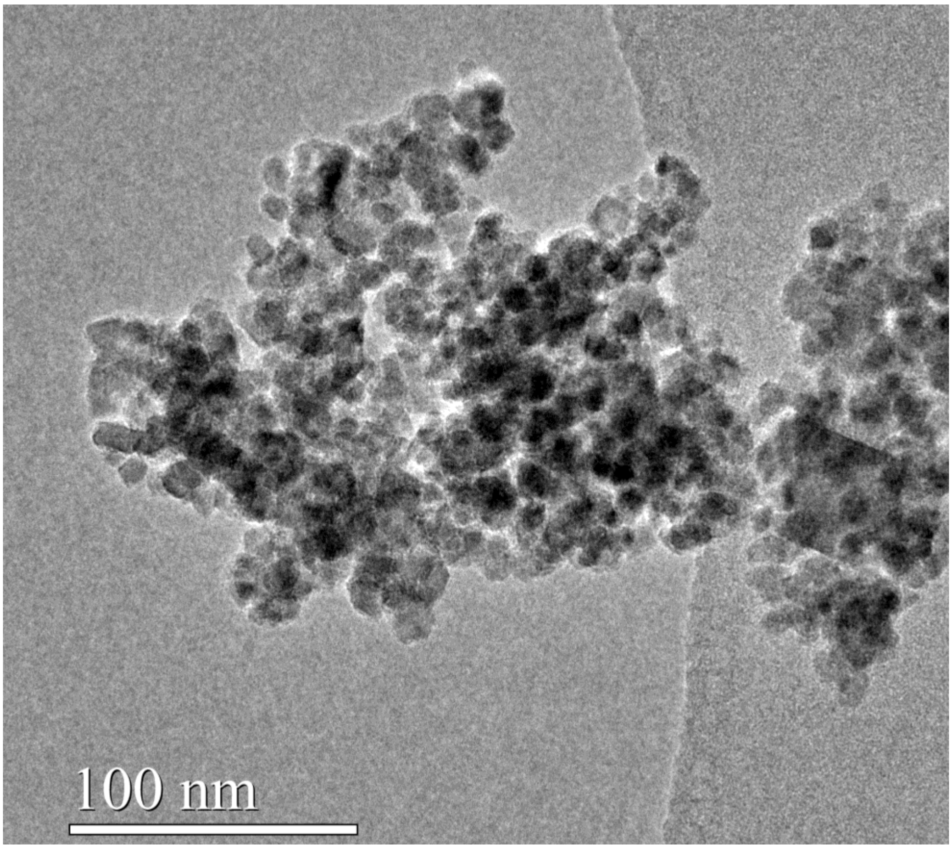

Figure S2. High-magnification TEM image of Fe_3O_4@SiO_2@(mim)[ZnCl(OH)$_2$].

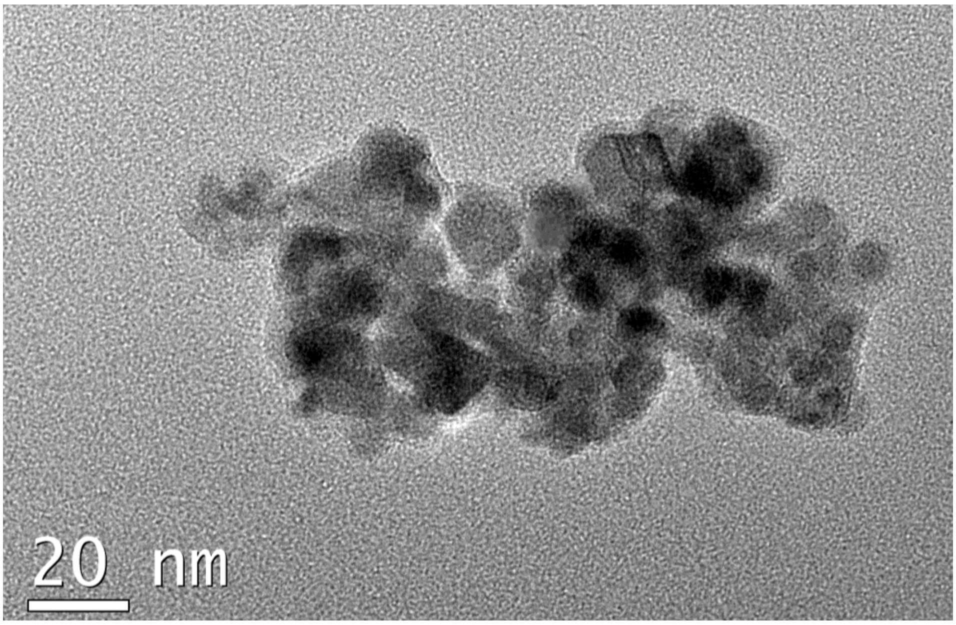

Figure S3. High-magnification TEM image of Fe_3O_4@SiO_2@(mim)[ZnCl$(OH)_2$].

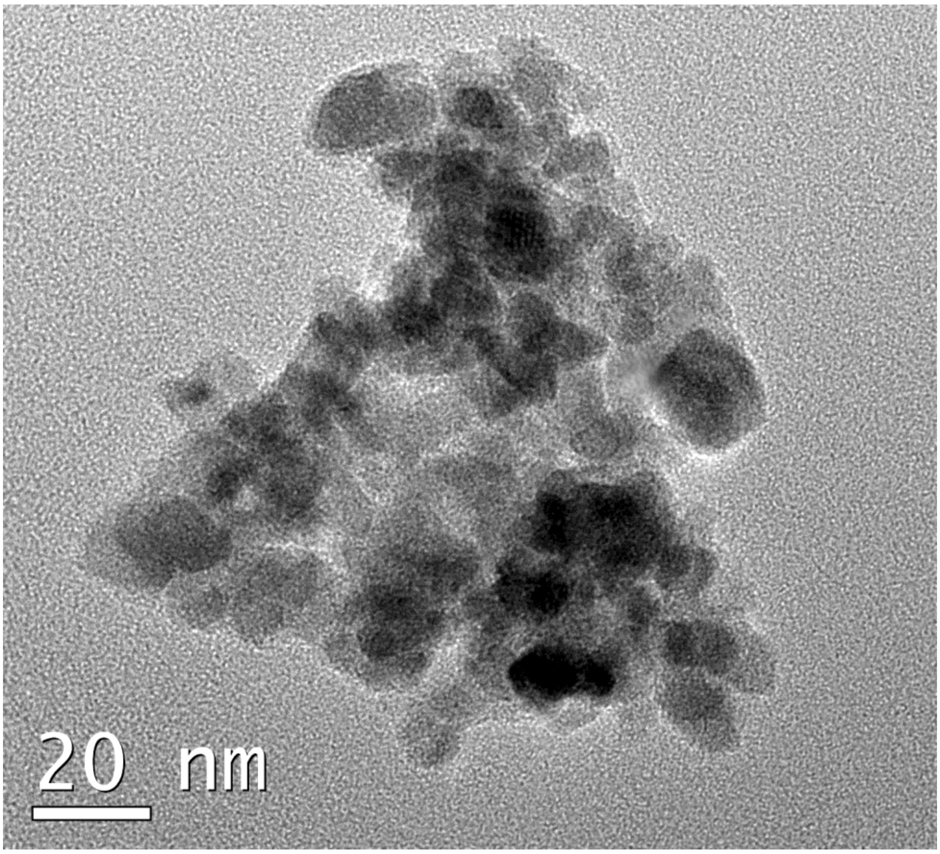

Figure S4. High-magnification TEM image of Fe_3O_4@SiO_2@(mim)[ZnCl$(OH)_2$].

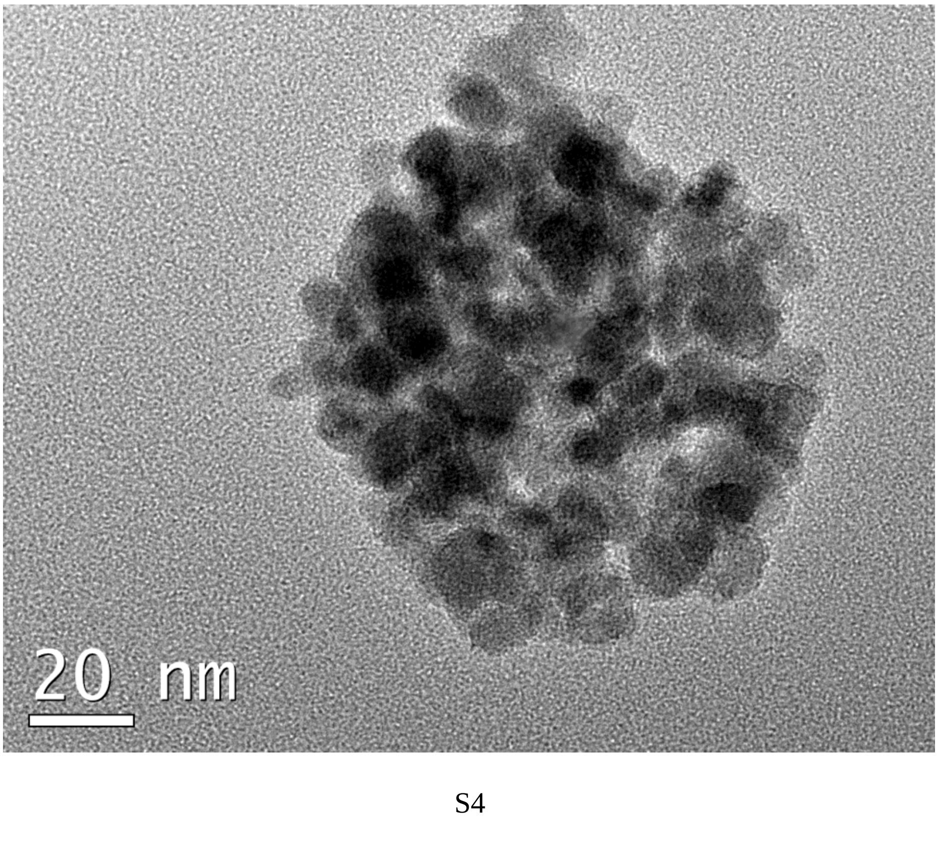

Figure S5. High-magnification TEM image of Fe_3O_4@SiO_2@(mim)[ZnCl(OH)$_2$].

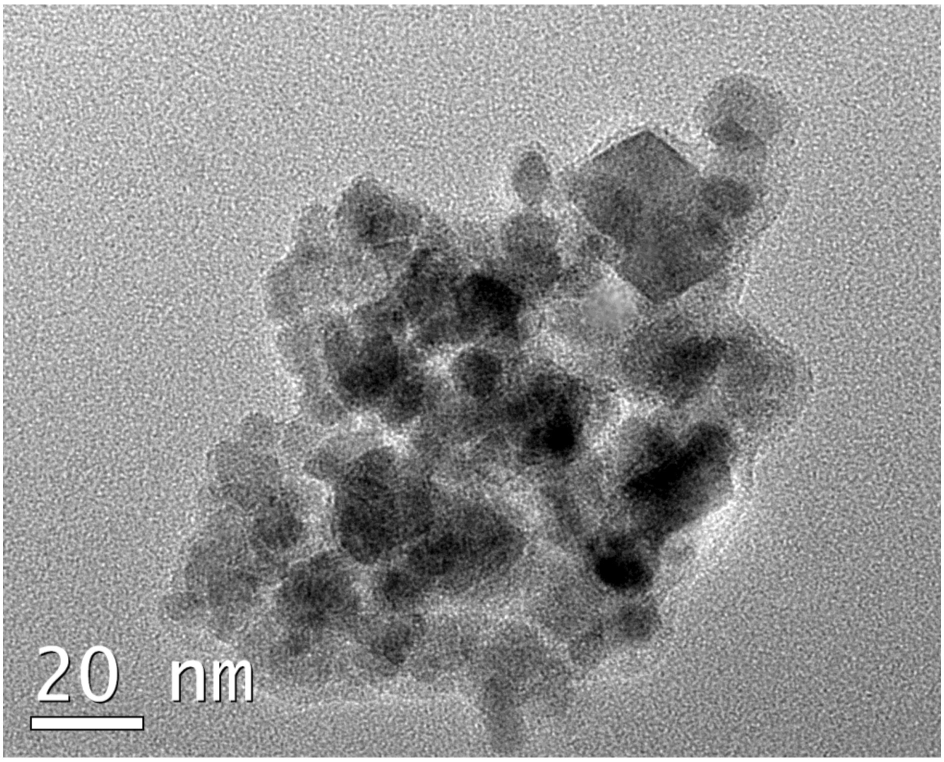

Figure S6. High-magnification TEM image of Fe_3O_4@SiO_2@(mim)[ZnCl(OH)$_2$].

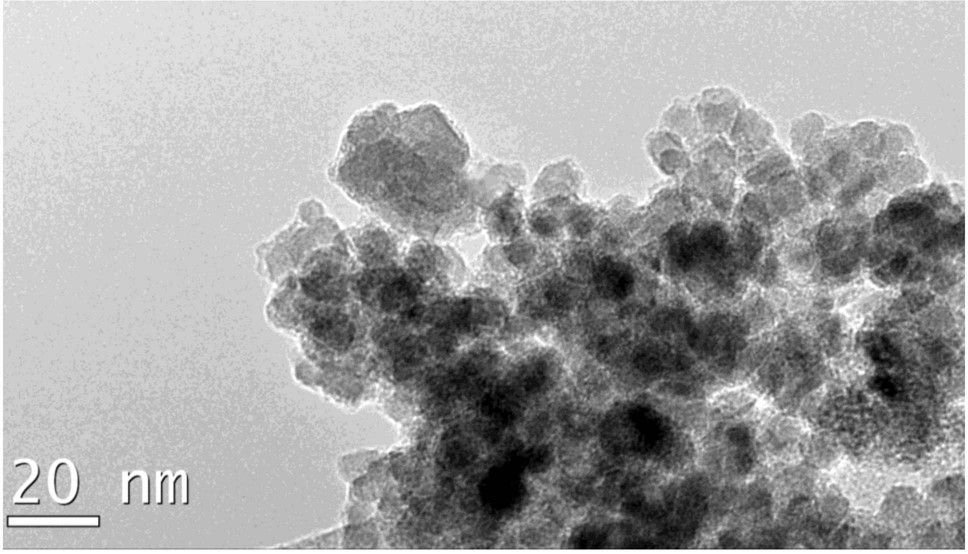

Figure S7. High-magnification TEM image of Fe_3O_4@SiO_2@(mim)[ZnCl(OH)$_2$].

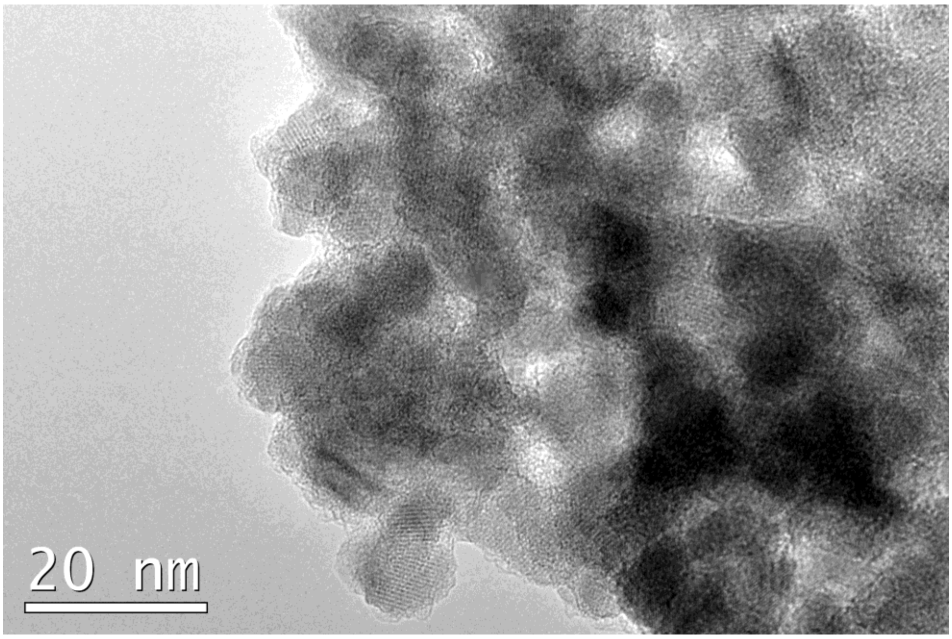

Figure S8. High-magnification TEM image of Fe_3O_4@SiO_2@(mim)[ZnCl(OH)$_2$].

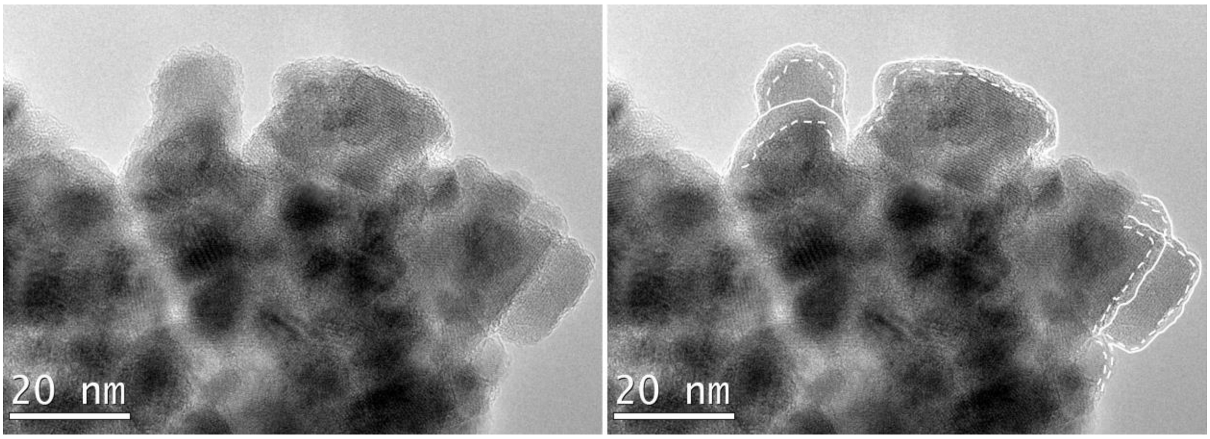

Figure S9. High-magnification TEM image of Fe_3O_4@SiO_2@(mim)[ZnCl(OH)$_2$].

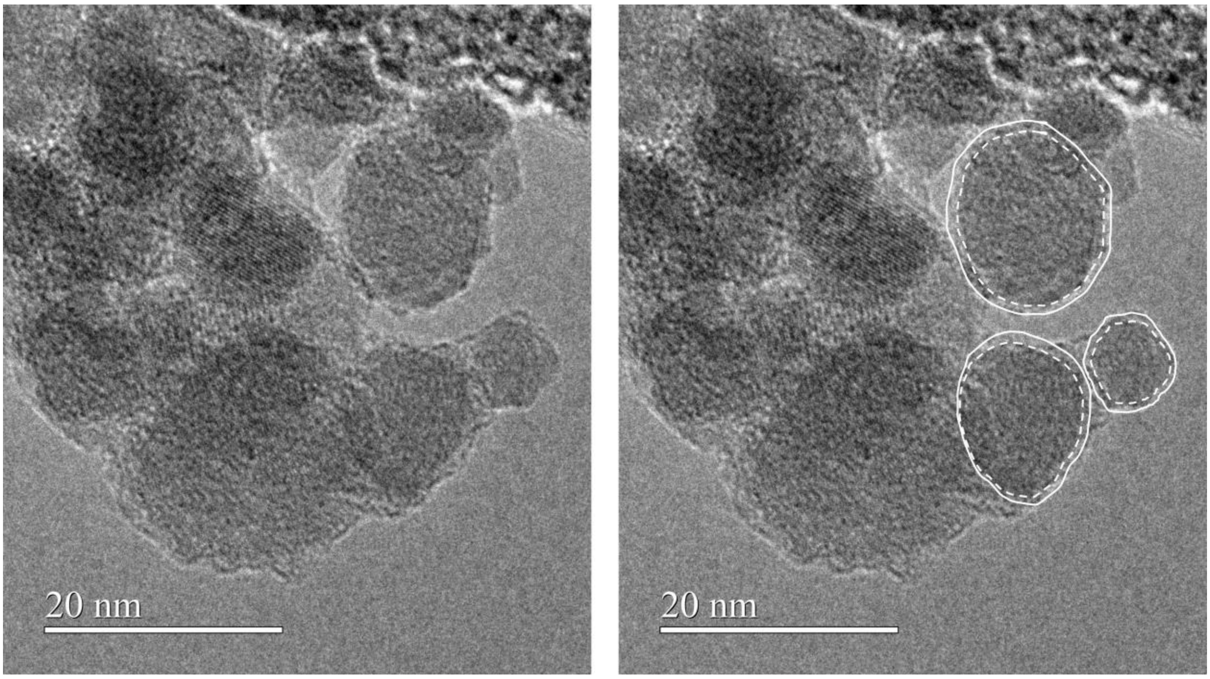

Figure S10. High-magnification TEM image of Fe_3O_4@SiO_2@(mim)[ZnCl(OH)$_2$].

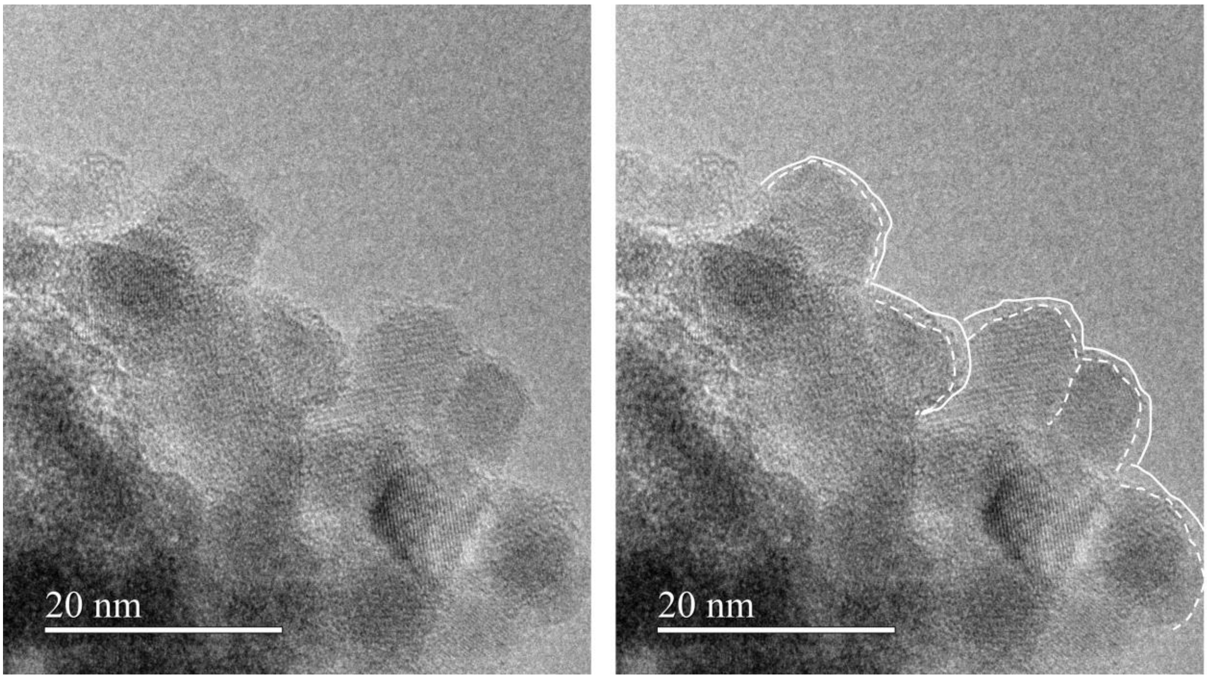

Figure S11. High-magnification TEM images of Fe_3O_4@SiO_2@(mim)[ZnCl(OH)$_2$] showing different thicknesses for the silica layer.

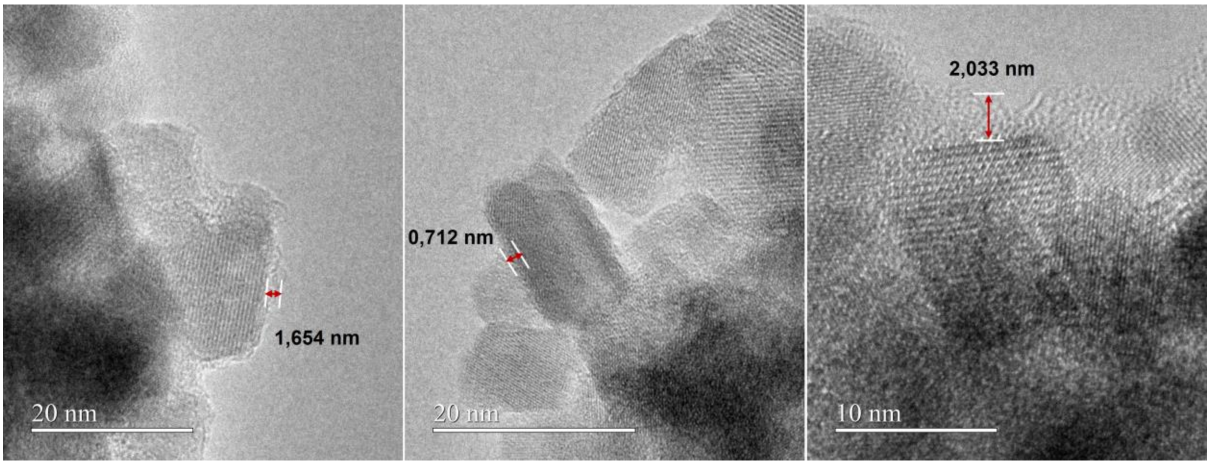

2. XRPD analysis of Fe_3O_4@SiO_2@(mim) [ZnCl(OH)$_2$]

Figure S12. Rietveld fit of the spinel structure of magnetite against XRPD data of Fe_3O_4@SiO_2@(mim)[ZnCl(OH)$_2$] (**2**) using the *Fd*-3*m* space group. The cell parameter refined to 8.348(3) Å. Observed (red points) powder diffraction pattern measured at room temperature and calculated (black solid line) pattern for the Rietveld fit. Position of the Bragg reflections are represented by vertical green bars. The observed-calculated difference pattern is depicted as a blue line at the bottom.

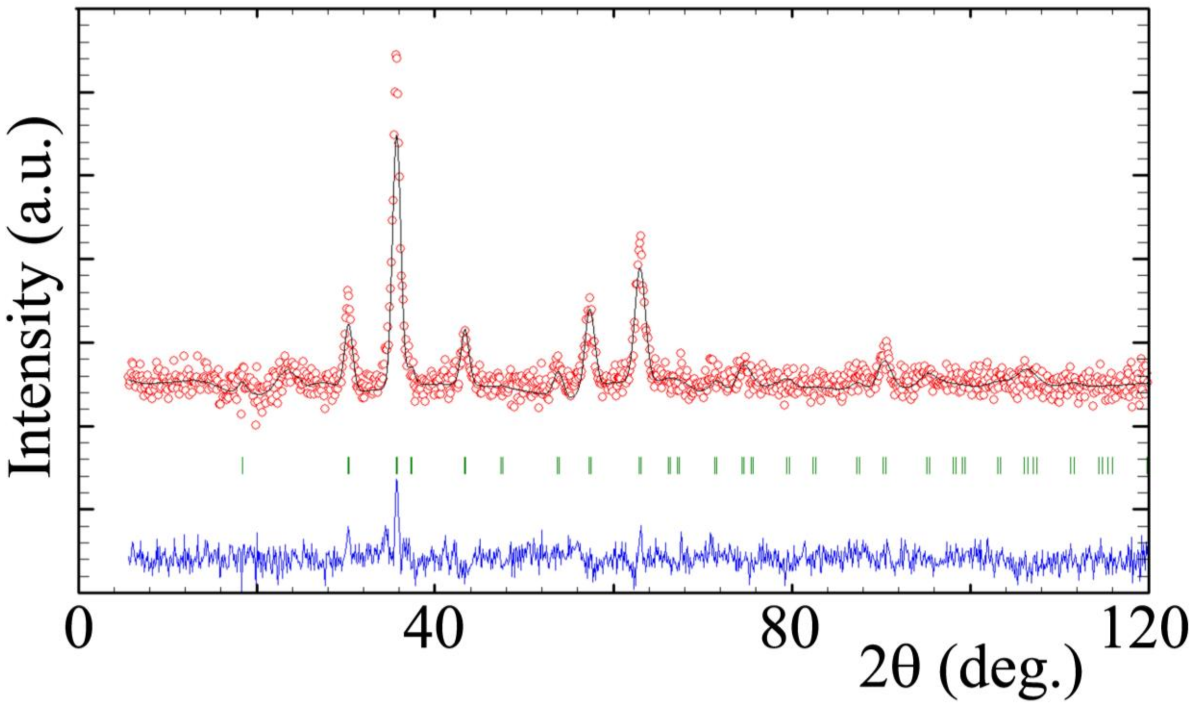

3. Magnetic measurements of $Fe_3O_4@SiO_2@(mim)[ZnCl(OH)_2]$

Figure S13. a) Thermal evolution of Field-Cooled (FC) and Zero-Field-Cooled (ZFC) magnetic susceptibility curves measured under different applied magnetic fields. A strong field dependence of T_B (blocking temperature) is observed as expected and in good agreement with the related $Fe_3O_4@SiO_2@(mim)[FeCl_4]$.[1] b) Hysteresis loop at 2 K showing typical magnetization values for NPs. Top inset shows the enlarged low field region, where the FM domain contribution is evidenced. The high field region is enlarged in the bottom inset, showing field induced metamagnetism at H_C ~ 3 T.

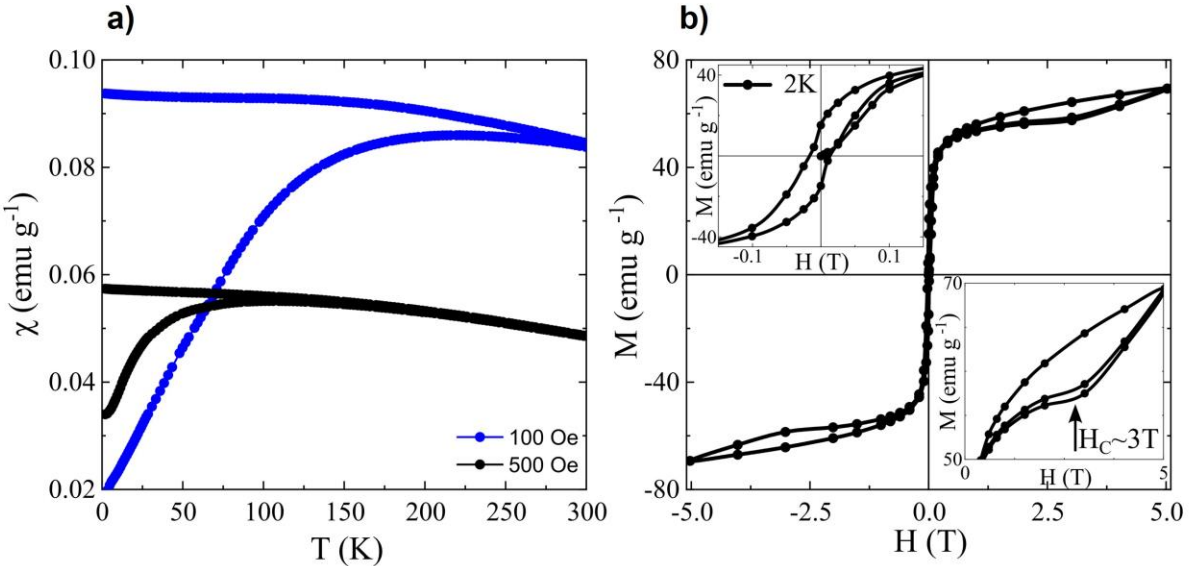

Field dependent magnetization shows no saturation, but a smooth increase supporting the presence of an AFM matrix. The origin of this matrix could be suspected to arise from a minor impurity of oxidized Fe_2O_3, not observed from the structural characterization techniques. The presence of such an impurity is further discarded due to the low maximal magnetization, which corresponds to 2.87 μ_B/f.u., well below the expected ordered magnetic moment of 4 μ_B/f.u. for Fe_3O_4. Our data point, otherwise, to a notable size effect, where the AFM interactions between the FM sublattices compete with the net FM contribution due to the short-range order. The random distribution of FM domains favoured by weak dipolar interactions is blocked below T_B, requiring a relatively high critical field of 3 T to align all domains, and giving rise to the observed metamagnetic transition. The FM nature of the domains justifies the hysteretic feature in this high magnetic field region.

4. N_2 adsorption-desorption isotherm studies

Figure S14. N_2 adsorption-desorption isotherm at 77 K of Fe_3O_4@SiO_2@(mim)[ZnCl(OH)$_2$].

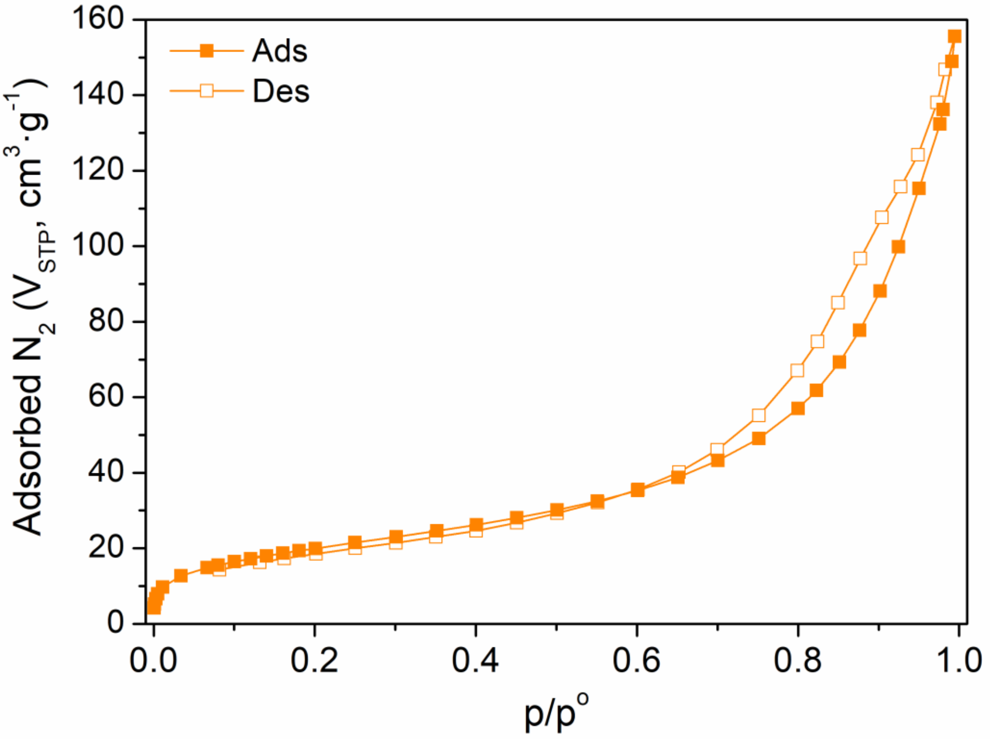

Figure S15. BET plot for $Fe_3O_4@SiO_2@(mim)[ZnCl(OH)_2]$ nanocatalyst.

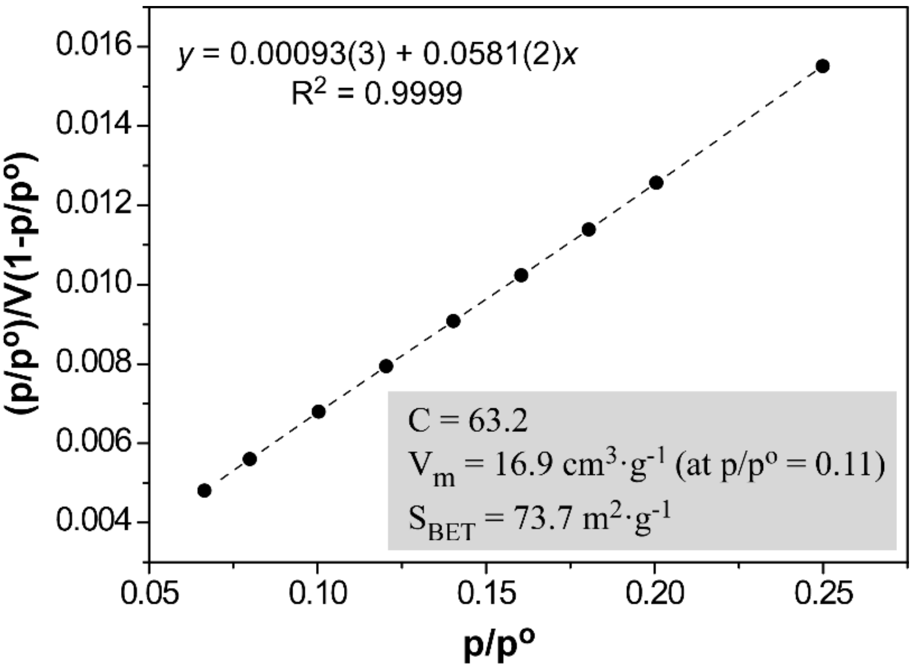

The surface area values were calculated by fitting the adsorption data to Brunauer-Emmett-Teller (BET) Equation S1:[2]

$$\frac{p/p^o}{V(1-p/p^o)} = \frac{1}{V_m C} + \frac{C-1}{V_m C}\left(\frac{p}{p^o}\right) \qquad \text{(Equation S1)}$$

where V is the specific amount adsorbed at the relative pressure p/p^o, V_m is the specific amount adsorbed corresponding to the monolayer formation, and C is a parameter exponentially related to the energy of monolayer formation.

The BET fitting data from the appropriate linear range (p/p^o = 0.05–0.30) for a Type II isotherm[3] are shown in Figure S10, along with the values of the monolayer volume V_m, the BET constant C and the surface area (S_{BET}) extracted during the analysis of the N_2 isotherm of the catalyst measured at 77 K.

5. FT-IR spectra of Fe_3O_4@SiO_2@(mim)[ZnCl(OH)$_2$]

Fe_3O_4@SiO_2@(mim)[ZnCl(OH)$_2$] NPs were characterized by Fourier transform infrared (FT-IR) spectroscopy (Figure S16). The peaks observed in the FT-IR spectrum confirm the composition of the Fe_3O_4 core and the presence of the Zn-containing IL on the SiO_2 shell: absorption bands at 552–580 cm^{-1} (Fe-O stretching vibration), peaks between 1224 and 890 cm^{-1} (Si-O and Si-O-Si), and absorption bands corresponding to the organic moiety of the IL at 1627 and 1566 cm^{-1} (C=N/C=C and C-N/C-C, respectively).[4,5]

Figure S16. IR spectrum of Fe_3O_4@SiO_2@(mim)[ZnCl(OH)$_2$].

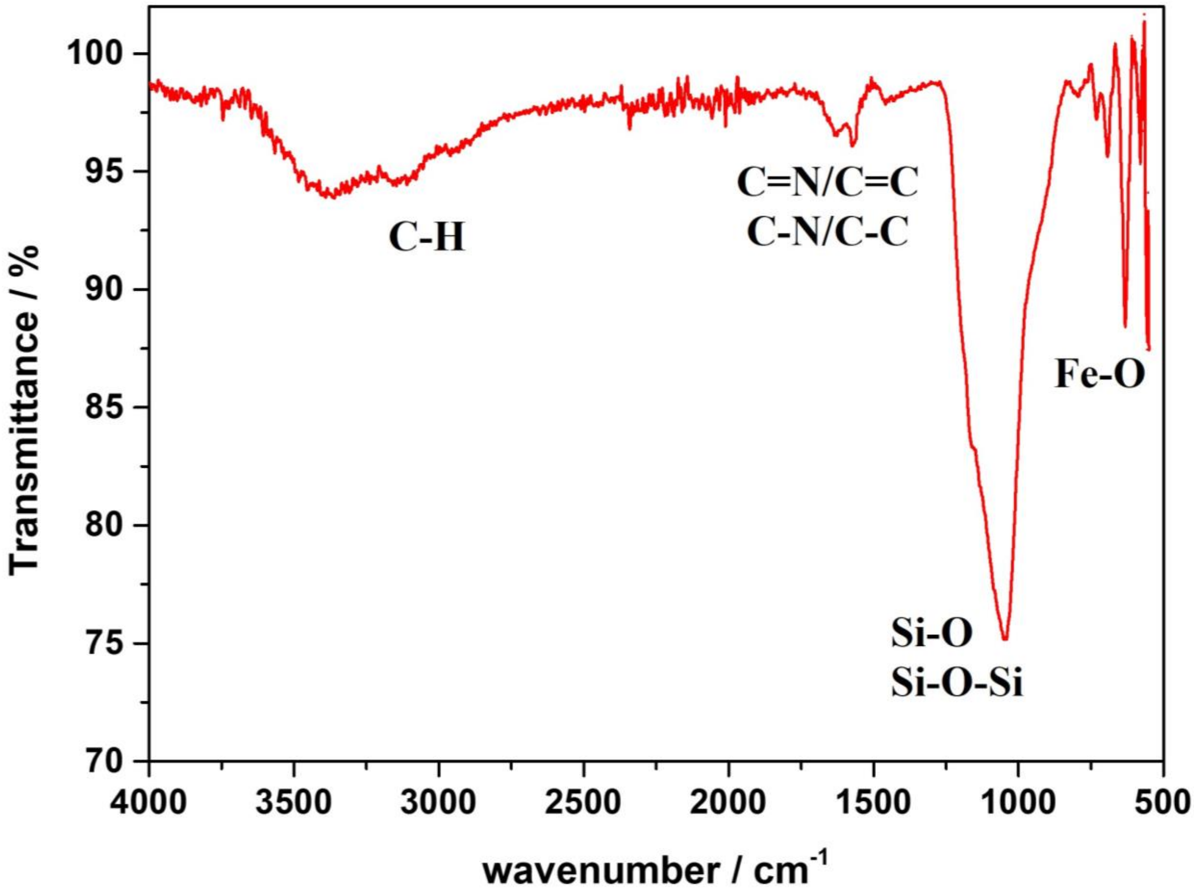

6. XEDS spectrum of Fe_3O_4@SiO_2@(mim)[ZnCl(OH)$_2$]

Figure S17. XEDS spectrum of Fe_3O_4@SiO_2@(mim)[ZnCl(OH)$_2$] showing the presence of Fe, Cl, Zn, Si and O. C and Cu belong to the holey carbon and Cu mesh TEM grid (Agar Scientific) used to support the nanoparticles.

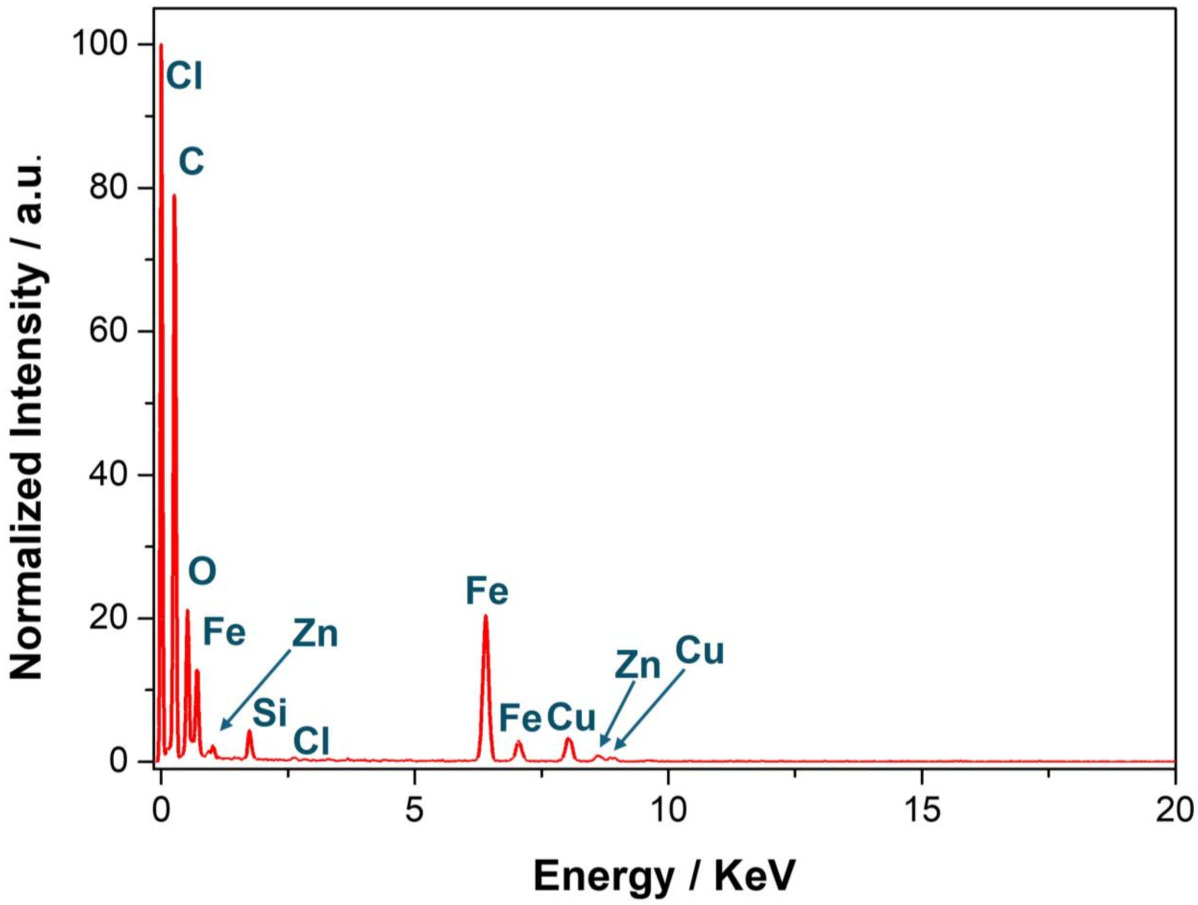

7. TGA analysis of $Fe_3O_4@SiO_2@(mim)[ZnCl(OH)_2]$

Figure S18. TGA analysis of $Fe_3O_4@SiO_2@(mim)[ZnCl(OH)_2]$.

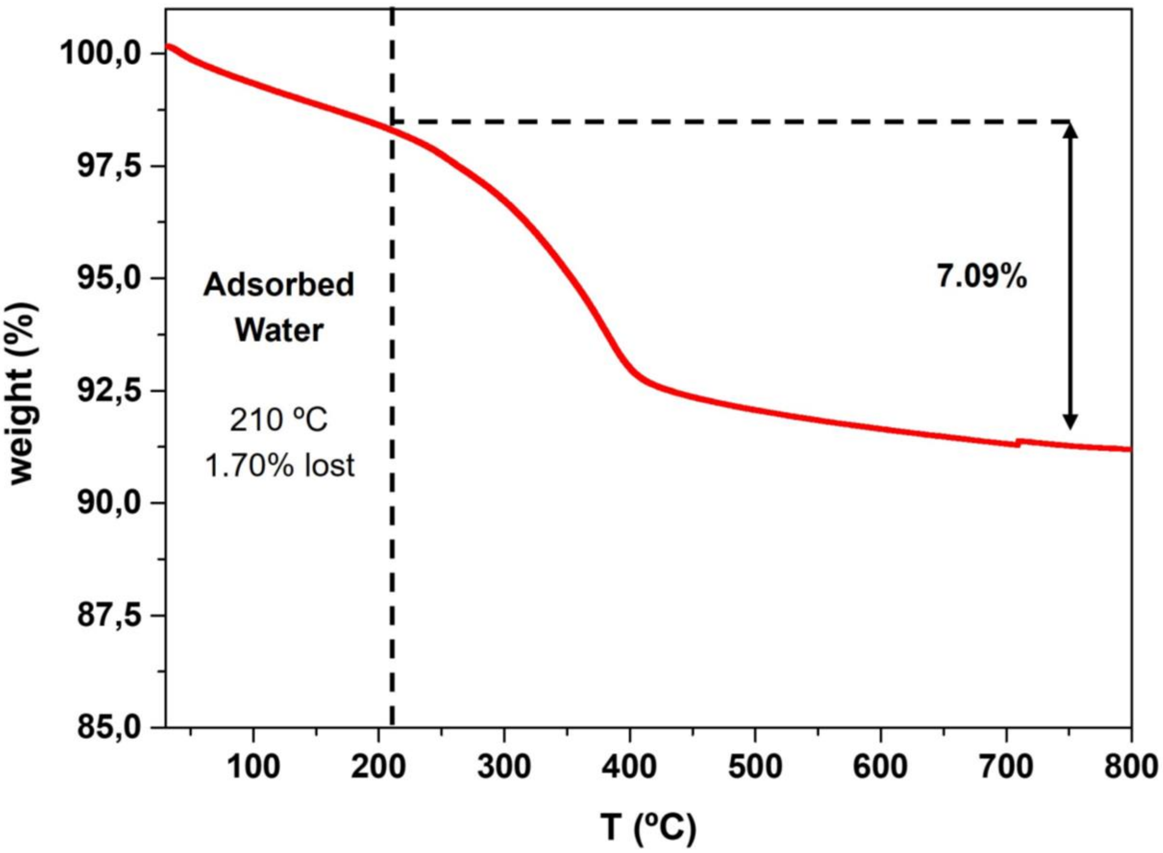

8. Characterization of BHET and BPA monomers

NMR spectroscopy. The ^1H and ^{13}C NMR spectra display the characteristic signals corresponding to BHET[6] and BPA[7] molecules. No peaks attributable to other compounds were observed (Figures S19-S24).

Figure S19. ^1H NMR spectrum of BHET monomer from PET (300 MHz, 298 K, DMSO-d_6): δ 8.12 (s, 4H, CH_{Ar}), 4.96 (t, J = 5.7 Hz, 2H, –OCH_2CH_2OH), 4.32 (t, J = 4.8 Hz, 4H, –OCH_2CH_2OH), 3.72 (m, 4H, –OCH_2CH_2OH).

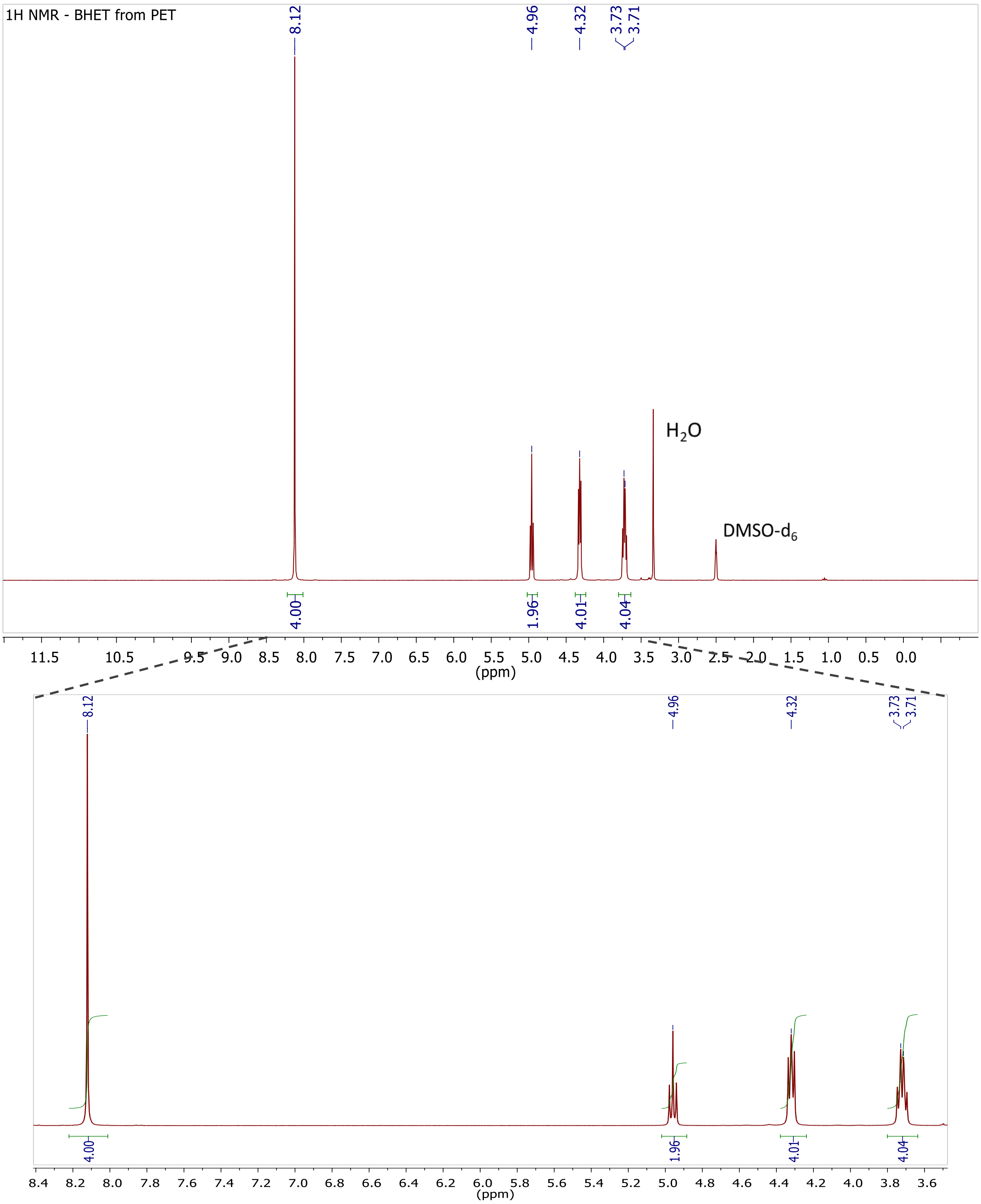

Figure S20. ^{13}C NMR spectrum of BHET monomer from PET (75 MHz, 298 K, DMSO-d_6): δ 165.2 (s, C_q, *C*=O), 133.8 (s, C_q, C_{Ar}), 129.5 (s, CH, CH_{Ar}), 67.0 (s, –O*C*H_2CH_2OH), 59.0 (s, –OCH_2*C*H_2OH).

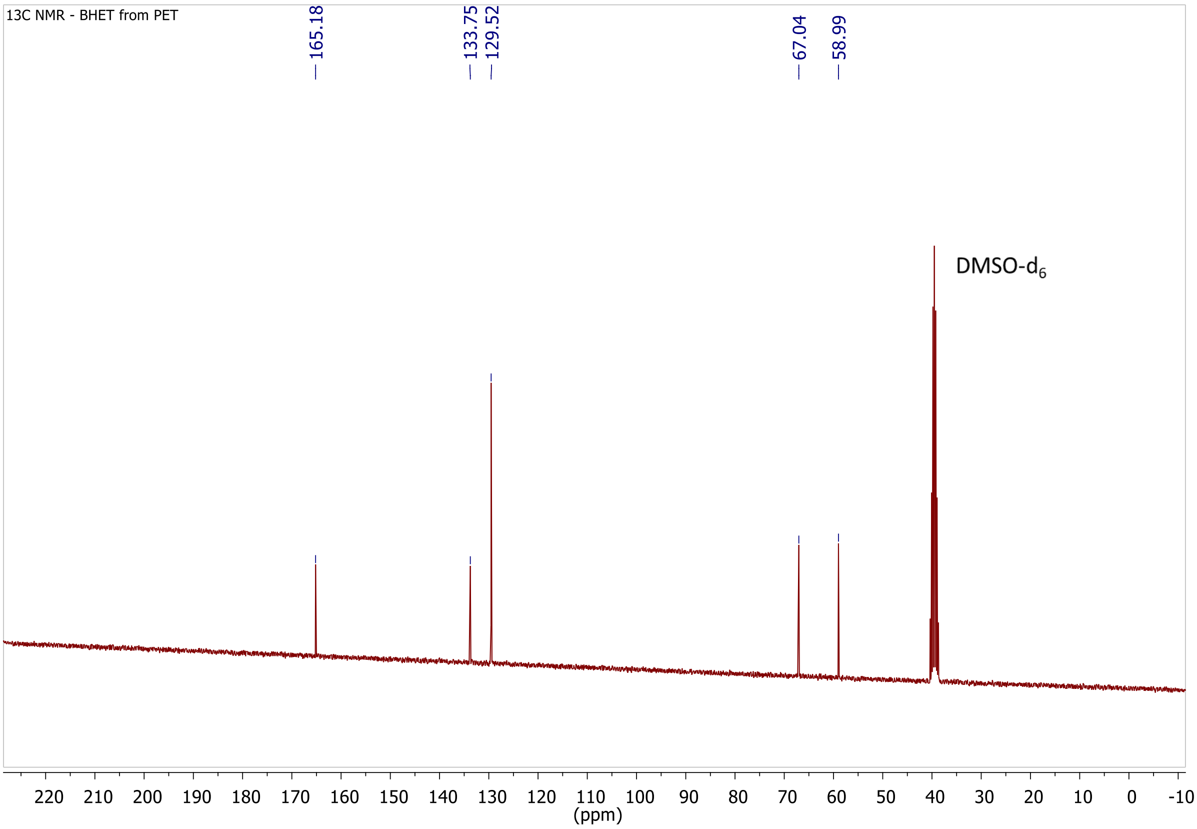

Figure S21. ^{1}H NMR spectrum of BHET monomer from PBT (300 MHz, 298 K, DMSO-d_6): δ 8.12 (s, 4H, CH_{Ar}), 4.96 (t, J = 5.7 Hz, 2H, –OCH_2CH_2OH), 4.32 (t, J = 4.8 Hz, 4H, –OCH_2CH_2OH), 3.72 (m, 4H, –OCH_2CH_2OH).

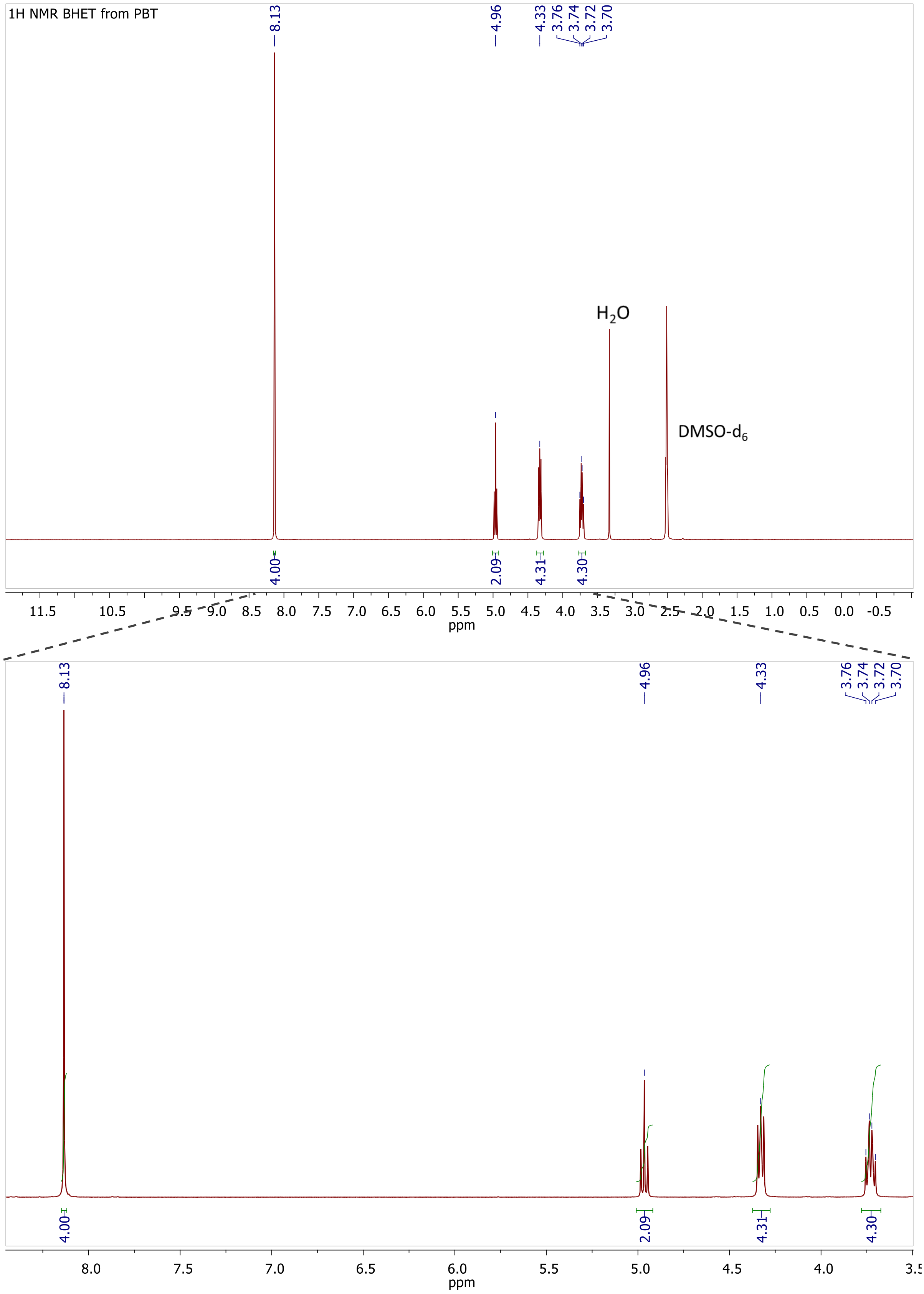

Figure S22. ^{13}C NMR spectrum of BHET monomer from PBT (75 MHz, 298 K, DMSO-d_6): δ 165.2 (s, C_q, *C*=O), 133.8 (s, C_q, C_{Ar}), 129.5 (s, CH, CH_{Ar}), 67.0 (s, –O*C*H_2CH_2OH), 59.0 (s, –OCH_2*C*H_2OH).

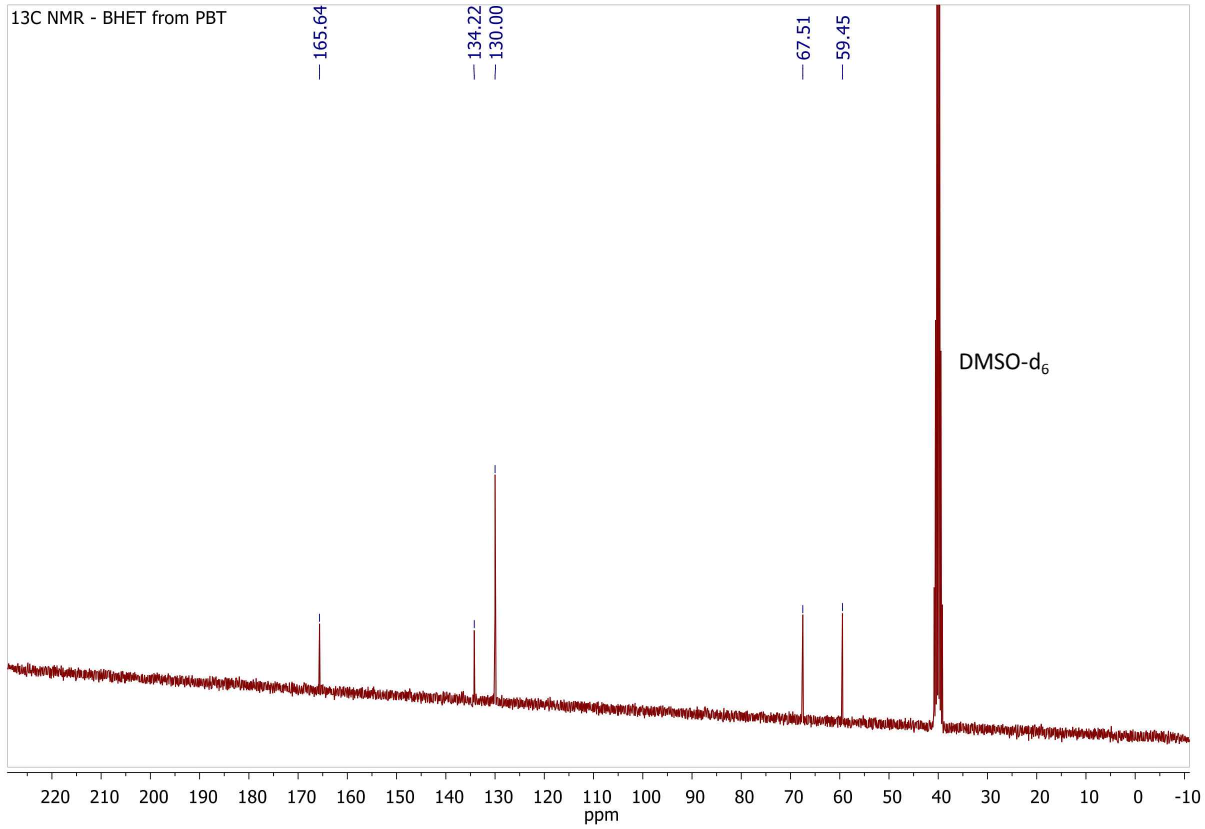

Figure S23. ^{1}H NMR spectrum of BPA monomer from BPA-PC (300 MHz, 298 K, DMSO-d_6): δ 9.12 (s br, 2H, O*H*), 6.97 (d, *J* = 8.7 Hz, 4H, CH_{Ar}), 6.63 (d, *J* = 8.7 Hz, 4H, CH_{Ar}), 1.52 (s, 6H, CH_3).

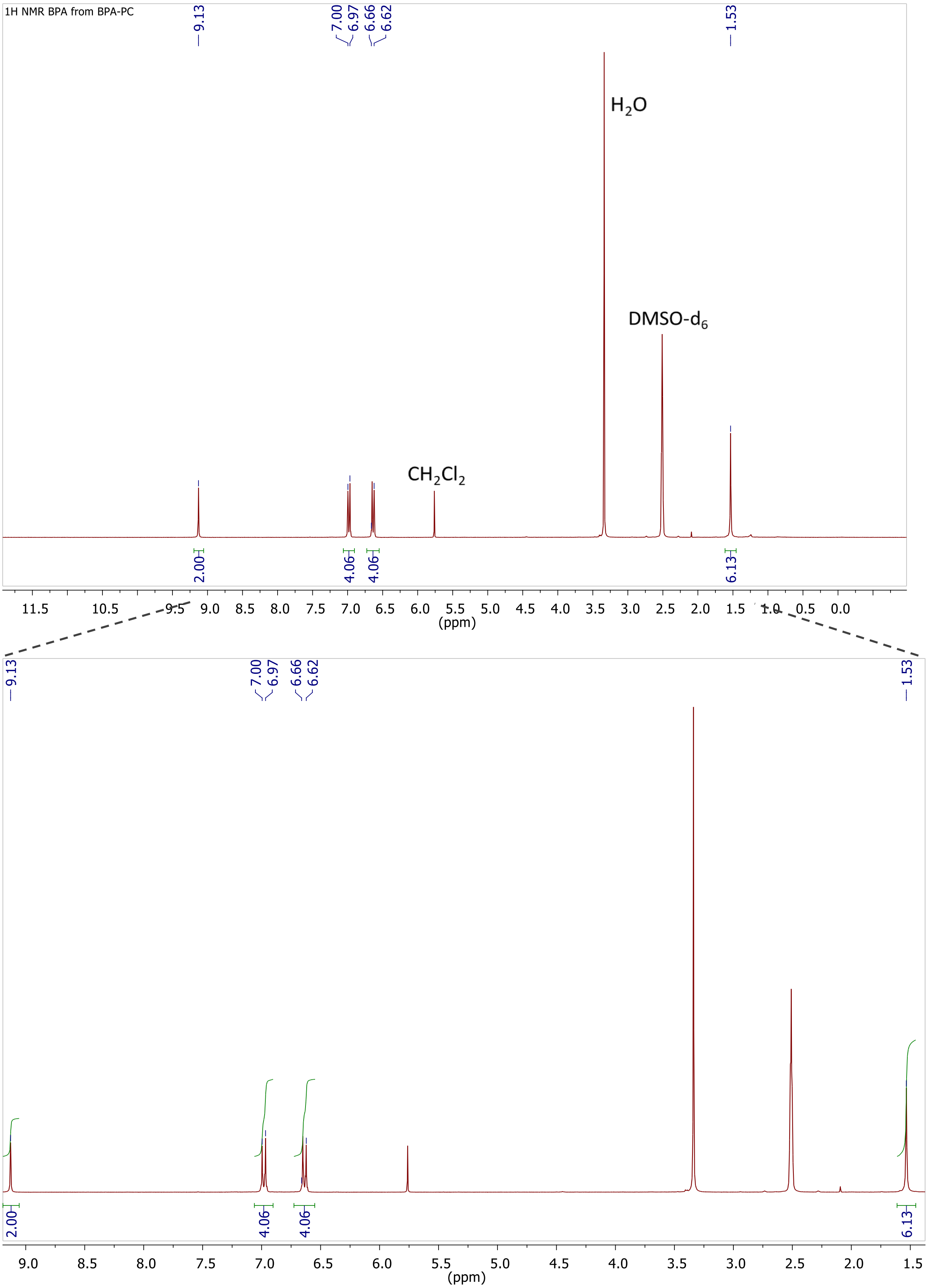

Figure S24. ^{13}C NMR spectrum of BPA monomer from BPA-PC (75 MHz, 298 K, DMSO-d_6): δ 154.8 (s, C_q, C_{Ar}), 141.1 (s, C_q, C_{Ar}), 127.2 (s, CH, CH_{Ar}), 114.5 (s, CH, CH_{Ar}), 40.9 (s, C_q, $C(CH_3)_2(C_{Ar})_2$), 30.8 (s, CH_3, $–C(CH_3)_2$).

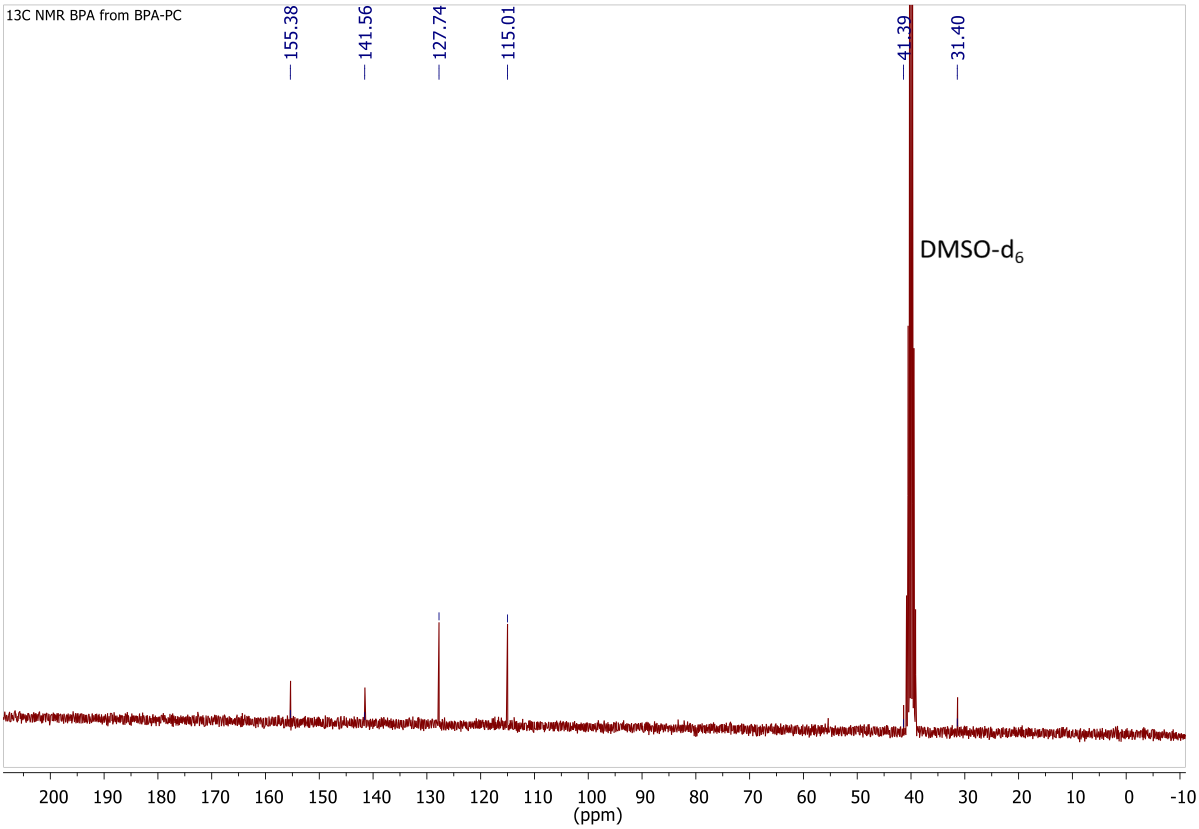

FT-IR spectroscopy. The FT-IR spectra of BHET and BPA (Figures S25-S27) are in good accordance with those previously described.[6,8]

Figure S25. IR spectrum of BHET monomer from PET.

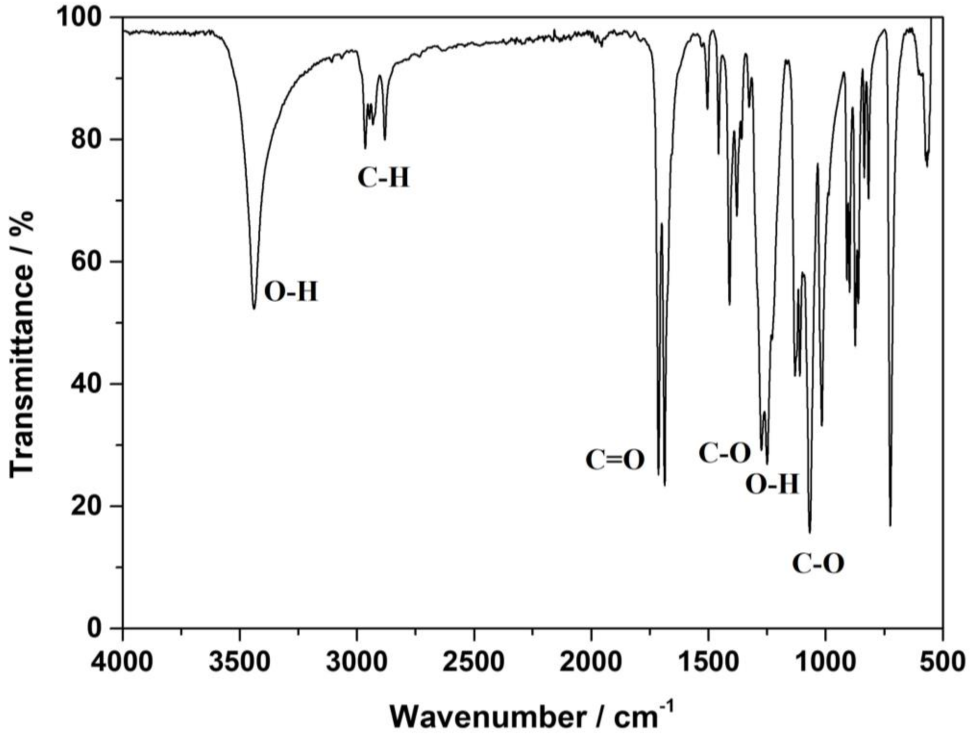

Figure S26. IR spectrum of BHET monomer from PBT.

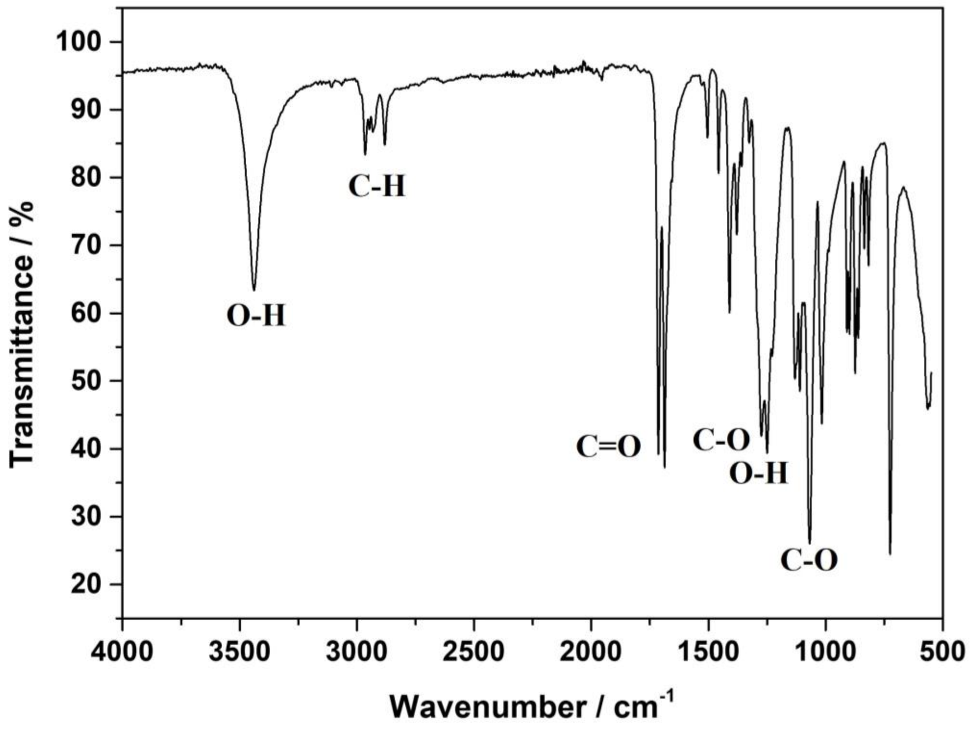

Figure S27. IR spectrum of BPA monomer from BPA-PC.

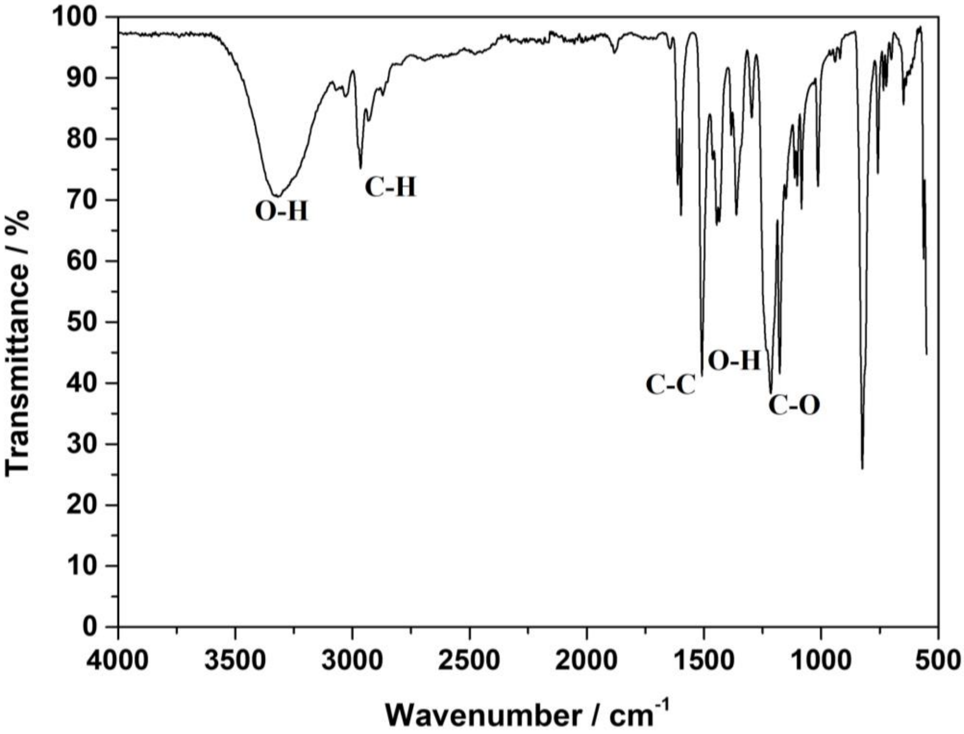

Thermal analyses (DSC and TGA). The DSC curve displays sharp endothermic peaks for BHET and BPA monomers at *ca.* 114 ºC and 156 ºC, respectively. These data are in good agreement with the reported melting points of the monomers (Figures S28-S30).[9,10,11] The TGA curves (Figure S31-S33) show the thermal decomposition of BHET and BPA products.[6]

Figure S28. DSC analysis of BHET monomer from PET.

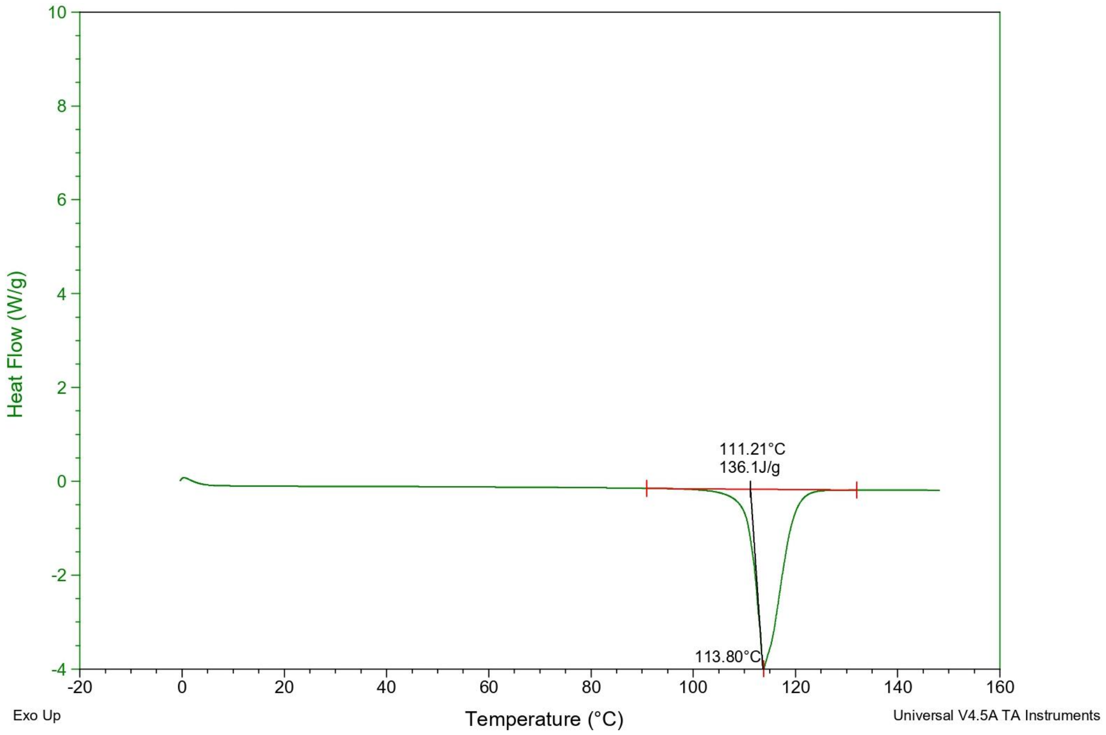

Figure S29. DSC analysis of BHET monomer from PBT.

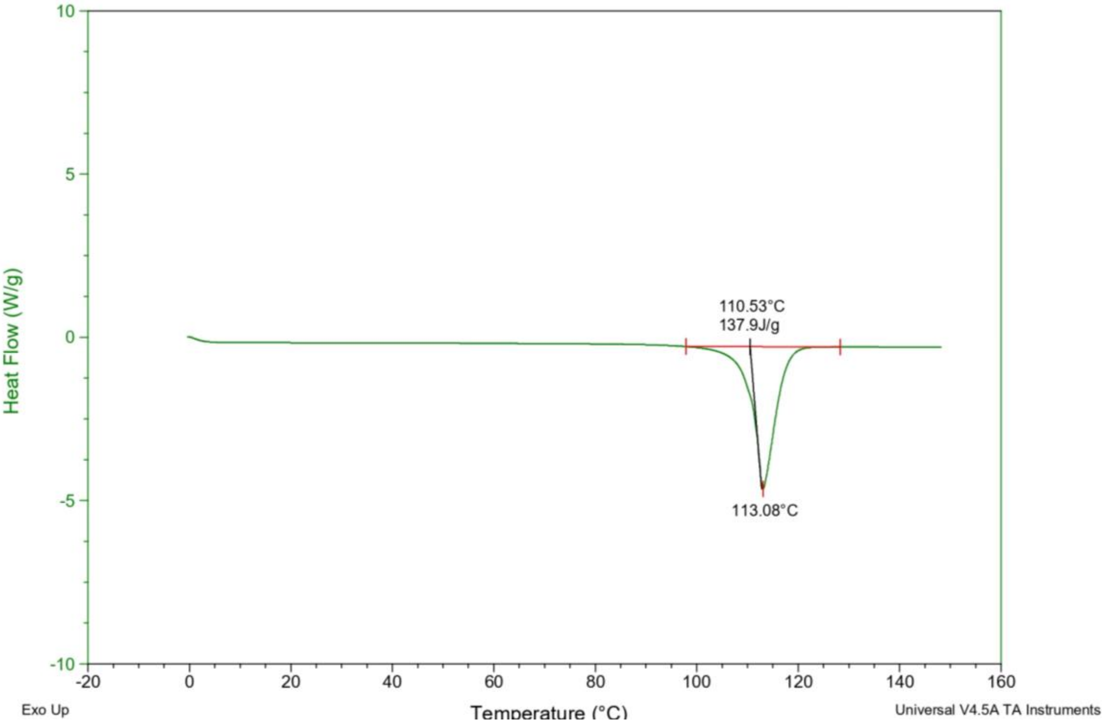

Figure S30. DSC analysis of BPA monomer from BPA-PC.

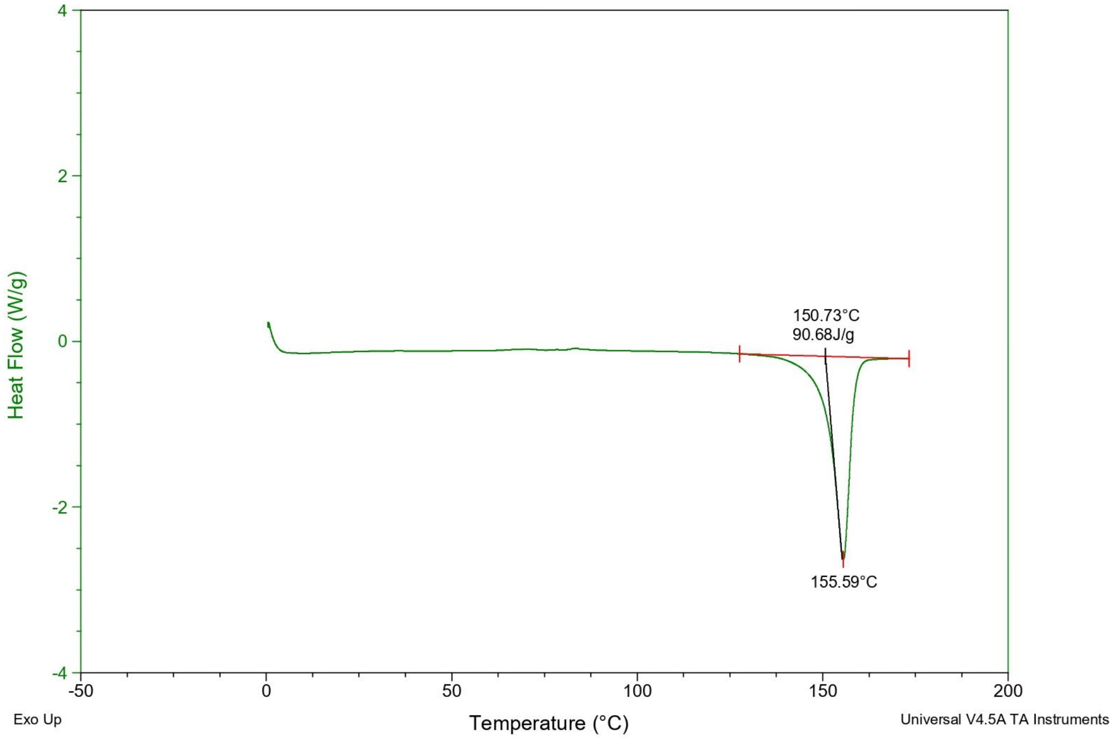

Figure S31. TGA analysis of BHET monomer from PET.

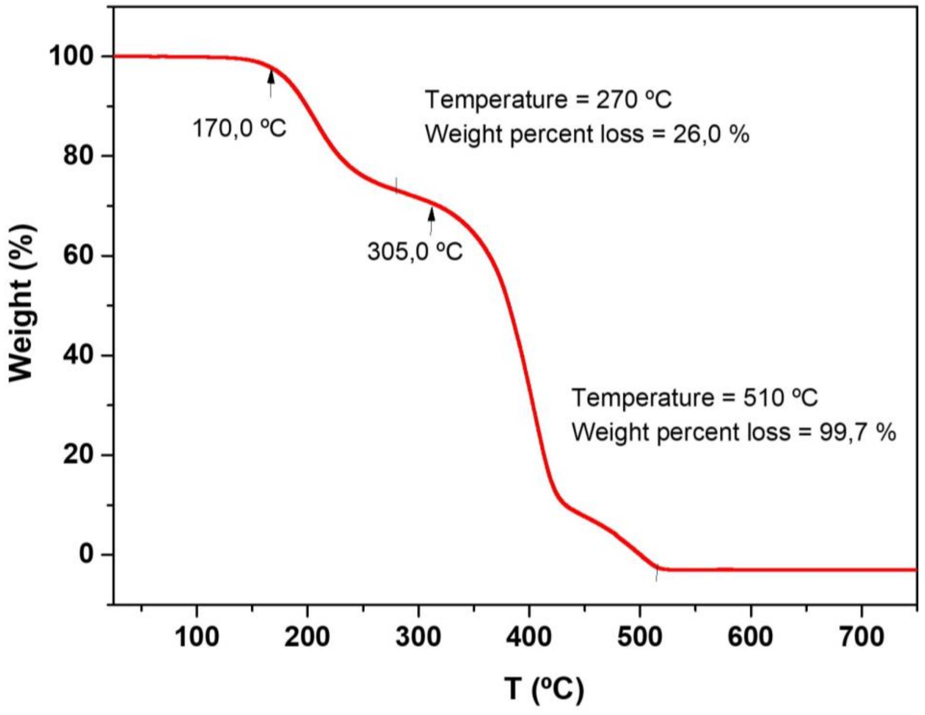

Figure S32. TGA analysis of BHET monomer from PBT.

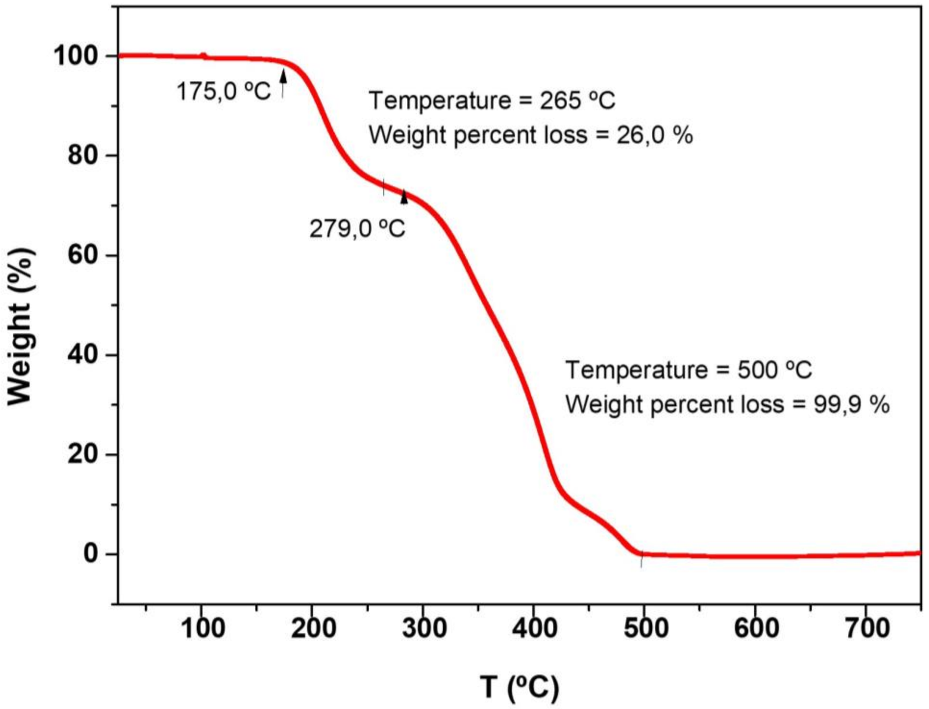

Figure S33. TGA analysis of BPA monomer from BPA-PC.

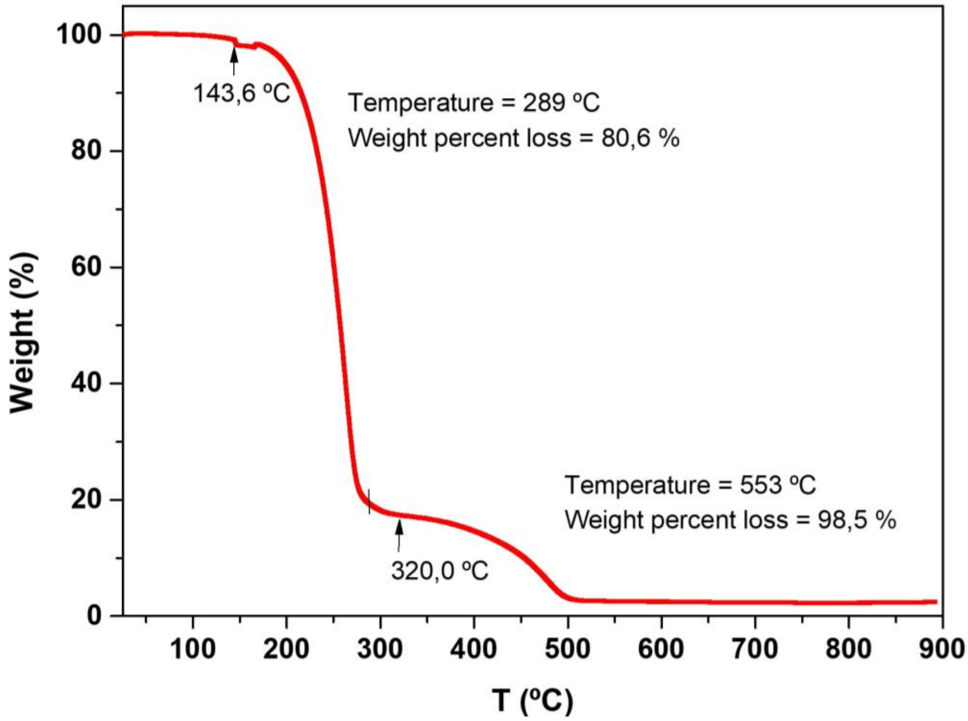

9. Gram-scale experiments

Gram-scale reactions with 2 g of PET were performed, leading to quantitative PET consumption and *ca*. 80% isolated yield towards BHET in the first and second catalytic cycles. The purity of the isolated BHET product was demonstrated by NMR spectroscopy (Figures S34-S35).

Figure S34. ^{1}H NMR spectrum of BHET monomer from PET (300 MHz, 298 K, DMSO-d_6). First catalytic cycle.

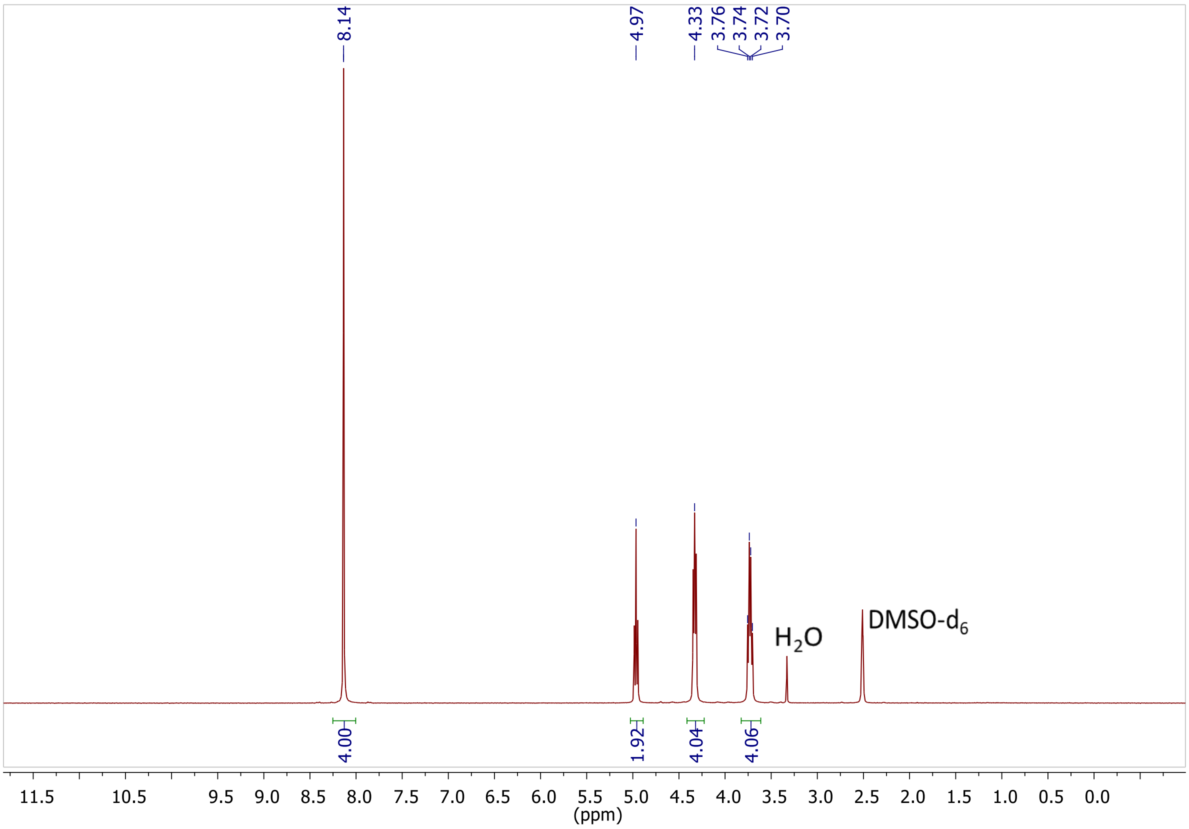

^{1}H NMR (300 MHz, 298 K, DMSO-d_6): δ 8.12 (s, 4H, CH_{Ar}), 4.96 (t, J = 5.7 Hz, 2H, –OCH_2CH_2O*H*), 4.32 (t, J = 4.8 Hz, 4H, –OC$H_2$$CH_2OH$), 3.72 (m, 4H, –$OCH_2CH_2$OH).

Figure S35. ^{1}H NMR spectrum of BHET monomer from PET (300 MHz, 298 K, DMSO-d_6). Second catalytic cycle.

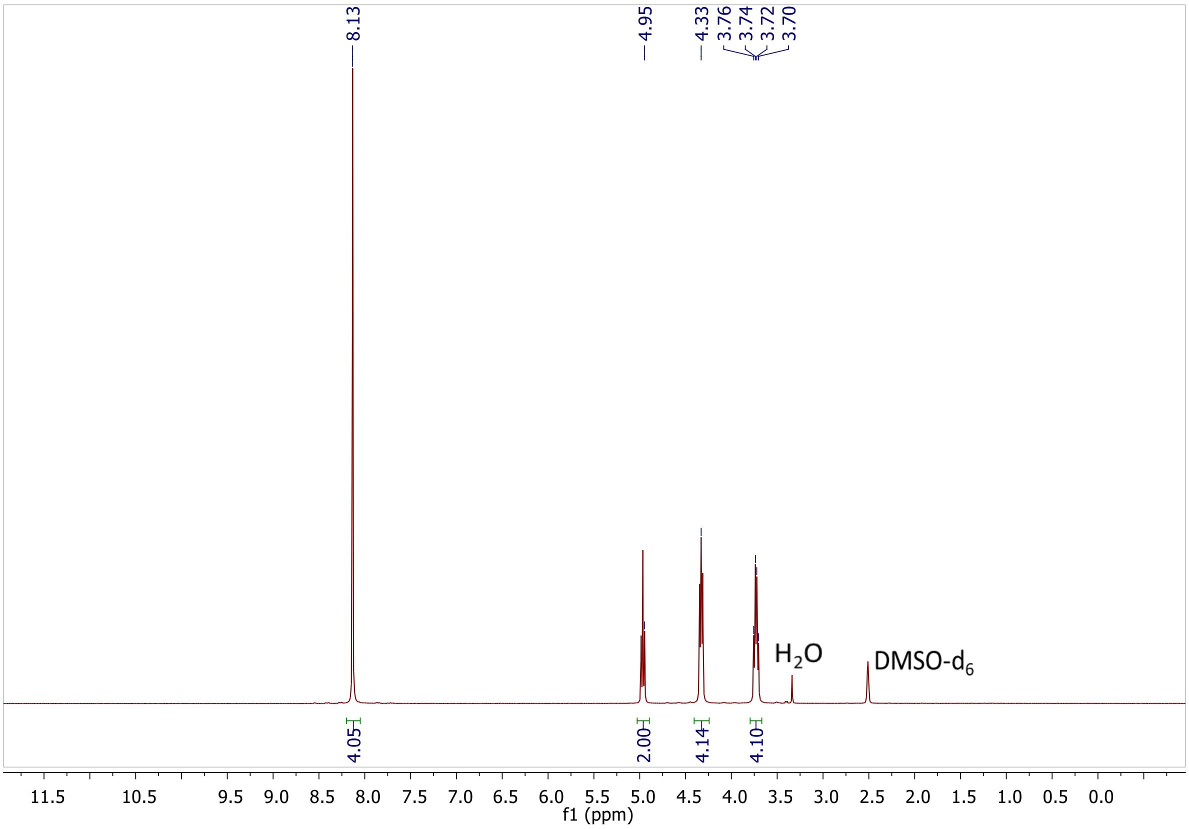

^{1}H NMR (300 MHz, 298 K, DMSO-d_6): δ 8.12 (s, 4H, CH_{Ar}), 4.96 (t, J = 5.7 Hz, 2H, –OCH$_2$CH$_2$OH), 4.32 (t, J = 4.8 Hz, 4H, –OCH_2CH$_2$OH), 3.72 (m, 4H, –OCH$_2$CH_2OH).

Figure S36. Crystalline BHET product obtained after the work-up.

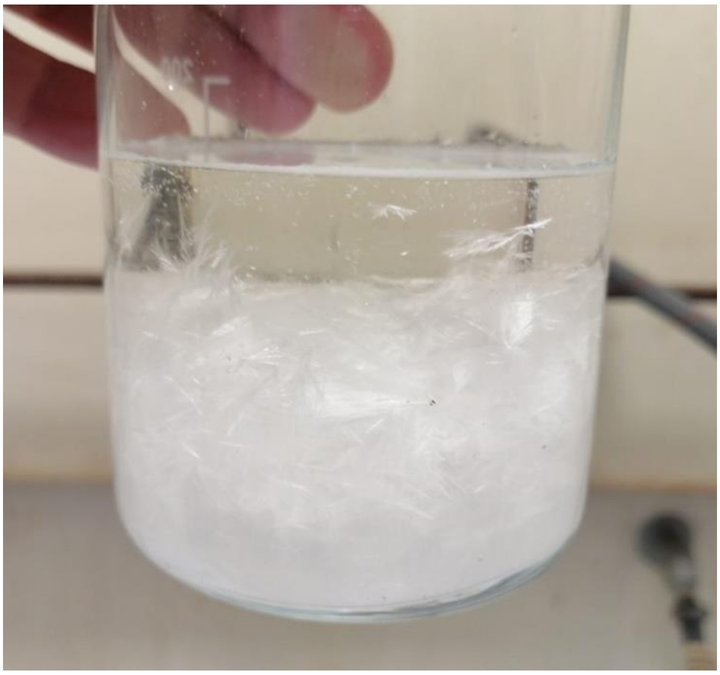

Gram-scale reactions with 2 g of PBT were performed, leading to nearly complete conversion of PBT consumption and *ca*. 84% isolated yield towards BHET in the first and second catalytic cycles. The purity of the isolated BHET product was demonstrated by NMR spectroscopy (Figures S37-S38).

Figure S37. ^{1}H NMR spectrum of BHET monomer from PBT (300 MHz, 298 K, DMSO-d_6). First catalytic cycle.

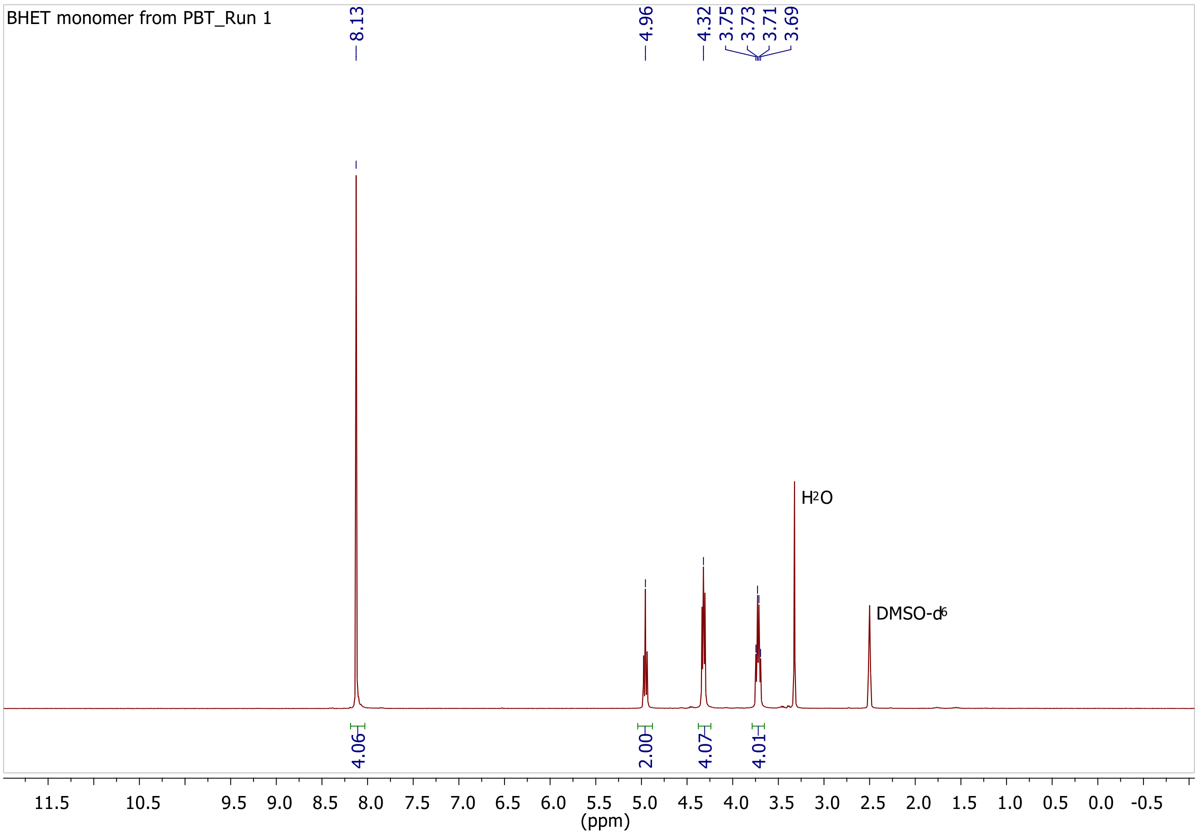

^{1}H NMR (300 MHz, 298 K, DMSO-d_6): δ 8.12 (s, 4H, CH_{Ar}), 4.96 (t, J = 5.7 Hz, 2H, –OCH$_2$CH$_2$OH), 4.32 (t, J = 4.8 Hz, 4H, –OCH_2CH$_2$OH), 3.72 (m, 4H, –OCH$_2$CH_2OH).

Figure S38. ^{1}H NMR spectrum of BHET monomer from PBT (300 MHz, 298 K, DMSO-d_6). Second catalytic cycle.

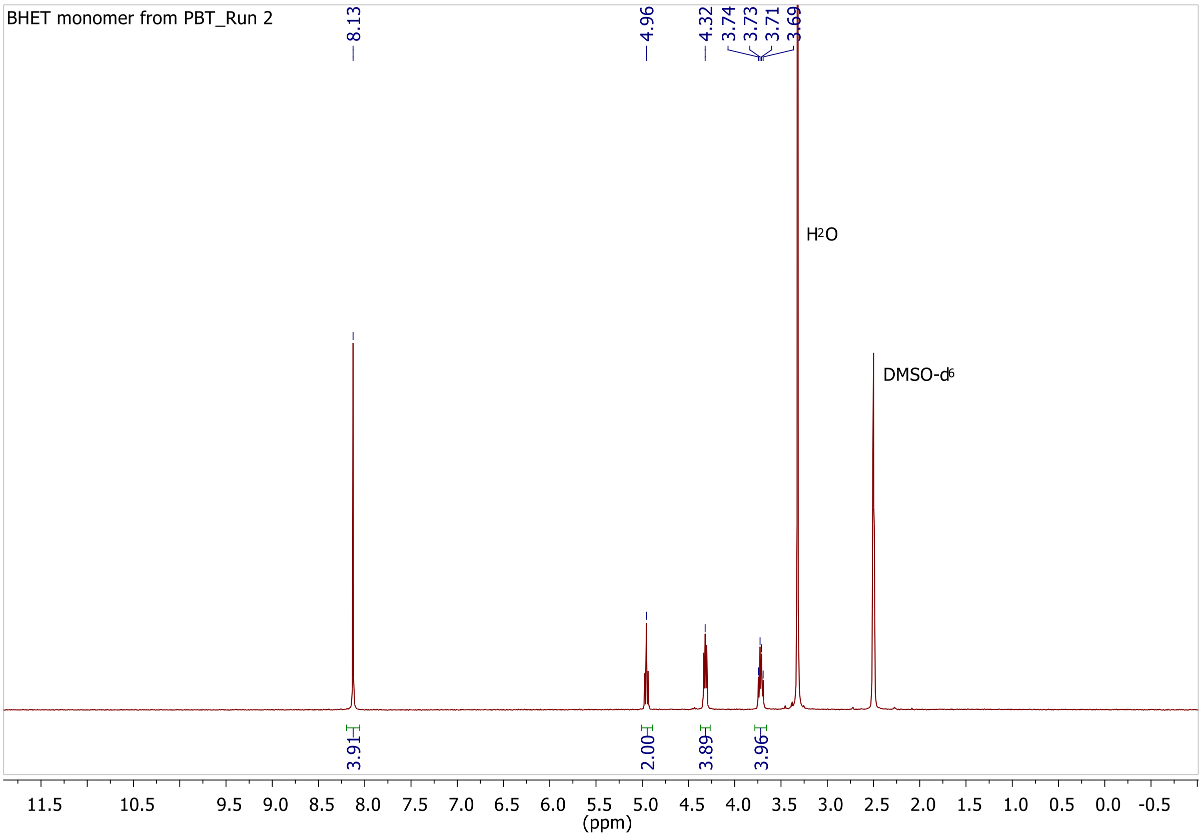

^{1}H NMR (300 MHz, 298 K, DMSO-d_6): δ 8.12 (s, 4H, CH_{Ar}), 4.96 (t, J = 5.7 Hz, 2H, –OCH_2CH_2OH), 4.32 (t, J = 4.8 Hz, 4H, –OCH_2CH_2OH), 3.72 (m, 4H, –OCH_2CH_2OH).

Gram-scale reactions with 2 g of BPA-PC were performed, leading to quantitative BPA-PC consumption and *ca*. 60% isolated yield towards BHET in the first and second catalytic cycles. The purity of the isolated BHET product was demonstrated by NMR spectroscopy (Figures S39-S40).

Figure S39. ^{1}H NMR spectrum of BPA monomer from BPA-PC (300 MHz, 298 K, DMSO-d_6). First catalytic cycle.

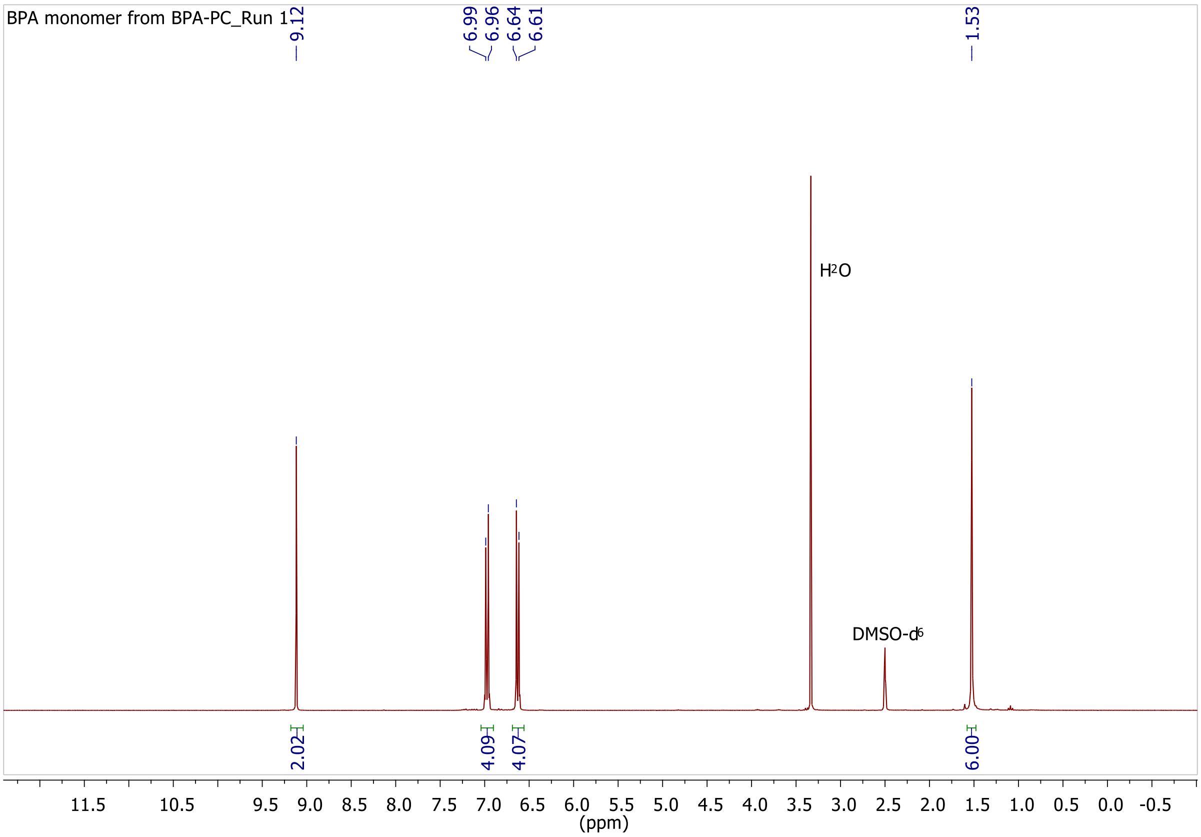

^{1}H NMR (300 MHz, 298 K, DMSO-d_6): δ 9.12 (s br, 2H, O*H*), 6.97 (d, *J* = 8.7 Hz, 4H, CH_{Ar}), 6.63 (d, *J* = 8.7 Hz, 4H, CH_{Ar}), 1.52 (s, 6H, CH_3).

Figure S40. ^{1}H NMR spectrum of BPA monomer from BPA-PC (300 MHz, 298 K, DMSO-d_6). Second catalytic cycle.

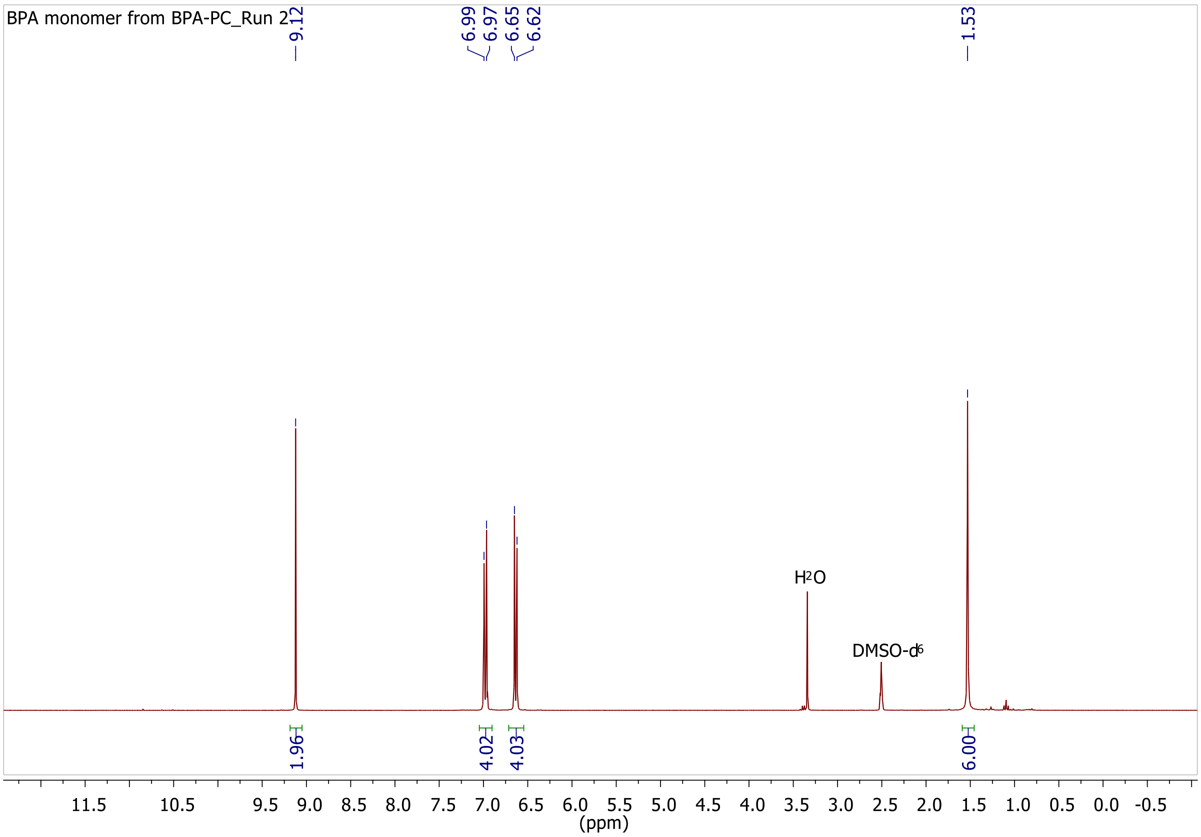

^{1}H NMR (300 MHz, 298 K, DMSO-d_6): δ 9.12 (s br, 2H, O*H*), 6.97 (d, *J* = 8.7 Hz, 4H, CH_{Ar}), 6.63 (d, *J* = 8.7 Hz, 4H, CH_{Ar}), 1.52 (s, 6H, CH_3).

10. TEM images of nanoparticles employed in the control experiments

Figure S41. TEM image of Fe_3O_4.

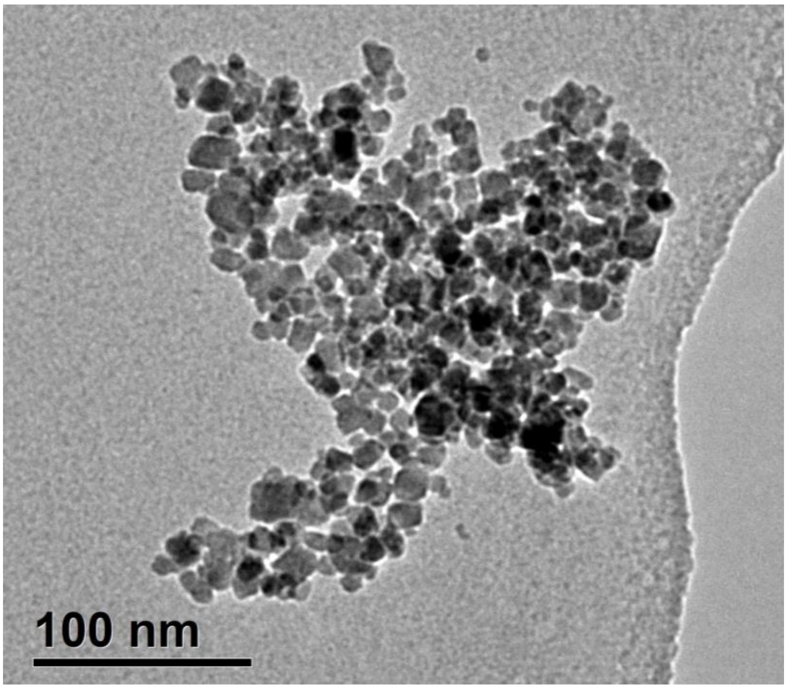

Figure S42. TEM image of Fe_3O_4.

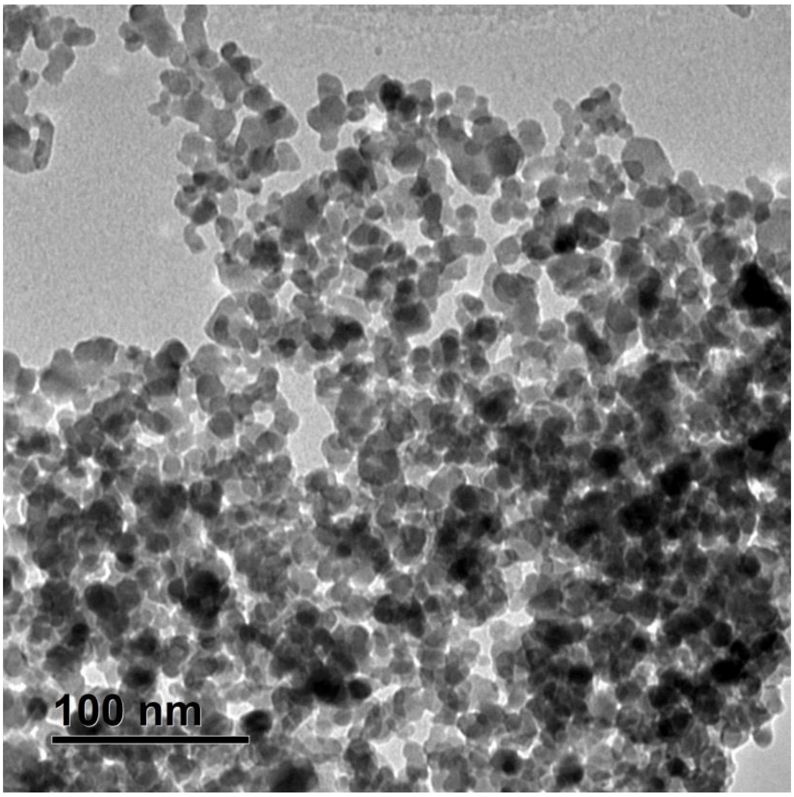

Figure S43. TEM image of Fe_3O_4@SiO_2.

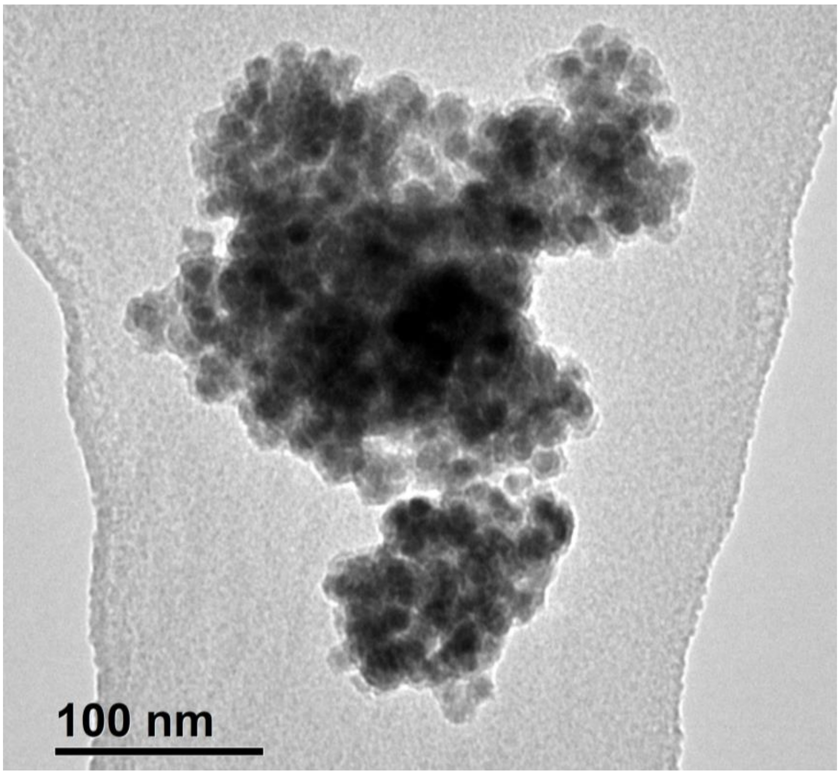

Figure S44. TEM image of Fe_3O_4@SiO_2.

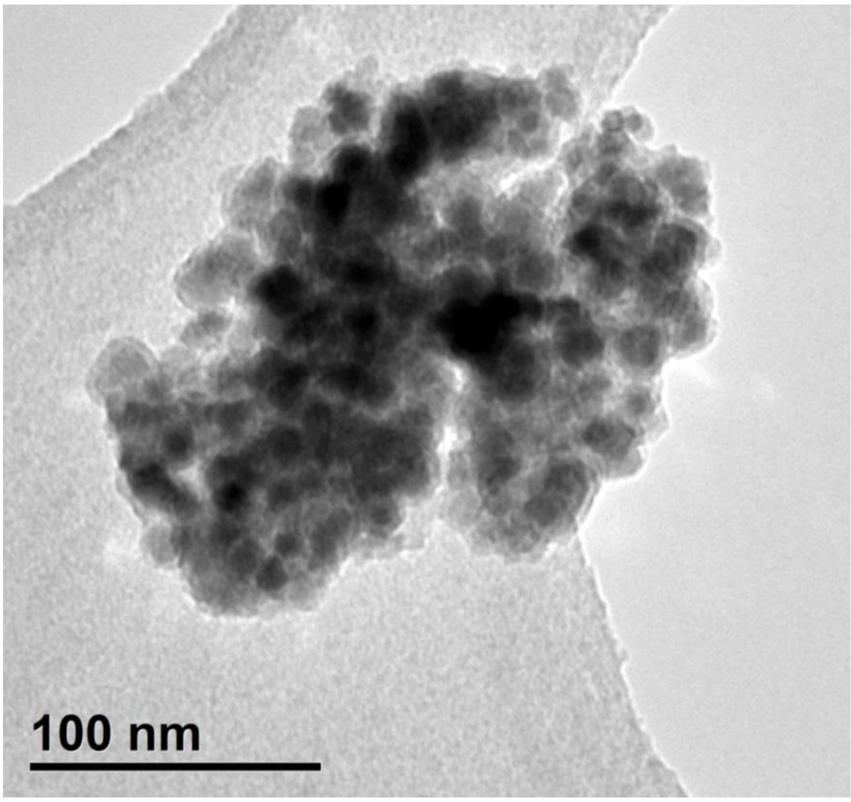

Figure S45. TEM image of Fe_3O_4@SiO_2@(mim)Cl.

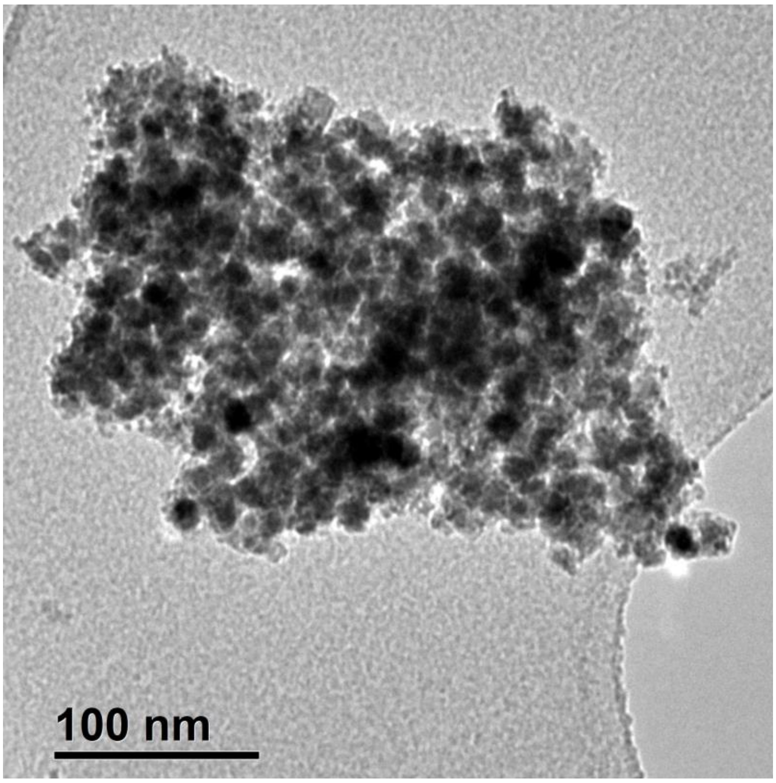

Figure S46. TEM image of Fe_3O_4@SiO_2@(mim)Cl.

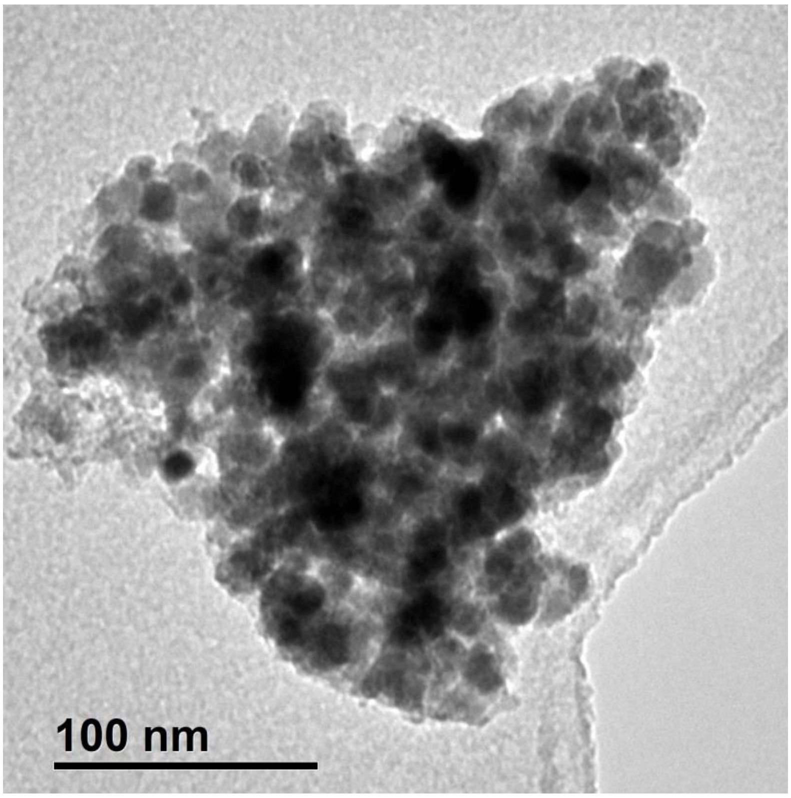

Figure S47. TEM image of Fe_3O_4@SiO_2@(mim)PF_6.

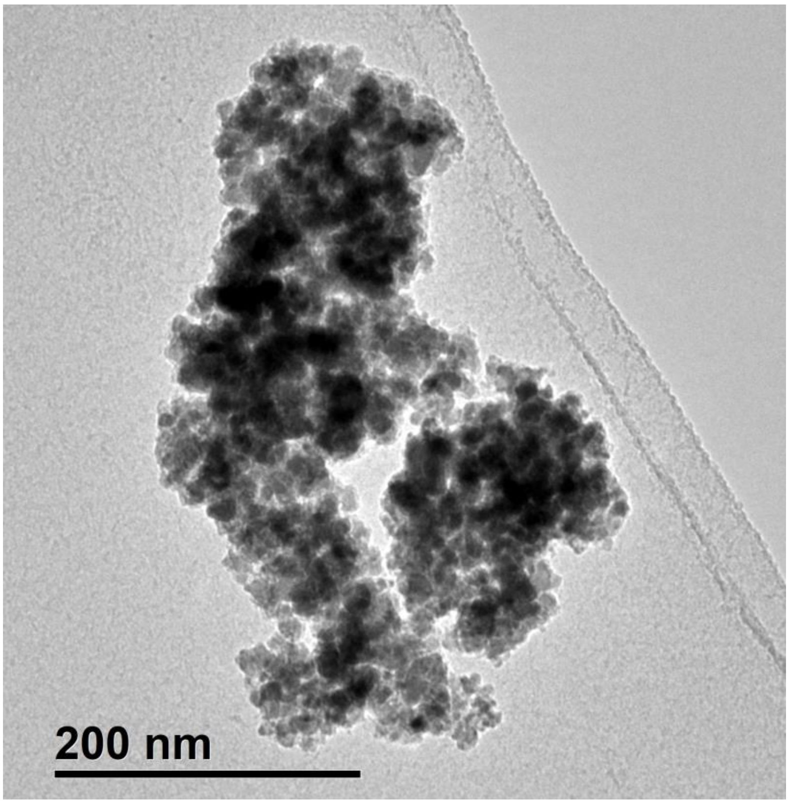

Figure S48. TEM image of Fe_3O_4@SiO_2@(mim)PF_6.

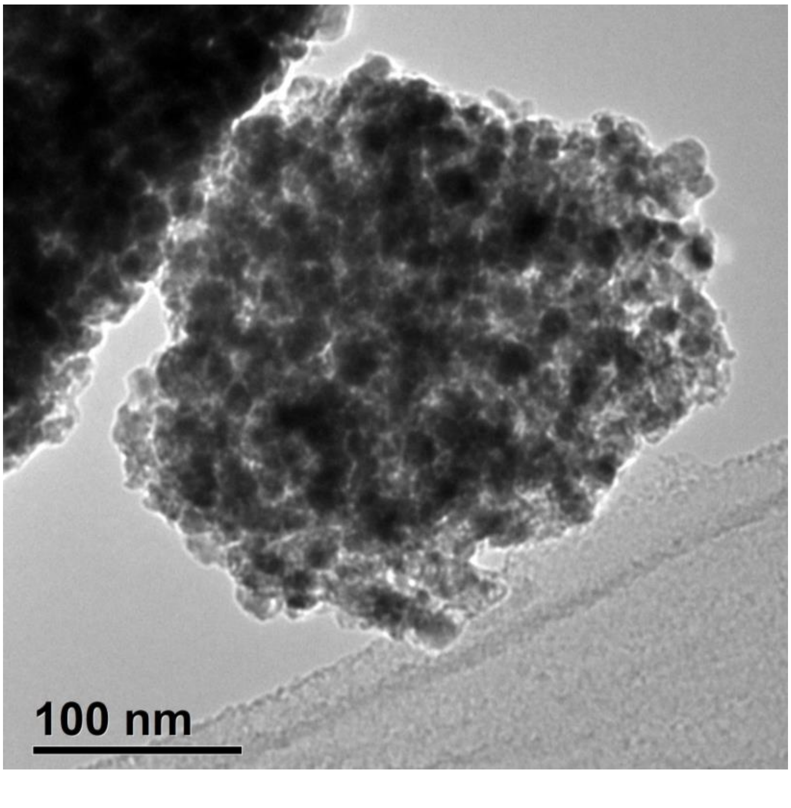

11. Comparative analysis with similar catalytic systems reported in the literature

Table S1. Comparison of several reported catalytic systems for PET glycolysis.

Catalyst	Catalyst loading (w:w)	EG:PET (w:w)	Time (h)	T (ºC)	BHET yield (%)	Runs	Ref.
$Fe_3O_4@SiO_2@(mim)[ZnCl(OH)_2]$	6:100 (0.39 mol%)[a]	1:9	24	170	>99	12	This work
Ionic liquids immobilized on magnetic supports							
$CoFe_2O_4/C_{10}$-OAc[b]	1:50	1:5	2.5	195	95.8	10	12
Fe_3O_4@PMIM.$SbBr_4$[c]	6:100 (0.21 mol%)[d]	1:5	1	200	96.4	5	13
$Fe_2O_3@SiO_2$-NH_2-SB<br>$Fe_2O_3@SiO_2$-NH-NH_2	1:10	1:4.5	3	190	73<br>71	5	14
Magnetic nanoparticles							
γ-Fe_2O_3	1:20	1:3	1	300	>90	10	15
Fe_3O_4/MWCNT	1:20	1:10	2	190	>99	8	16
$CoFe_2O_4$	1:143	1:8	1	210	92.5	4	17
Mg-Al-O@Fe_3O_4	1:200	1:5	1.5	240	82	3	18
Co-Al31@Fe_3O_4	1:100	1:9	2	180	99	4	19
Purely organic and metal-containing ionic liquids							
[Ch][OAc][e]	1:20 (5.9 mol%)	1:4	4	180	85.2	1	20
[Bmim−Fe][$(OAc)_3$]/ Bentonite	1:3[f]	1:6.7	3	190	44	6	21
$(dimim)_2[Fe_2Cl_6(\mu\text{-}O)]$	1:5.4 (6.5 mol%)	1:5.8	24<br>2[g]	170	74<br>77	2	22
$(dimim)_2[Fe_2Cl_4(\mu\text{-}ox)]$	1:5 (6.9 mol%)	1:5.4	24<br>3[h]	170	80<br>>99	2	23
$[bmim]_2[ZnCl_4]$<br>$[bmim]_2[CoCl_4]$	1:5 (2 mol%)<br>1:6 (6.6 mol%)	1:4<br>1:11.7	4<br>1.5	170<br>175	89.5<br>95.7	1<br>7	6
[deim][$Zn(OAc)_3$][i]	1:40 (1.2 mol%)	1:6.7	2.5	180	70.9	1	24

[a] Zn-containing IL loading (1:227 w/w). [b] C_{10}-OAC: [C_{10}COOHbim]OAc. [c] PMIM: propylmethylimidazolium. [d] Sb-containing IL loading. [e] Choline acetate. [f] The catalytic material contains 30% ionic liquid (wt%). [g] Microwave-assisted conditions (100 W). [h] Microwave-assisted conditions (6 W). [i] deim: 1,3-diethylimidazolium.

12. Characterization of 2 after catalysis

Figure S49. Compared XRPD data of **2** before (red) and after (blue) the catalytic reaction. A negligible increase in the cell parameter is observed, while the main difference between both samples is the increased background at low angles due to the amorphous contribution of oligomers and BHET monomer. Particle size from the Scherrer equation yields (9.0 ± 0.1) Å and (9.1 ± 0.2) Å before and after catalysis, respectively, which confirms that the catalytic process does not affect the magnetite core of the nanoparticles.

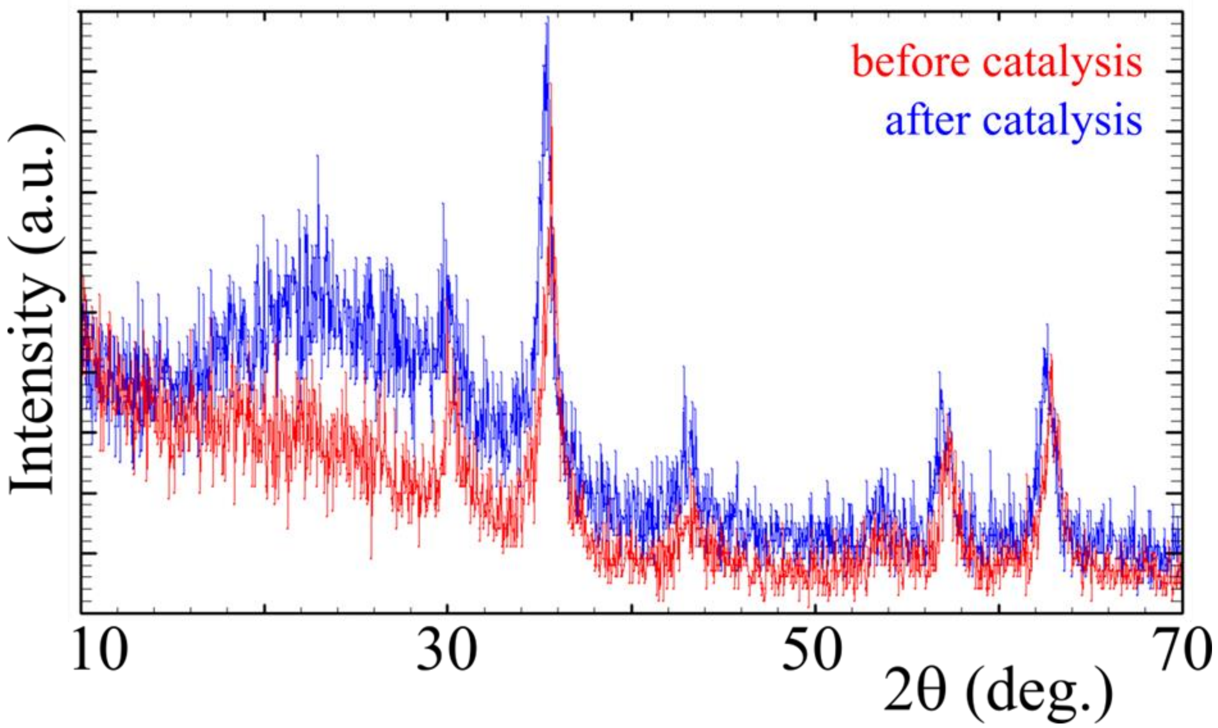

Figure S50. Low-magnification TEM image of **2** recovered after the catalytic reaction.

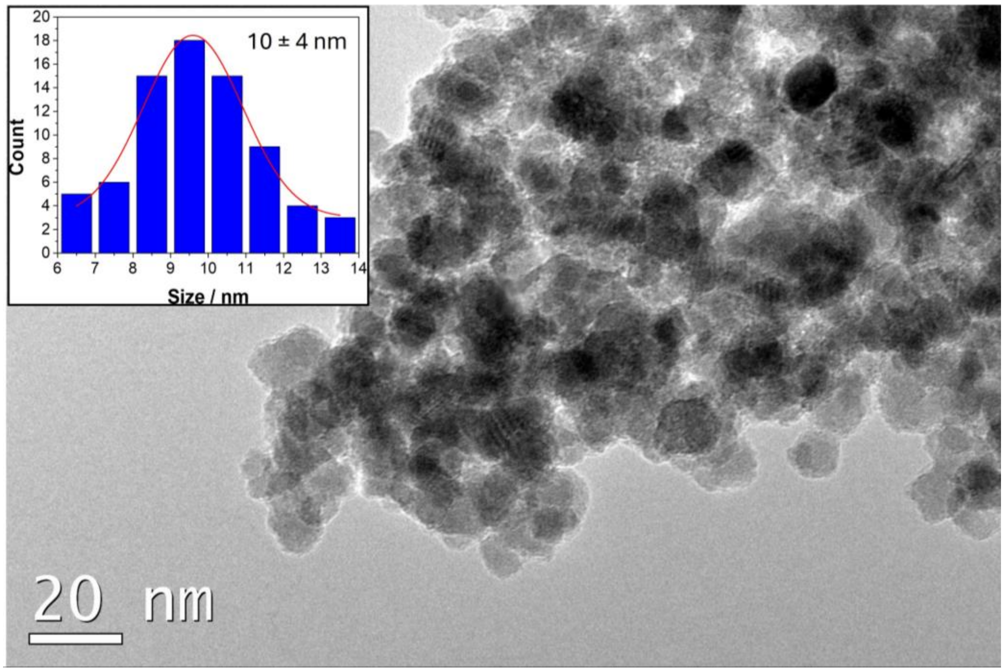

Figure S51. High-magnification TEM image of **2** recovered after the catalytic reaction.

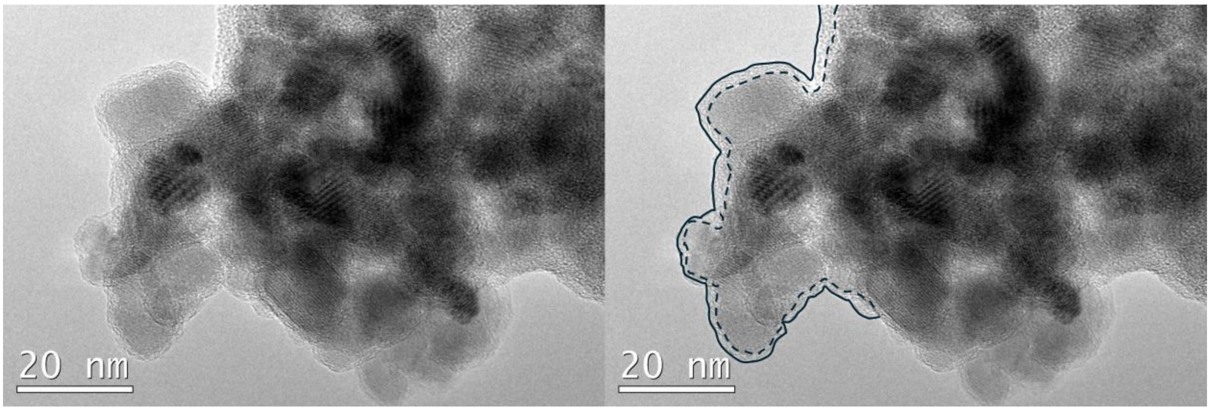

Figure S52. High-magnification TEM image of **2** recovered after the catalytic reaction.

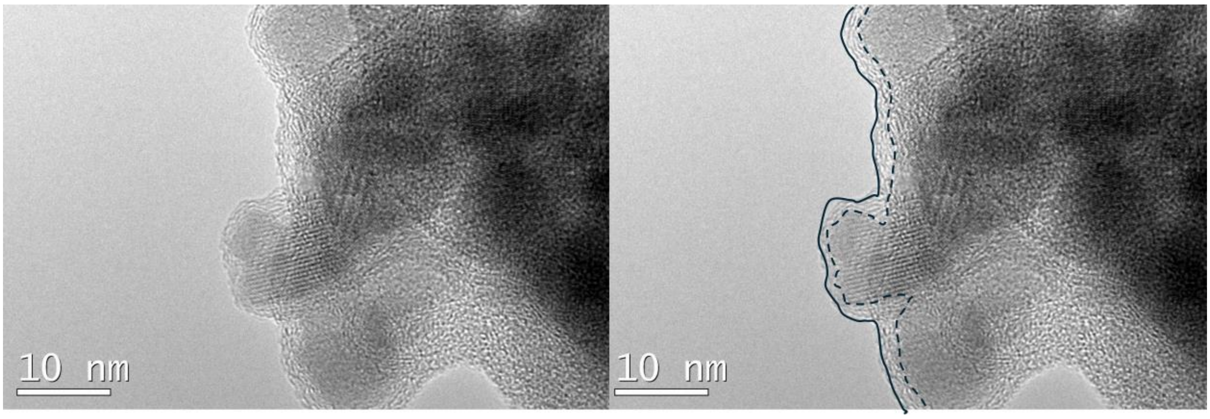

Figure S53. STEM-XEDS elemental map over a group of NPs of **2** recovered after the catalytic reaction.

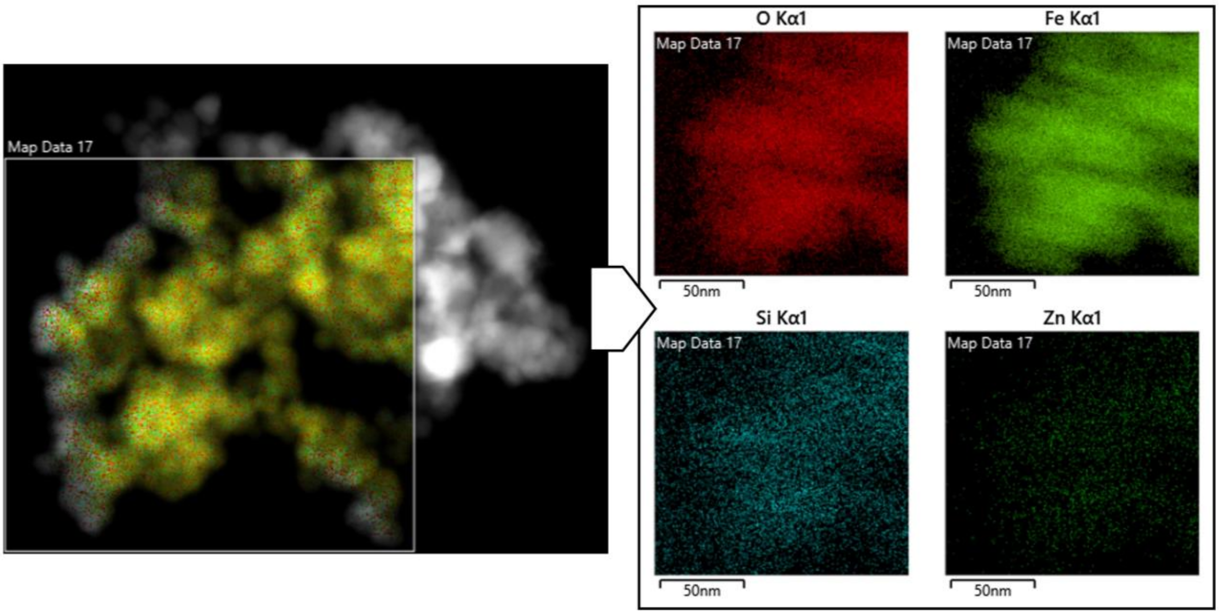

Figure S54. STEM-XEDS elemental map over a group of NPs of **2** recovered after the catalytic reaction.

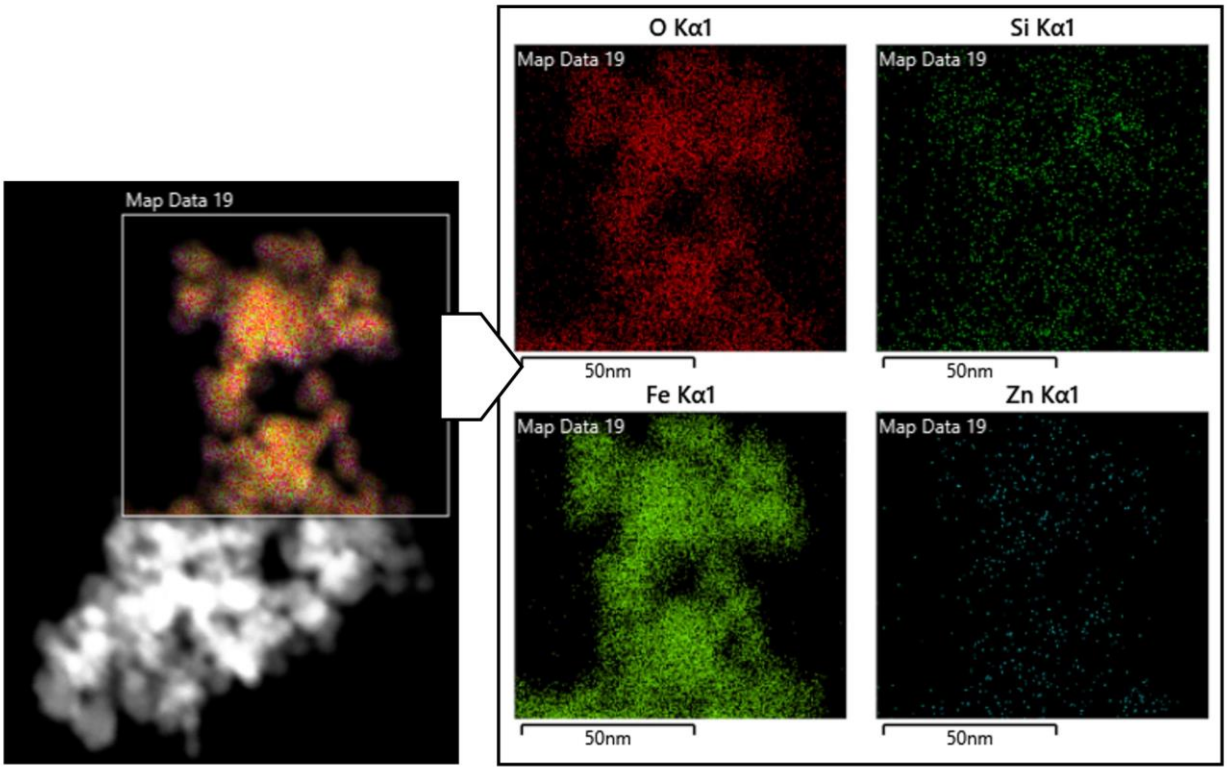

Figure S55. XPS data fitting for (a) Zn ($2p_{2/3}$), (b) Cl (2p), (c) C (1s) and (d) O (1s) lines in the fresh (F, up) and recovered (R, down) catalyst after heating at 180 ºC in the presence of ethylenglycol and washing with CH_2Cl_2. Grey line for background end envelope omitted for clarity in all spectra.

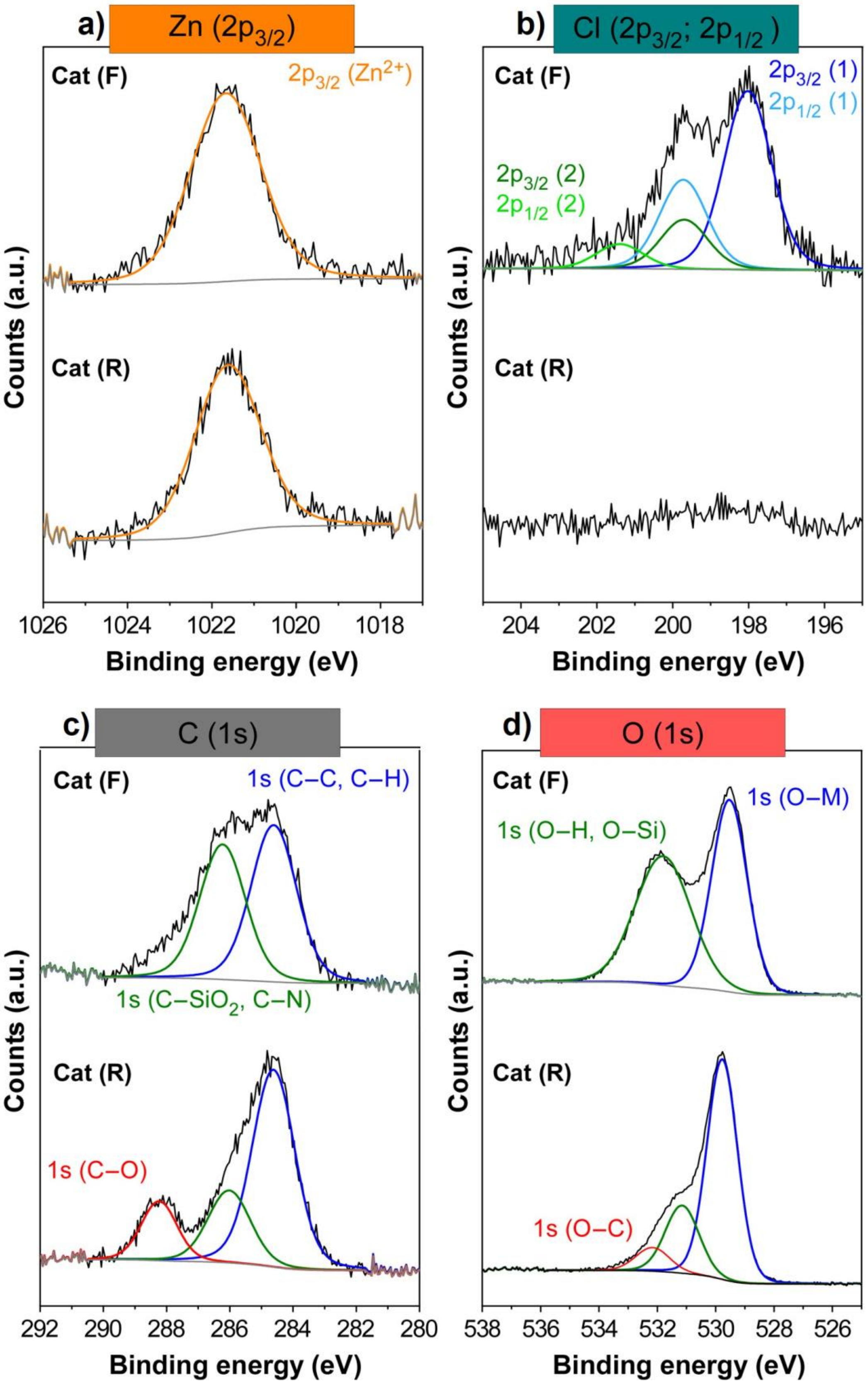

Figure S56. XPS data fitting for Zn ($2p_{2/3}$) and C (1s) in **2** after the catalytic reaction and washing with water. Grey line for background and envelope omitted for clarity.

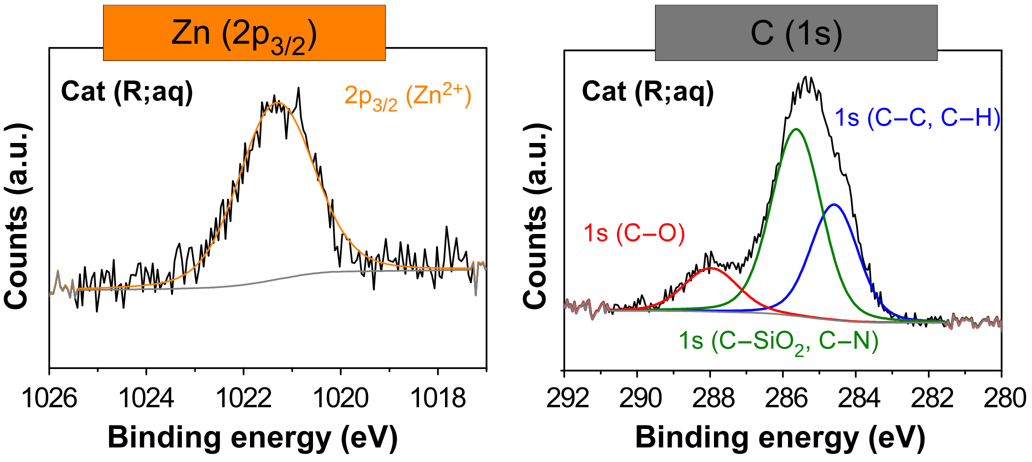

Figure S57. Comparative TGA analyses of Fe_3O_4@SiO_2@(mim)[ZnCl(OH)$_2$] (red trace) and the recovered catalyst after depolymerization reaction at 180 ºC (black trace). Inset: TGA curve of BHET.

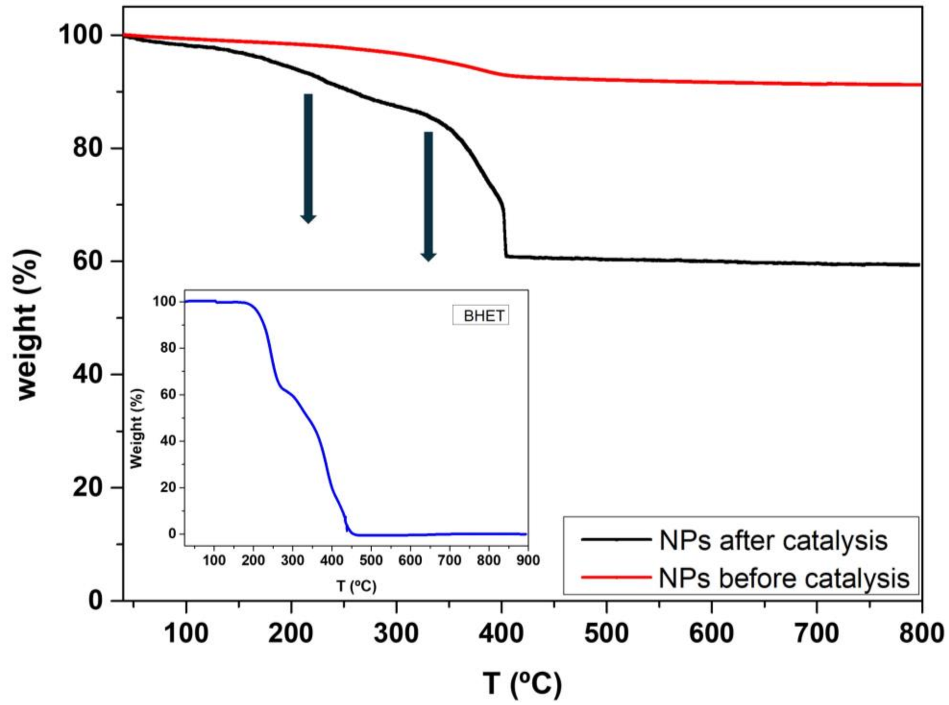

* TGA results show the organic material adsorption from the degradation process (monomers and oligomers) onto the NP surface.

Figure S58. Comparative TGA analyses of Fe_3O_4@SiO_2@(mim)[ZnCl(OH)$_2$] (red trace) and the recovered catalyst after heating at 180 ºC in the presence of EG and washing with CH_2Cl_2 (black trace).

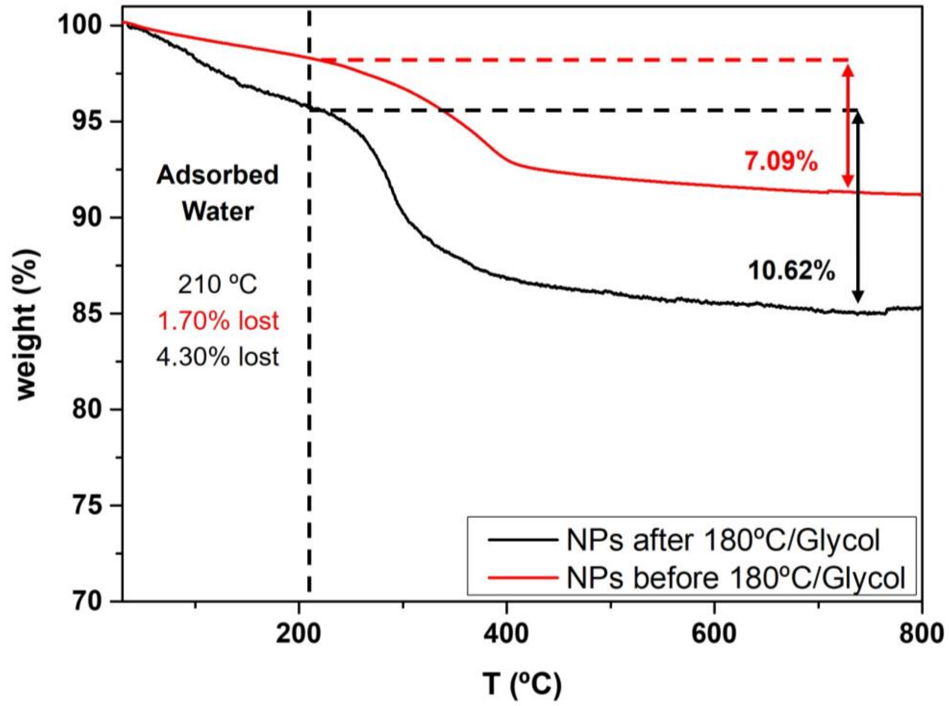

Figure S59. Comparative IR spectra of **2** before (red trace) and after (black trace) the catalytic reaction. Inset: IR spectrum of BHET.

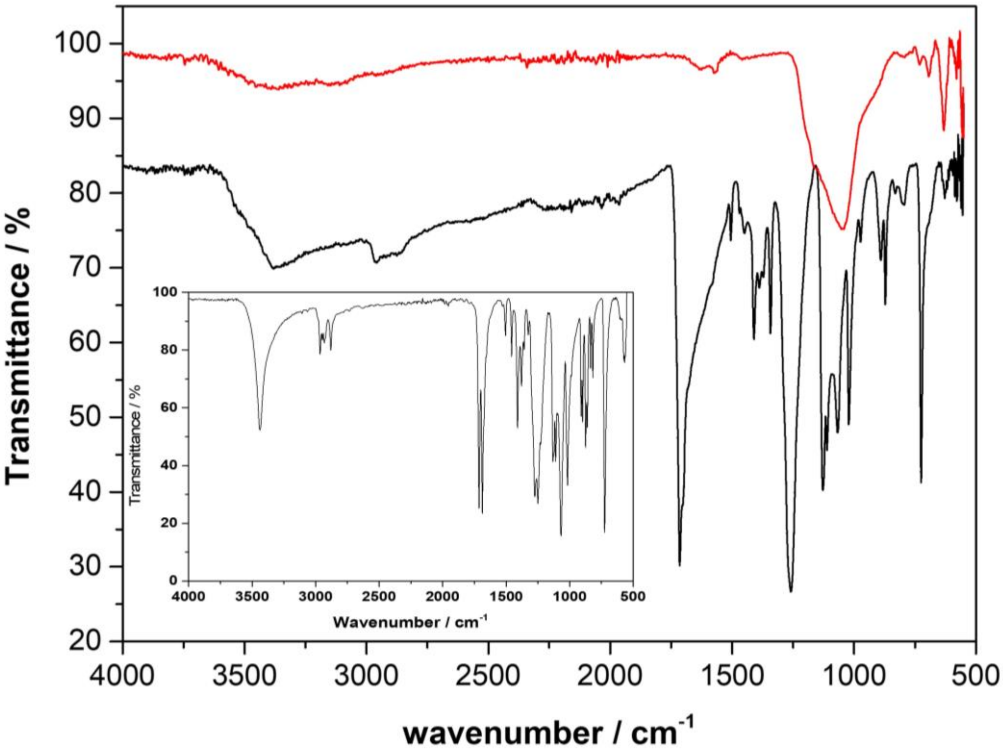

* IR spectrum shows the organic material adsorption from the degradation process (monomers and oligomers) onto the NP surface.

13. Synthesis and characterization of (bmim)[ZnCl(OH)$_2$] and (bmim)[Zn((O)C$_2$H$_4$OH)$_3$]

Synthesis of 1-butyl-3-methylimidazolium zincate, (bmim)[ZnCl(OH)$_2$]. 195 mg (1.4 mmol) of $ZnCl_2$ were dissolved in 60 mL of water and mixed with 250 mg (1.4 mmol) of 1-butyl-3-methylimidazolium chloride. After stirring at 30 ºC for 48 h, the solvent was removed using a rotary evaporator at 40 °C. The ionic liquid (bmim)[ZnCl(OH)$_2$] was obtained in quantitative yield. Anal. calcd. for $C_8H_{17}ClN_2O_2Zn$: C 35.06, H 6.25, N 10.22; found: C 34.88, H 5.56, N 9.11

***In situ* generation of (bmim)[Zn((O)C$_2$H$_4$OH)$_3$].** 22 mg (0.07 mmol) of (bmim)[ZnCl(OH)$_2$] were mixed with an excess of ethylene glycol (11 μL, 0.20 mmol). After stirring at 170 ºC for 24 h, the substitution of Cl^- and OH^- ions by glycolate species (understood as the anion formed after deprotonation of EG) occurred, affording the (bmim)[Zn($C_2H_4O_2$)$_n$] IL. Note that the signals integration in the ^{1}H spectrum indicates the coordination of three EG units to the Zn atom. Accordingly, the formula (bmim)[Zn((O)C_2H_4OH)$_3$] was proposed for a correct charge balancing.

Figure S60. ^{1}H NMR spectrum of (bmim)Cl (300 MHz, 298 K, $CDCl_3$).

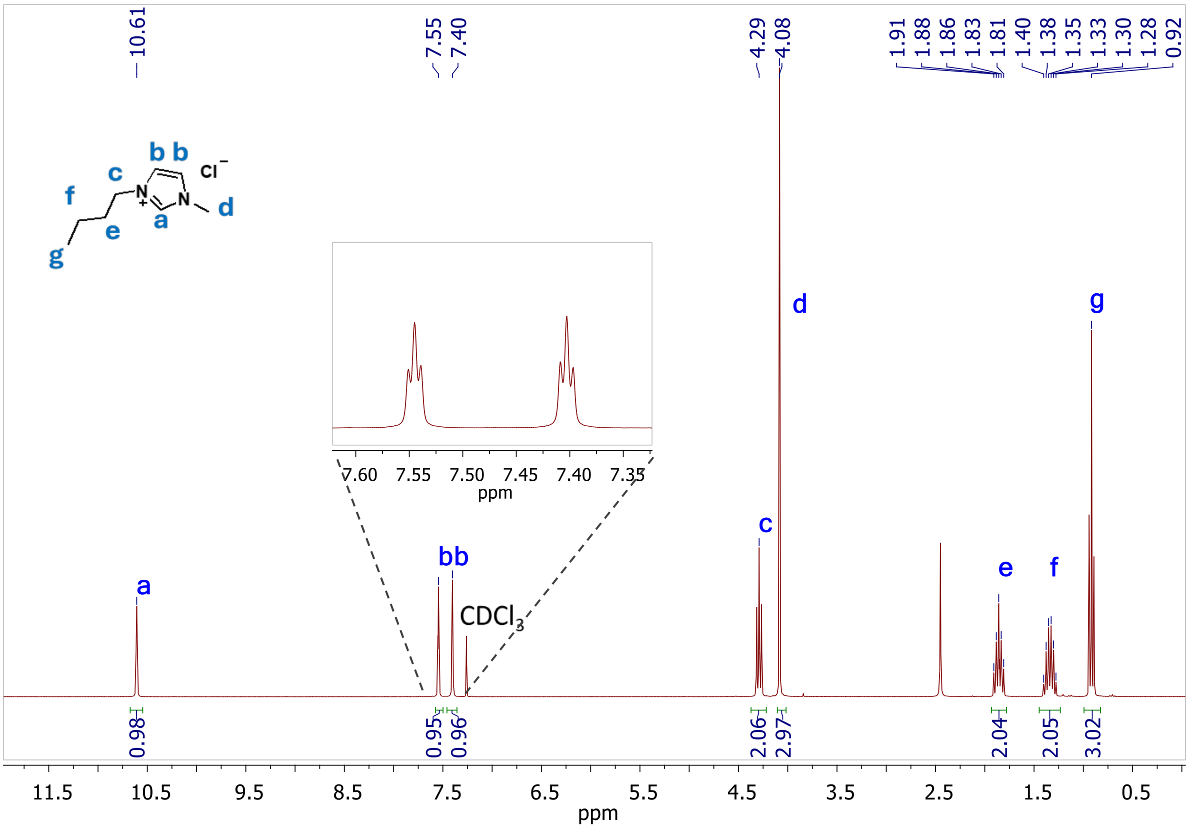

^{1}H NMR (300 MHz, 298 K, $CDCl_3$): δ 10.61 (s, 1H, N_2CH_{Ar}), 7.40 (dd, J = 3.6, 1.7 Hz, 1H, NCH_{Ar}), 7.26 (dd, J = 3.6, 1.7 Hz, 1H, NCH_{Ar}), 4.26 (t, J = 7.1 Hz, 2H, –$NCH_2CH_2CH_2CH_3$), 4.08 (s, 3H, –NCH_3), 1.86 (m, 2H, –$NCH_2CH_2CH_2CH_3$), 1.34 (m, 2H, –$NCH_2CH_2CH_2CH_3$), 0.92 (t, J = 7.3 Hz, 3H, –$NCH_2CH_2CH_2CH_3$).

*(bmim)Cl (bmim = 1-butyl-3-methylimidazolium) was purchased from Sigma Aldrich (ref. 94128-5G and CAS number 79917-90-1).

Figure S61. ^{1}H NMR spectrum of (bmim)[$ZnCl(OH)_2$] (300 MHz, 298 K, $CDCl_3$).

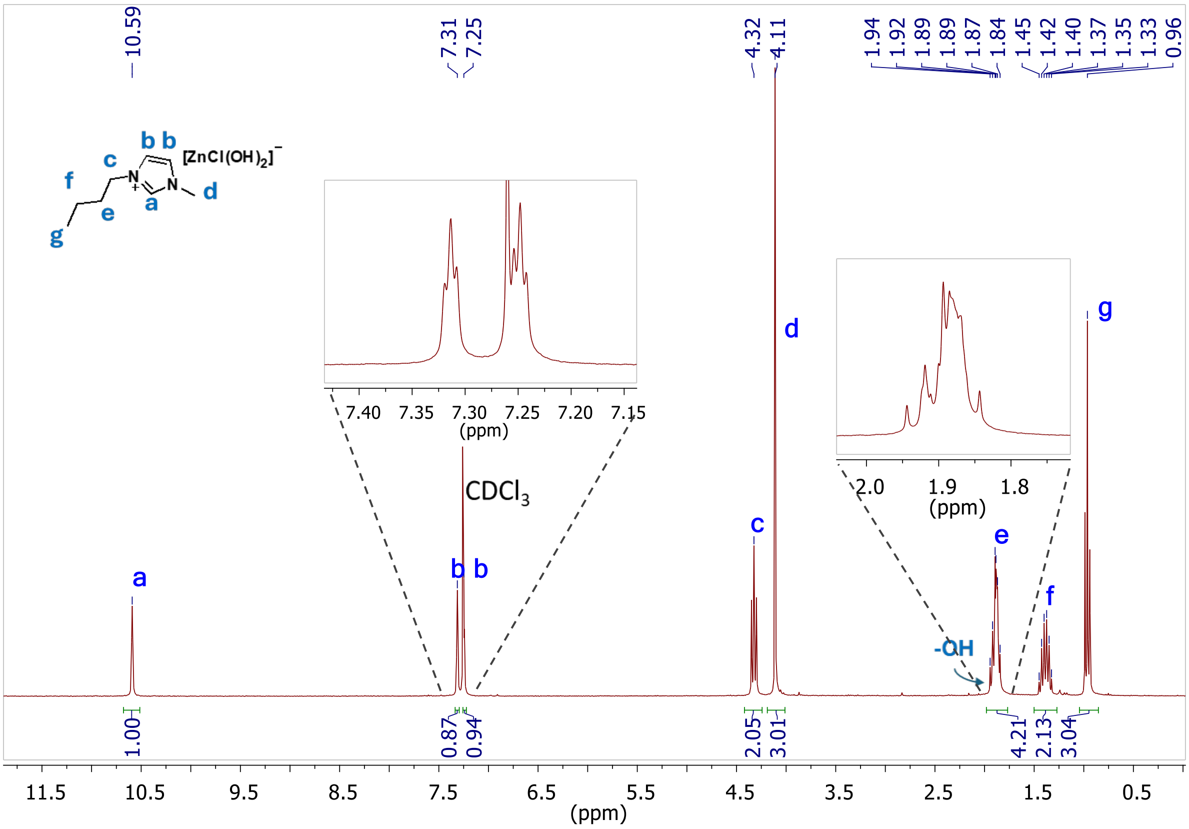

^{1}H NMR (300 MHz, 298 K, $CDCl_3$): δ 10.61 (s, 1H, N_2CH_{Ar}), 7.31 (dd, J = 3.1, 1.7 Hz, 1H, NCH_{Ar}), 7.25 (dd, J = 3.1, 1.7 Hz, 1H, NCH_{Ar}), 4.32 (t, J = 7.4 Hz, 2H, –$NCH_2CH_2CH_2CH_3$), 4.11 (s, 3H, –NCH_3), 1.96-1.84 (m, 4H, –$NCH_2CH_2CH_2CH_3$ and two –OH units), 1.45-1.33 (m, 2H, –$NCH_2CH_2CH_2CH_3$), 0.92 (t, J = 7.1 Hz, 3H, –$NCH_2CH_2CH_2CH_3$).

*Note: Resonances of Zn−OH moieties were observed around 0 ppm for tris(pyrazoly1)hydroboratozinc hydroxide complexes (Tp^xM−OH).[25,26] The observed upfield shift of the peak for the OH^- ligand is a consequence of the Zn−OH^- interaction and the electronic properties of the Tp^x ligand, since the chemical shift of the signal corresponding to coordinated OH^- is correlated with the electron density at the metal center.[27]

Figure S62. ^{13}C NMR spectrum of spectrum of [bmim][$ZnCl(OH)_2$] (75 MHz, 298 K, $CDCl_3$).

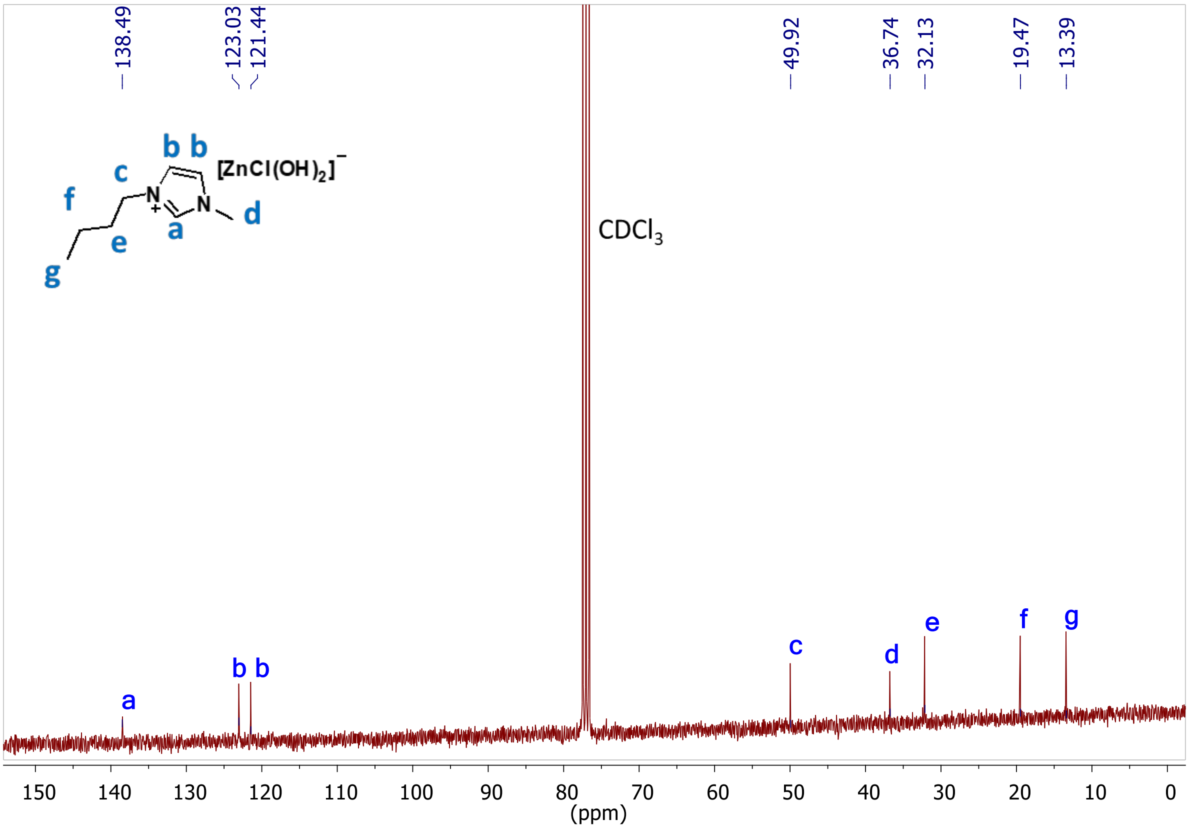

^{13}C NMR (75 MHz, 298 K, $CDCl_3$): δ 138.5 (s, CH, N*CH*$_{Ar}$), 123.0 (s, CH, N*CH*$_{Ar}$), 121.4 (s, CH, N*CH*$_{Ar}$), 49.9 (s, CH_2, N*C*$H_2(CH_2)_2CH_3$), 36.7 (s, CH_3, N*C*H_3), 32.1 (s, CH_2, NCH_2(*C*$H_2)_2CH_3$), 19.4 (s, CH_2, NCH_2(*C*$H_2)_2CH_3$), 13.4 (s, CH_3, $NCH_2(CH_2)_2$*C*H_3).

Figure S63. IR spectrum of [bmim][$ZnCl(OH)_2$].

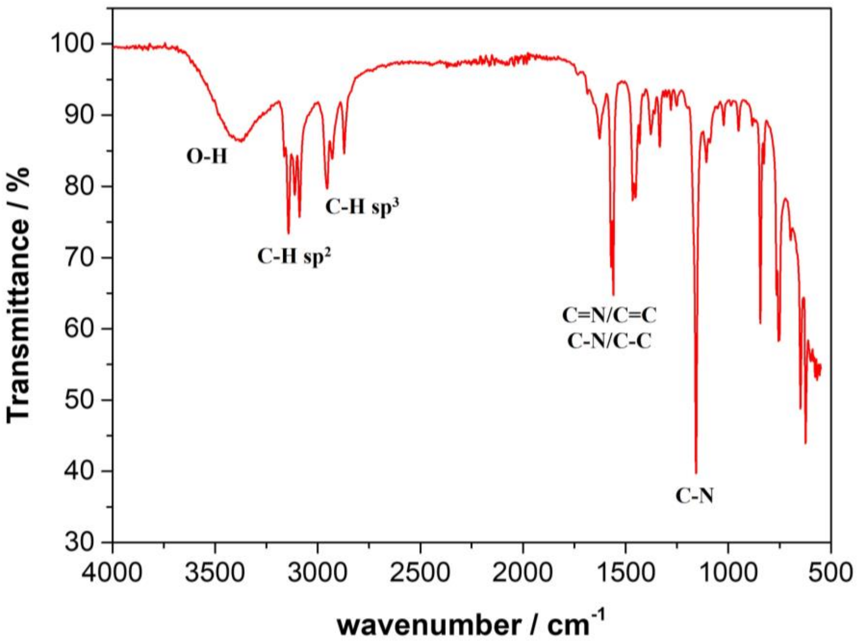

Characteristic IR bands (cm^{-1}): 3391 (O–H), 3163–3087 (C–H sp^2), 2954–2872 (C–H sp^3), 1572 (C=C/C=N), 1561 (C–C/C–N), 1158 (C–N).

Figure S64. Comparative IR spectra of [bmim][$ZnCl(OH)_2$ (red trace) and [bmim]Cl (blue trace).

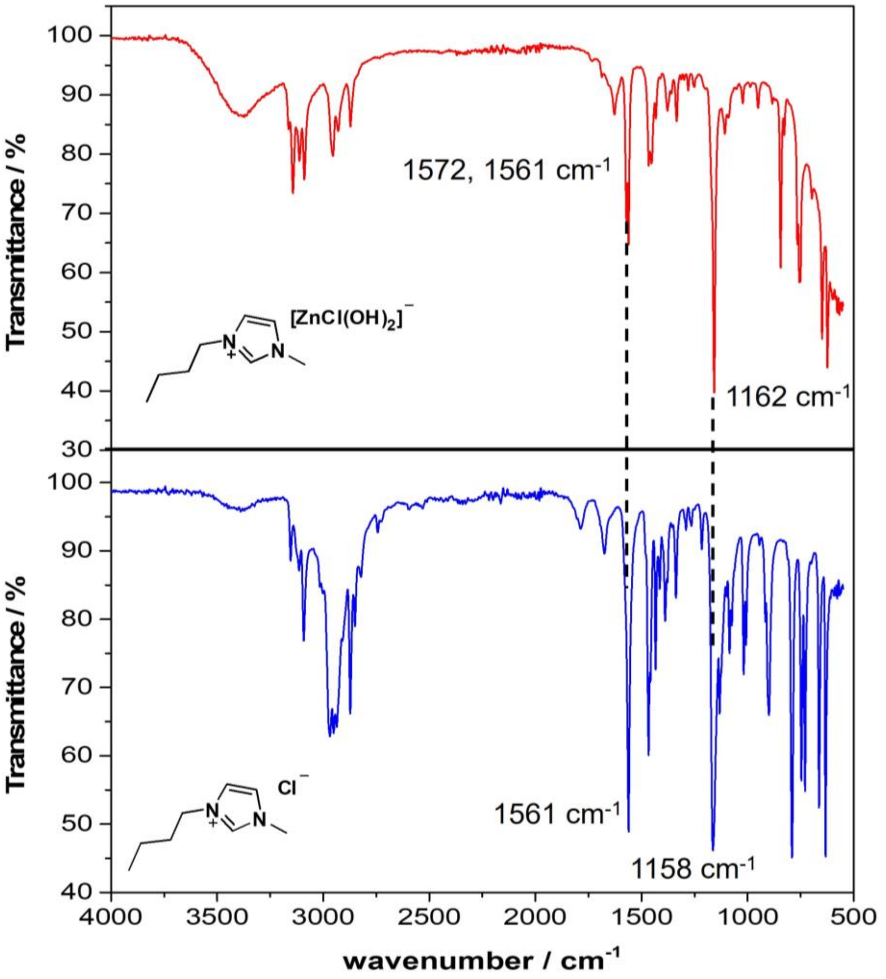

Figure S65. ^{1}H NMR spectrum of (bmim)[ZnCl(OH)$_2$] + ethylene glycol (300 MHz, 298 K, CDCl$_3$).

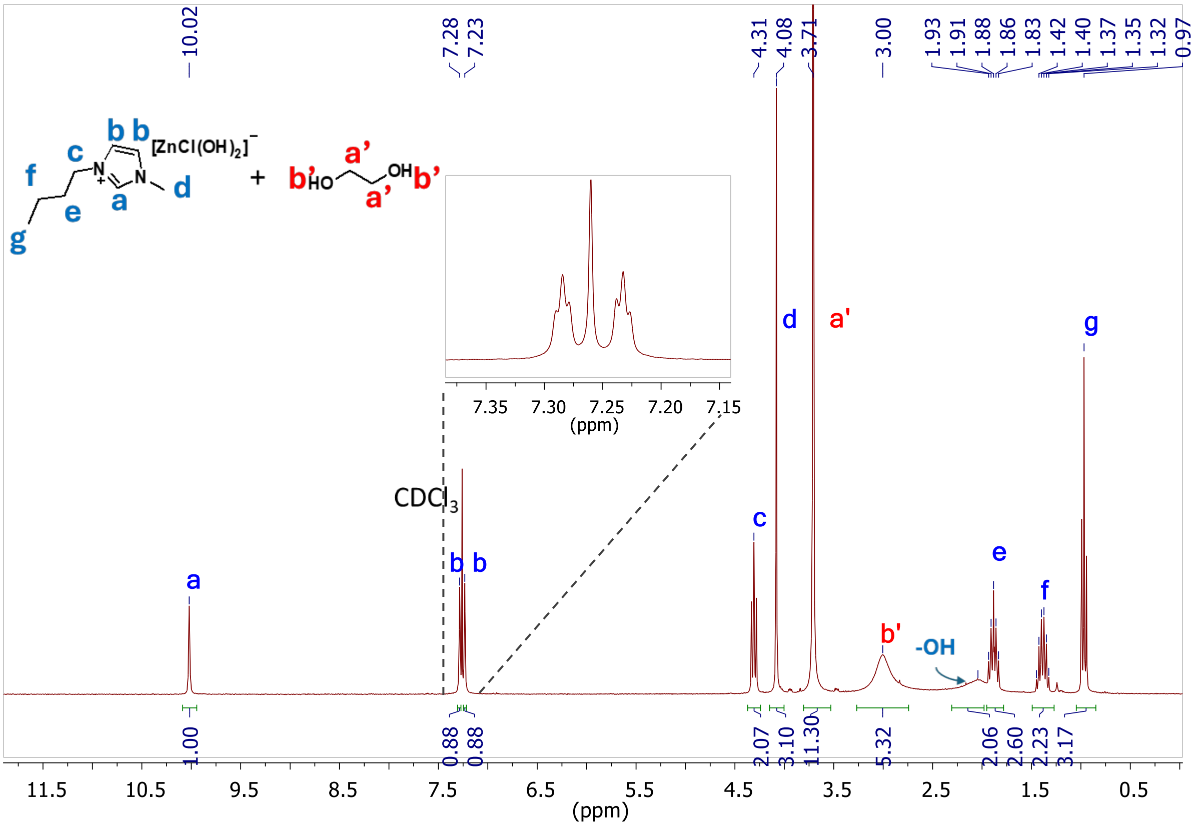

^{1}H NMR (300 MHz, 298 K, CDCl$_3$): δ 10.02 (s, 1H, N$_2$C*H*$_{Ar}$), 7.28 (dd, *J* = 3.4, 1.7 Hz, 1H, NC*H*$_{Ar}$), 7.23 (dd, *J* = 3.4, 1.7 Hz, 1H, NC*H*$_{Ar}$), 4.31 (t, *J* = 7.4 Hz, 2H, –NC*H*$_2$CH$_2$CH$_2$CH$_3$), 4.08 (s, 3H, –NC*H*$_3$), 3.71 (s, 4H, HOC*H*$_2$C*H*$_2$OH), 3.00 (s br, 2H, *H*OCH$_2$CH$_2$O*H*, glycolate), 2.04 (s br, 2H, two –OH units), 1.93-1.83 (m, 2H, –NCH$_2$C*H*$_2$CH$_2$CH$_3$), 1.45-1.32 (m, 2H, –NCH$_2$CH$_2$C*H*$_2$CH$_3$), 0.97 (t, *J* = 7.3 Hz, 3H, –NCH$_2$CH$_2$CH$_2$C*H*$_3$).

Figure S66. ^{1}H NMR spectrum of (bmim)[Zn((O)$C_2H_4OH)_3$] (300 MHz, 298 K, $CDCl_3$).

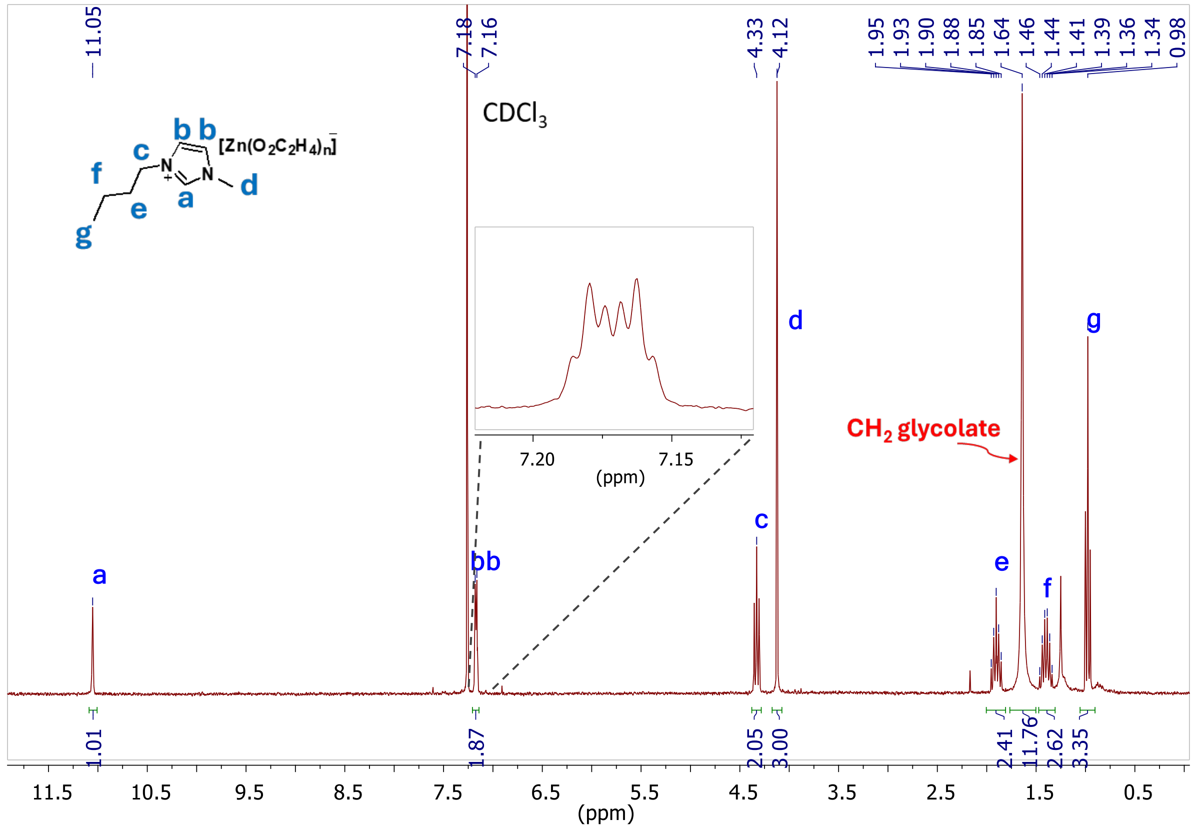

^{1}H NMR (300 MHz, 298 K, $CDCl_3$): δ 11.05 (s, 1H, N_2CH_{Ar}), 7.18 (dd, J = 3.5, 1.8 Hz, 1H, NCH_{Ar}), 7.16 (dd, J = 3.5, 1.8 Hz, 1H, NCH_{Ar}), 4.33 (t, J = 7.4 Hz, 2H, –$NCH_2CH_2CH_2CH_3$), 4.12 (s, 3H, –NCH_3), 1.95-1.85 (m, 2H, –$NCH_2CH_2CH_2CH_3$), 1.64 (s, 4H, –OCH_2CH_2O, glycolate), 1.46-1.34 (m, 2H, –$NCH_2CH_2CH_2CH_3$), 0.98 (t, J = 7.3 Hz, 3H, –$NCH_2CH_2CH_2CH_3$).

14. References

1. Cano, I.; Martín, C.; Fernandes, J. A.; Lodge, R. W.; Dupont, J.; Casado-Carmona, F. A.; Lucena, R.; Cárdenas, S.; Sans, V.; de Pedro, I. Paramagnetic ionic liquid-coated $SiO_2@Fe_3O_4$ nanoparticles—The next generation of magnetically recoverable nanocatalysts applied in the glycolysis of PET. *App. Catal. B Environ.* **2020**, *260*, 118110. DOI: 10.1016/j.apcatb.2019.118110.

2. Brunauer, S.; Emmett, P. H.; Teller, E. Adsorption of Gases Multimolecular Layers. *J. Am. Chem. Soc.* **1938**, *60,* 309–319. DOI: 10.1021/ja01269a023.

3. Thommes, M.; Kaneko, K.; Neimark, A. V.; Oliver, J. P.; Rodriguez-Reinoso, F.; Rouquerol, J.; Sing, K. S. W. Physisorption of gases, with special reference to the evaluation of surface area and pore size distribution (IUPAC Technical Report). *Pure Appl. Chem.*, **2015**, *87,* 1051–1069. DOI: 10.1515/pac-2014-1117.

4. Hajipour, A. R.; Tadayoni, N. S.; Khorsandi, Z. Magnetic iron oxide nanoparticles–N-heterocyclic carbene–palladium(II): a new, efficient and robust recyclable catalyst for Mizoroki–Heck and Suzuki–Miyaura coupling reactions. *Appl. Organomet. Chem.* **2016**, *30*, 590–595. DOI: 10.1002/aoc.3475.

5. Martin, C.; Cano, I.; Scé, F.; Pérez-Aguirre, R.; Gimbert-Suriñach, C.; López-Cornejo, P.; de Pedro, I. Synthesis of Chiral Iron-Based Ionic Liquids: Modelling Stable Hybrid Materials. *New J. Chem.* **2020**, *44*, 6375–6383. DOI: 10.1039/d0nj00349b.

6. Wang, Q.; Geng, Y.; Lu, X.; Zhang, S. First-Row Transition Metal-Containing Ionic Liquids as Highly Active Catalysts for the Glycolysis of Poly(ethylene terephthalate) (PET). *ACS Sustainable Chem. Eng.* **2015**, *3*, 340–348. DOI: 10.1021/sc5007522.

7. Nifant'ev, I. E.; Pyatakov, D. A.; Tavtorkin, A. N.; Ivchenko, P. V. Chemical recycling and upcycling of poly(Bisphenol A carbonate) *via* metal acetate catalyzed glycolysis. *Polym. Degrad. and Stab*. **2023**, *207*, 110210. DOI: 10.1016/j.polymdegradstab.2022.110210.

8. Quaranta, E.; Minischetti, C. C.; Tartaro, G. Chemical Recycling of Poly(bisphenol A carbonate) by Glycolysis under 1,8-Diazabicyclo[5.4.0]undec-7-ene Catalysis. *ACS Omega* **2018**, *3*, 7261–7268. DOI: 10.1021/acsomega.8b01123.

9. Achilias, D. S.; Redhwi, H. H.; Siddiqui, M. N.; Nikolaidis, A. K.; Bikiaris, D. N.; Karayannidis, G. P. Glycolytic depolymerization of PET waste in a microwave reactor, *J. Appl. Polym. Sci.* **2010**, *118*, 3066–3073. DOI: 10.1002/app.32737.

10. Pingale, N.; Shukla, S. Microwave assisted ecofriendly recycling of poly (ethylene terephthalate) bottle waste. *Eur. Polym. J.* **2008**, *44*, 4151–4156. DOI: 10.1016/j.eurpolymj.2008.09.019.

11. Grebowicz, J.S. Thermal properties of polycarbonate grade bisphenol A. *J. Therm. Anal.* **1996**, *46*, 1151–1166. DOI: 10.1007/BF01983626.

12. Wang, T.; Zheng, Y.; Yu, G.; Chen, X. Glycolysis of polyethylene terephthalate: Magnetic nanoparticle $CoFe_2O_4$ catalyst modified using ionic liquid as surfactant. *Eur. Polym. J.* **2021**, *155*, 110590. DOI: 10.1016/j.eurpolymj.2021.110590.

13. Mohammadi, S.; Enayati, M. Magnetic ionic liquid catalyst functionalized with antimony (III) bromide for effective glycolysis of polyethylene terephthalate. *Waste Manag.* **2023**, *170*, 308–316. DOI: 10.1016/j.wasman.2023.09.016.

14. Casey, E.; Breen, R.; Gomez, J. S.; Kentgens, A. P. M.; Pareras, G.; Rimola, A.; Holmes, J. D.; Collins, G. Ligand-Aided Glycolysis of PET Using Functionalized Silica-Supported Fe_2O_3 Nanoparticles. *ACS Sustainable Chem. Eng.* **2023**, *11*, 15544–15555. DOI: 10.1021/acssuschemeng.3c03585.

15. Bartolome, L., Lee, K. G.; Sangalang, A.; Ahn, J. K.; Kim, D. H. Superparamagnetic γ-Fe_2O_3 nanoparticles as an easily recoverable catalyst for the chemical recycling of PET. *Green Chem*. **2014**, *16*, 279–286. DOI: 10.1039/c3gc41834k.

16. Al-Sabagh, A. M.; Yehia, F. Z.; Harding, D. R. K.; Eshaq, G.; ElMetwally, A. E. Fe_3O_4-boosted MWCNT as an efficient sustainable catalyst for PET glycolysis. *Green Chem.* **2016**, *18*, 3997–4003. DOI: 10.1039/C6GC00534A.

17. Du, J.-T.; Wu, H.; Jie, Y.; Xia, Y.; Yang, Z.; Yan, H.; Wang, Q.; Wang, J.-X.; Chen. J.-F. Magnetically recyclable $CoFe_2O_4$ nanocatalysts for efficient glycolysis of polyethylene terephthalate. *Chem. Eng. Sci*. **2025**, *304*, 121042. DOI: 10.1016/j.ces.2024.121042.

18. Guo, Z.; Adolfsson, E.; Tam, P. L. Nanostructured micro particles as a low-cost and sustainable catalyst in the recycling of PET fiber waste by the glycolysis method. *Waste Manag.* **2021**, *126*, 559–566. DOI: 10.1016/j.wasman.2021.03.049.

19. Thomas, D.; Ranjana, R.; George, B. K. Co-Al-CO_3 layered double hydroxide: an efficient and regenerable catalyst for glycolysis of polyethylene terephthalate. *RSC Sustain.* **2023**, *1*, 2277–2286. DOI: 10.1039/d3su00304c.

20. Liu, Y.; Yao, X.; Yao, H.; Zhou, Q.; Xin, J.; Lu, X.; Zhang, S. Degradation of poly(ethylene terephthalate) catalyzed by metal-free choline-based ionic liquids. *Green Chem.* **2020**, *22*, 3122–3131. DOI: 10.1039/D0GC00327A.

21. Al-Sabagh, A. M.; Yehia, F. Z.; Eshaq, Gh.; ElMetwally, A. E. Ionic Liquid-Coordinated Ferrous Acetate Complex Immobilized on Bentonite as a Novel Separable Catalyst for PET Glycolysis. *Ind. Eng. Chem. Res.* **2015**, *54*, 12474–12481. DOI: 10.1021/acs.iecr.5b03857.

22. Scé, F.; Cano, I.; Martin, C.; Beobide, G.; Castillo, Ó.; de Pedro, I. Comparing conventional and microwave-assisted heating in PET degradation mediated by imidazolium-based halometallate complexes. *New J. Chem.* **2019**, *43*, 3476–3485. DOI: 10.1039/C8NJ06090H.

23. Cot, S.; Leu, M. K.; Kalamiotis, A.; Dimitrakis, G.; Sans, V.; de Pedro, I.; Cano, I. An Oxalate-Bridged Binuclear Iron(III) Ionic Liquid for the Highly Efficient Glycolysis of Polyethylene Terephthalate under Microwave Irradiation. *ChemPlusChem* **2019**, *84*, 786–793. DOI: 10.1002/cplu.201900075.

24. Zhou, X.; Lu, X.; Wang, Q.; Zhu, M.; Li, Z. Effective catalysis of poly(ethylene terephthalate) (PET) degradation by metallic acetate ionic liquids. *Pure Appl. Chem.* **2012**, *84*, 789–801. DOI: 10.1351/PAC-CON-11-06-10.

25. Sattler, W.; Parkin, G. Low temperature NMR spectroscopic investigation of a zinc bicarbonate compound: Thermodynamics of bicarbonate formation by insertion of CO_2 into the zinc hydroxide bond of [TpBut;Me]ZnOH. *Polyhedron* **2012**, 3241–3248. DOI: 10.1016/j.poly.2011.08.015.

26. Looney, A.; Han, R.; McNeill, K.; Parkin, G. Tris(pyrazoly1)hydroboratozinc Hydroxide Complexes as Functional Models for Carbonic Anhydrase: On the Nature of the Bicarbonate Intermediate. *J. Am. Chem. Soc.* **1993**, *115*, 4690–4697. DOI: 10.1021/ja00064a033.

27. Martín, C.; Muñoz-Molina, J. M; Locati, A.; Alvarez, E.; Maseras, F.; Belderrain, T. R.; Pérez, P. J. Copper(I)-Olefin Complexes: The Effect of the Trispyrazolylborate Ancillary Ligand in Structure and Reactivity. *Organometallics* **2010**, *29*, 3481–3489. DOI: 10.1021/om1002705.
